# Biodiversity, Evolution and Ecological Specialization of Baculoviruses: A Treasure Trove for Future Applied Research

**DOI:** 10.3390/v10070366

**Published:** 2018-07-11

**Authors:** Julien Thézé, Carlos Lopez-Vaamonde, Jenny S. Cory, Elisabeth A. Herniou

**Affiliations:** 1 Institut de Recherche sur la Biologie de l’Insecte, UMR 7261, CNRS—Université de Tours, 37200 Tours, France; julien.theze@zoo.ox.ac.uk (J.T.); carlos.lopezvaamonde@inra.fr (C.L.-V.); 2 Department of Zoology, University of Oxford, South Parks Road, Oxford OX1 3SY, UK; 3 INRA, UR633 Zoologie Forestière, 45075 Orléans, France; 4 Department of Biological Sciences, Simon Fraser University, Burnaby, BC V5A 1S6, Canada; jennifer_cory@sfu.ca

**Keywords:** nucleopolyhedrovirus, granulovirus, lepidoptera, phylogenetics, species delimitation, niche conservatism, host shifts, cophylogeny, resource tracking, multitrophic interactions

## Abstract

The *Baculoviridae*, a family of insect-specific large DNA viruses, is widely used in both biotechnology and biological control. Its applied value stems from millions of years of evolution influenced by interactions with their hosts and the environment. To understand how ecological interactions have shaped baculovirus diversification, we reconstructed a robust molecular phylogeny using 217 complete genomes and ~580 isolates for which at least one of four lepidopteran core genes was available. We then used a phylogenetic-concept-based approach (mPTP) to delimit 165 baculovirus species, including 38 species derived from new genetic data. Phylogenetic optimization of ecological characters revealed a general pattern of host conservatism punctuated by occasional shifts between closely related hosts and major shifts between lepidopteran superfamilies. Moreover, we found significant phylogenetic conservatism between baculoviruses and the type of plant growth (woody or herbaceous) associated with their insect hosts. In addition, we found that colonization of new ecological niches sometimes led to viral radiation. These macroevolutionary patterns show that besides selection during the infection process, baculovirus diversification was influenced by tritrophic interactions, explained by their persistence on plants and interactions in the midgut during horizontal transmission. This complete eco-evolutionary framework highlights the potential innovations that could still be harnessed from the diversity of baculoviruses.

## 1. Introduction

The use of baculoviruses (BVs) as expression vectors has mainly focused on the development of a single virus, namely *Autographa californica multiple nucleopolyhedrovirus* (AcMNPV) [1], and also, to a lesser extent, *Bombyx mori nucleopolyhedrovirus* (BmNPV) [2]. The commercial success of AcMNPV for biotechnological application is undeniable. Aside from historical reasons, the fact that it is a generalist virus that can infect cell lines from different hosts probably explains why AcMNPV was first chosen. The family *Baculoviridae*, however, encompasses hundreds of isolates, many of which have been studied in the context of biological control of insect pests, but some of which could in the future prove equally as useful as AcMNPV for biotechnological applications, by providing new molecular and biochemical products with contrasting antigenic properties or by infecting new, more productive cell lines. In this context, it is important to describe the taxonomical diversity of BVs to ensure that new isolates developed for biotechnological products are not examples of commonly used viral species, which could infringe patents. However, to delineate species boundaries requires an understanding of BV evolution based on both historical and ecological perspectives.

The delimitation of species is a notoriously difficult question in biology, particularly for those organisms for which the biological species concept cannot be applied. Viral species are defined by the International Committee on Taxonomy of Viruses (ICTV) as “a monophyletic group of viruses whose properties can be distinguished from those of other species by multiple criteria” [3]. This general definition recognizes the importance of shared evolutionary history to define viral lineages but allows for other biological criteria to be used, whether or not they may have an evolutionary meaning. Recent advances in virus discovery (e.g., [4]) have spurred the need to reconsider the method used for virus classifications [5,6]. Genetic distances are commonly used to delimitate viral species based on distance cut-offs [5]. In the case of BVs, the cut-off between species has been defined based on sequence alignments of the *polyhedrin (polh)*, *late expression factor 8 (lef-8)* and *late expression factor 9 (lef-9)* genes as between 0.015 and 0.05 using Kimura-2-parameter distances [7], which are genetic distances taking into account differential substitution rates for transitions and transversions, with the assumption of equal nucleotide frequencies and equal substitution rate among sites. There is no doubt about the usefulness of those distance thresholds to cluster closely related isolates (distance below 0.015) and to separate distantly related viruses. However, there is often a high degree of uncertainty when clustering at intermediate levels of genetic distances (up to 0.05). Intermediate levels of genetic differentiation could be found within widespread, generalist, or fast evolving viral species. In these cases, the sole use of genetic distances is insufficient to infer clusters and the delimitation criteria are left to the judgment of the taxonomist. There is therefore a need to consider alternative analyses for the delimitation of BV species, which could also be applied to other viral families. Recent advances in molecular phylogenetics now allow the clustering of sequences into species groups, taking into account differences in evolutionary rates or based on coalescence theory (e.g., [8,9]), and which do not require prior knowledge of species limits. These automated methods, designed for DNA barcoding (e.g., [10,11]) to group individual animal sequences of typically one gene into species clusters, are based on universal theory and can therefore be applied to other genes and taxa [12], including viruses.

As insect-specific, enveloped, rod-shaped viruses with large circular double-stranded DNA genomes, BVs replicate in the nucleus of infected host cells. Their life cycle is characterized by the packaging of virions within a matrix of protein, forming the so-called occlusion body (OB), which is the vehicle of horizontal transmission. Over 600 BVs have been described from the insect orders Lepidoptera, Hymenoptera and Diptera. More than 90% of the known BVs have been isolated from lepidopteran hosts [13]. The current classification [14] divides the *Baculoviridae* family into four genera [15]. Two genera, the *Alphabaculovirus* and *Betabaculovirus*, infect lepidopteran species, and differ by the morphology of their OBs forming the nucleopolyhedroviruses (NPVs) and granuloviruses (GVs) respectively. The OBs of NPVs contain many virions and have been isolated from both lepidopteran and non-lepidopteran hosts; in contrast, the OBs of the lepidopteran GVs contain a single virion [14]. Phylogenetic and comparative genomic studies have shown that both Lepidoptera specific genera are sister groups and that they diversified during the Mesozoic Era after the diversification of insect orders [7,16,17,18].

OBs are the between host transmission stage of the virus and allow the virus to survive outside the host [19]. BVs are generally obligate killers, as the host must die before the OBs are released into the environment for horizontal transmission. However, latent BV infections [20,21] and vertical transmission [22,23,24] have been reported. Insect larvae become infected by ingesting host plant tissue contaminated with OBs. Thus, plant and virus have a close interaction in the insect gut and plant chemicals can interact with the virus in numerous ways resulting in altered infectivity [25]. As a result of these tritrophic interactions (meaning the ecological impact on each other of three levels within a single food chain, i.e., here between the entomopathogenic virus, the insect host and the insect host plant), there is the potential for plants to play an important role in the evolution of insect-BV interactions [25,26,27,28]. A better understanding of these diverse evolutionary interactions might point to new molecular targets that could lead to biotechnological innovations.

In this study, our main aims were to (1) provide a robust phylogeny for the whole family *Baculoviridae* using all the genetic data generated so far; (2) delineate BV species using a non-a-priori phylogenetic-concept-based approach; (3) study the evolutionary history of host use of BVs, assessing the level of host specialization and role of host shifts in the speciation of BVs; (4) elucidate the role of host plants in the evolution of BVs. Our results shed light on the biodiversity ecological and evolutionary factors that drive *Baculoviridae* diversification.

## 2. Materials and Methods

### 2.1. Virus Isolate Sequence Database

We created a DNA sequence database containing sequences of at least one of the four lepidopteran BV core genes, *lef-8, lef-9, per os infectivity factor 2 (pif-2)* and *polh.* We chose those genes because it has been shown that they bear a strong phylogenetic signal [16] and they have been abundantly sequenced. The database includes sequences collated from the public databases GenBank, EMBL and DDBJ (version April 2018), and we tried to obtain sequences from one hundred historical BV samples originating from the reference collection at the Centre for Ecology and Hydrology (NERC-CEH), Wallingford, UK. Primers, PCR amplification protocols and Sanger sequencing are previously described [7,15]. Following current taxonomic practice, each BV sample was associated with the host species from which it was isolated and OB morphology (NPV or GV), as well as sampling information about the virus isolate or strain (if provided), and the name of the first author of the study from which BV sequences derived (Table S1).

### 2.2. Host Ecology Database

For each BV isolate studied, we associated host ecological data. We included the taxonomy of each insect host species (superfamily, family, subfamily), and their geographical distribution (ecozone), established from localities where insect hosts were observed in the field. We also included the host plant range of each insect host, from which we determined the associated insect host plant growth type, distinguishing woody perennial (including shrubby and suffrutescent plants) and annual/biennial herbaceous plants. Information was mainly extracted from the database of the world’s lepidopteran host plants of the Natural History Museum of London [29], from the Barcode of Life Data System (BOLD) [30] and from the literature [31] (Table S2).

### 2.3. Baculovirus Core-Genome Phylogeny

A phylogenomic approach was used to reconstruct the BV core-genome phylogeny. BVs possess 37 core genes [32], identified in all completely sequenced genomes. Amino acid multiple alignments were performed on the 37 BV core gene products using MAFFT program [33] and, alignments were concatenated prior to the phylogenetic reconstruction. A maximum likelihood (ML) phylogenetic inference was performed on the concatenated multiple alignment under the Le and Gascuel amino acid substitution model, with a gamma distributed among site rate variation and a proportion of invariant sites (LG + Γ + I), as determined by ProtTest 3 [34]. The ML analysis was performed with RAxMLv.8.2 program [35] and statistical support for nodes in the ML tree was assessed using a bootstrap approach (with 100 replicates).

### 2.4. Baculovirus Isolate Phylogeny

For each of the four BV core genes (*lef-8*, *lef-9*, *pif-2* and *polh*) codon-based multiple alignments were performed on all BV isolate sequences. Alignments were then concatenated and completed with gaps for isolates, for which up to three of the four lepidopteran core genes might be missing. A ML phylogenetic inference was performed on the concatenated codon-based multiple alignment with RAxMLv.8.2 using the BV core-genome phylogeny as backbone constraint tree. The BV isolate phylogeny was reconstructed using the best-fitted substitution model and parameters GTR + Γ + I, as determined by jModelTest 2 [36] and statistical support for nodes in the ML tree was assessed using a bootstrap approach (with 100 replicates).

### 2.5. Species Delimitation

A species delimitation analysis was performed on the BV isolate phylogeny using a phylogenetic-species-concept-based method, the multi-rate Poisson tree processes (mPTP) model [37]. The mPTP method is an improved version of the PTP model [9], which models speciation or branching events in terms of number of substitutions and uses heuristic algorithms to identify the most likely classification of branches into population and species-level processes. Moreover, mPTP is fast and incorporates different levels of intraspecific genetic diversity deriving from differences in either the evolutionary history or sampling of each species [37]. The mPTP model delimit species on phylogenies without a priori assumptions, instead of the genetic distance cut-off commonly used. The mPTP results were compared to the commonly used genetic-distances-based method. A previous study calculated an intra-species genetic distance of ≤0.015 (up to 0.05 with complementary information) as marker to delimit BV species [7]. This distance is commonly used by the BV taxonomists and was evaluated using the Geneious plugin SpDelim [38], which assesses the within and between species genetic distances in a phylogenetic tree. For both mPTP and SpDelim analyses, the BV isolate ML phylogeny was used.

### 2.6. Baculovirus Species Phylogeny

From the species delimitation analysis, one isolate per cluster was selected as a representative of a putative BV species (e.g., isolates with a complete genome or isolates with the four core genes sequenced) and singletons were considered as distinct putative BV species. The topology of these species-representative isolates was extracted from the BV isolate phylogeny by pruning the other taxa, using the ‘ape’ package [39] for R. A new amino acid multiple alignment was created with only the set of the species-representative isolates. A Bayesian phylogenetic inference was performed on this alignment using BEASTv.1.8.4 [40]. The substitution model and parameters LG + Γ + I were used as well as a fixed strict clock of 1.0. The topology of the above species-representative isolate tree was used as target tree for the calculation of the consensus tree and posterior probabilities.

### 2.7. Phylogeny-Trait Correlation and Ancestral State Estimations

Correlations between phylogenetic tree structure and isolate trait discrete values (see Table S2) were assessed using the methods implemented in BaTS/befi-BaTS [41]. The number of state randomizations was defined as 100 to yield a null distribution. A correlation was considered unambiguously positive if the Association Index (AI), the Parsimony Score (PS), the Phylogenetic Diversity (PD), the Nearest Taxa (NT) and the Net Relatedness (NR) indices, the Unique Fraction (UniFrac) index, and the maximum monophyletic clade (MC) size probabilities were ≤0.01. Phylogenetic uncertainty was taken into account by using the set of tree topologies estimated by the above Bayesian phylogenetic inference. For those traits that obtained significant *p* value, a new Bayesian phylogenetic inference was performed on the species-representative isolate alignment with the same set of parameters as above and adding discrete trait partitions to reconstruct ancestral states.

### 2.8. Host-Virus Cophylogeny

To test for the existence of statistically significant topological congruence between BV species and Lepidoptera hosts, we estimated the maximum number of cospeciation events at different taxonomic levels (superfamily, family, subfamily and genus), needed for reconciling BV species and host phylogenies in TreeMap 3b [42] and compared this estimate to the distribution of corresponding values obtained by randomizing different Lepidoptera trees 100 times while keeping BV-host associations unchanged [43]. Tests at superfamily, family and subfamily taxonomic levels compared the whole BV species phylogeny to published Lepidoptera phylogenies [44,45,46]. Tests at the genus level were performed by reconstructing lepidopteran host species phylogenies based on published phylogenies (Noctuidae [47], Erebidae [48,49], Lasiocampidae [50], *Spodoptera* genus [51]) or when not available on CO1 sequence data extracted from BOLD public database [30] and compared to the BV species tree simplified by pruning monophyletic clades of species sharing a given host genus down to a single species. Analyses were performed using 25 starts from random maps and heuristic searches for up to 25 generations.

## 3. Results

### 3.1. Baculovirus Phylogeny

Here we present a comprehensive phylogeny of BVs, including most of the relevant BV genetic data available to date. A data-mining analysis was performed on public genetic databases and resulted in the collation of 749 BV isolates properly formatted, containing the nucleotide sequences of at least one of four lepidopteran BV core genes (*lef-8*, *lef-9*, *pif-2* and *polh* genes). We were also able to determine the sequences (38 *lef-8*, 39 *lef-9*, 21 *pif-2* and 45 *polh*) for 45 historical isolates from our own collection. The 143 sequences were deposited in the GenBank database under the accession numbers MH454109-MH454230 and MH458171-MH458191. Overall, our working sequence database contains 2053 nucleotide sequences (564 *lef-8* genes, 498 *lef-9* genes, 283 *pif-2* genes and 708 *polh* genes) belonging to 794 BV isolates (Table S1). A total of 235 isolates have the sequences of the four genes.

**Figure 1:**
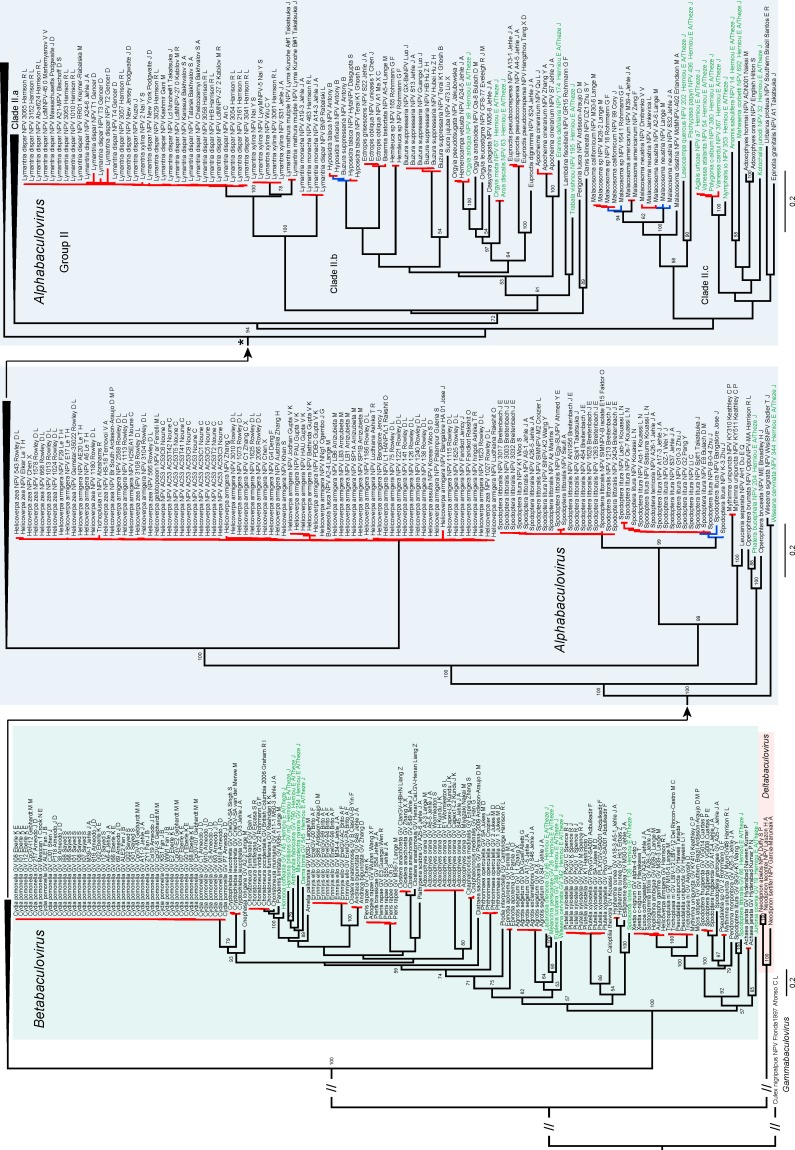

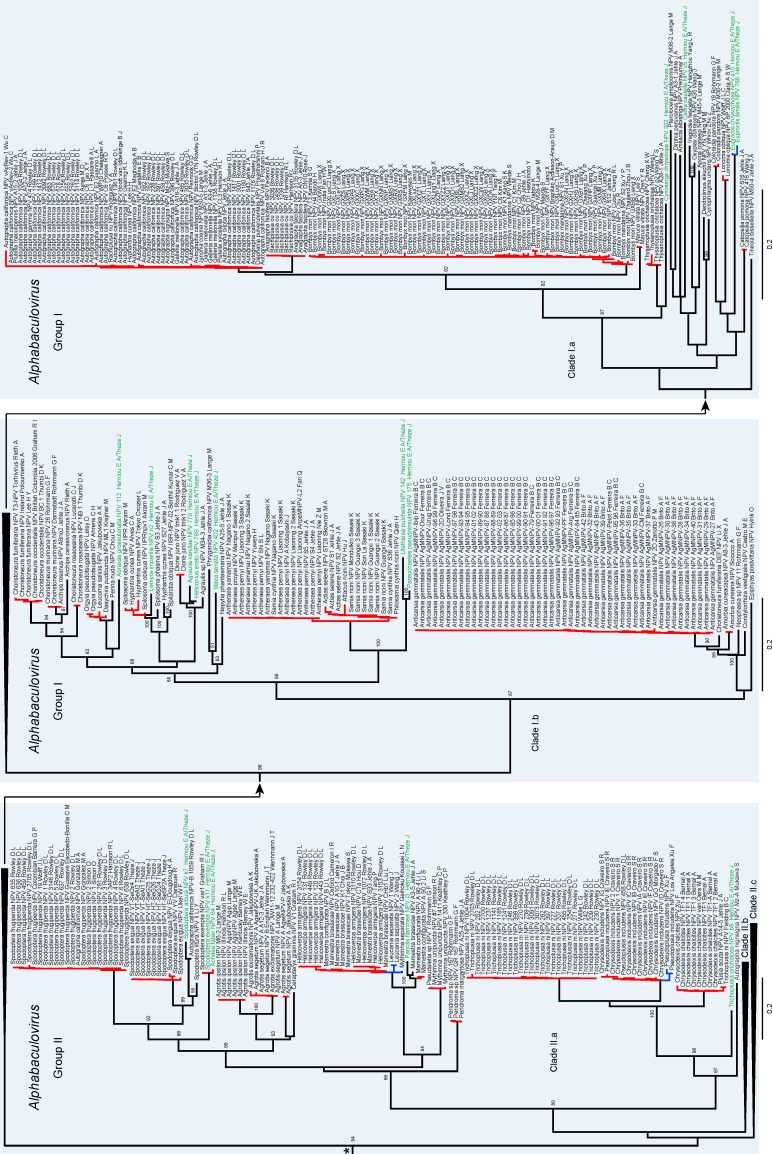
Baculovirus isolate phylogeny. The tree was obtained from a maximum likelihood inference analysis of the concatenated codon-based alignment (794 taxa) of four lepidopteran baculovirus core genes with the baculovirus core-genome phylogeny used as backbone tree (Figure S1). External clades colored in red correspond to clusters determined by both the mPTP (Figure S3) and SpDelim (Figure S4) species delimitation analysis and in blue the clusters not determined by SpDelim. The two star symbols point out the same node in the tree. Baculovirus isolate sequences generated in this study are highlighted in green. Statistical support for nodes in the tree corresponds to bootstraps (with 100 replicates).

Among the BV isolates, 217 have complete genomes and were used to reconstruct the highly supported BV core-genome phylogeny (Figure S1), inferred from the concatenated multiple amino acid alignment of the 37 BV core genes. The tree topology is in accordance with previous studies showing four BV genera infecting three distinct insect orders: the genera *Alphabaculovirus* and *Betabaculovirus* infect lepidopteran species, the genus *Gammabaculovirus* infects Hymenopteran species and the genus *Deltabaculovirus* infects dipteran species [14,15]. The topology obtained was used as backbone constraint tree to reconstruct the BV isolate phylogeny. The BV isolate phylogeny (Figure 1 and Figure S2) is quite well supported and is in accordance with previous studies showing similar topologies [7,14,17] with notably a separation of two major monophyletic subgroups of alphabaculoviruses (Group I, Group II). In addition, four minor monophyletic subgroups are outgroups to Group I and II. Within Group I we observed a clear division into two distinct monophyletic clades (I.a, I.b) and Group II is divided into three distinct monophyletic clades (II.a, II.b and II.c) (Figure 1 and Figure S2).

### 3.2. Species Delimitation

To infer macroevolutionary process, it is necessary to conduct analyses at the species level to avoid interference from intraspecific diversity. A species delimitation analysis was performed on the BV isolate tree (Figure 1; see also Figure S2) using the mPTP method and then compared to the commonly used genetic-distances-based method. The mPTP approach delimited 165 distinct putative BV species of which 70 putative BV species have been derived from clusters of two or more isolates and 95 putative species are singletons (based on a unique viral isolate) (Figure 1; see also Figures S2 and S3). The dataset includes 38 new BV species from our historical isolates. The genetic-distances-based method delimited 178 putative BV species (72 clusters and 106 singletons; Figure 1; see also Figures S2 and S4). The two approaches show 157 species in common, the differences in the genetic-distances-based method were mostly observed in alphabaculovirus species, showing high levels of sampling and genetic variability (Figure 1; see also Figure S2).

Within the 165 distinct species identified by mPTP, 116 putative species are alphabaculoviruses, 45 are betabaculoviruses, three are gammabaculoviruses and one is a deltabaculovirus (Table S3). The 10th report of the ICTV [52] currently only recognizes 68 species in the *Baculoviridae*: 40 in the genus *Alphabaculovirus*, 25 in the genus *Betabaculovirus*, two in the genus *Gammabaculovirus* and one in the genus *Deltabaculovirus* [14]. Our analysis leading to a reduced tree containing only putative BV species thus suggests 97 new putative BV species, including 76 alphabaculoviruses and 20 betabaculoviruses (Table S3).

### 3.3. Phylogenetic Conservatism and Host Shifts

Out of the 165 species of the genera *Alphabaculovirus* and *Betabaculovirus* identified by mPTP, 161 are associated with 187 Lepidoptera species from 24 different families. For all those lepidopteran hosts, we compiled a dataset with the taxonomy (superfamily, family and subfamily), the biogeographical distribution and the insect host plant range, from which we determined the host plant growth type (woody versus herbaceous) (Table S2). We performed phylogeny-trait correlation and ancestral state estimation analyses on the BV species phylogeny to measure whether those traits have evolved randomly or show phylogenetic conservatism. The different phylogeny-trait correlation tests show unequivocal significant associations between the taxonomy of insect hosts, the insect host plant growth type and the BV species phylogeny (AI, PS, PD, NT, NR, UniFrac and MC; *p* ≤ 0.01, Table 1). In contrast, no significant association was found between the insect host biogeography distribution and the BV species phylogeny, as shown by the high *p* values of certain tests (NR, UniFrac and MC; *p* > 0.01, Table 1).

**Figure 2:**
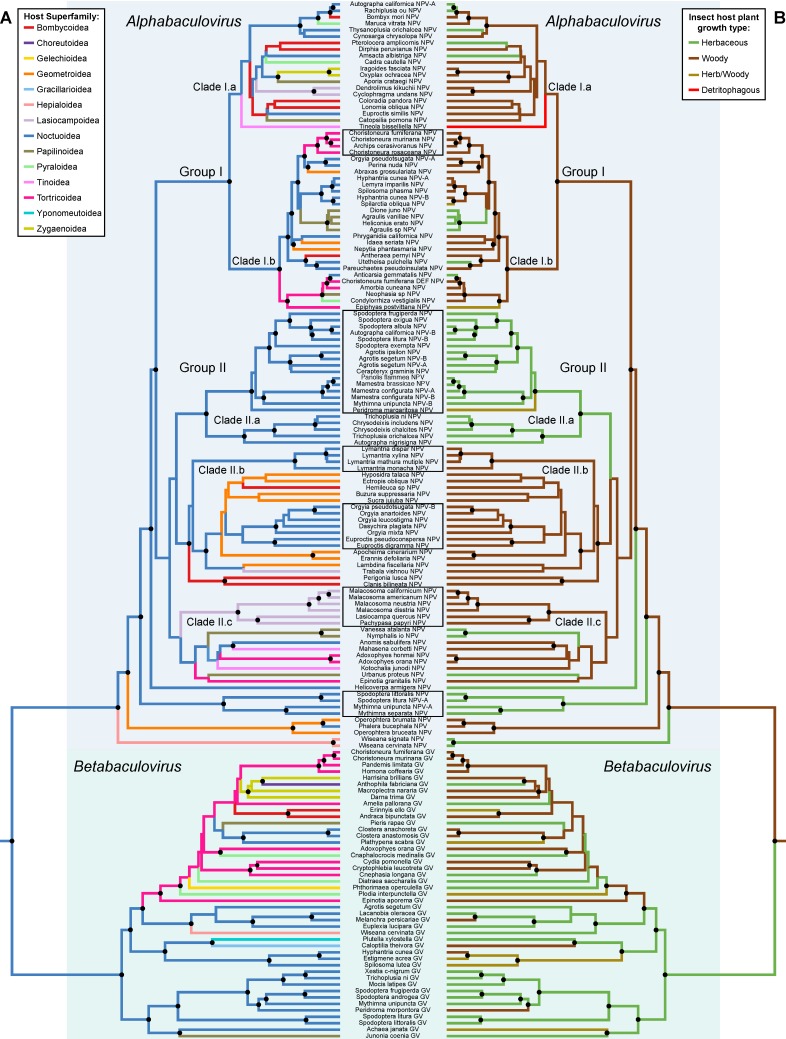
Phylogenetic optimizations on the lepidopteran baculovirus species tree. Optimizations were obtained by ancestral state estimation of (**A**) host Lepidoptera at superfamily level, and (**B**) insect host plant growth type traits on the ingroup of Lepidoptera infecting baculovirus. The term “Herb/woody” in the legend means that insect hosts feed on herbaceous plants as well as woody plants. Black circles close to phylogenetic nodes refer to posterior probabilities over 0.75. Baculovirus species denoted by black line rectangles correspond to clades that are significantly topologically congruent (*p* < 0.5) with lepidopteran host clades.

Ancestral state estimations on the BV species tree were performed on traits that showed significant associations such as host taxonomy and insect host plant growth. The estimations show highly significant levels of phylogenetic conservatism with the different host taxonomic levels and the insect host plant growth type (Table 1). For the host taxonomy trait, we illustrate only the insect host superfamily optimization, as it is the higher taxonomic level and has a reduced number of character states (Figure 2). The ancestral state estimation of the insect host superfamilies shows that closely related BV species tend to infect closely related lepidopterans (BVs cluster together according to the lepidopteran superfamilies) (Figure 2A). The host use optimization identifies owlet moths (Noctuoidea) as the most likely ancestral hosts of BVs (Figure 2A). Despite significant levels of phylogenetic conservatism several major host shifts across different lepidopteran superfamilies have occurred during BV evolution. Thus, in the *Betabaculovirus* genus we can see a host shift from Noctuoidea to the Tortricoidea superfamily and then colonization of several superfamilies: Papilionoidea, Zygaenoidea, Bombycoidea and a shift back to the Noctuoidea (Figure 2A). The *Alphabaculovirus* genus splits into two large lineages (Group I and II) and four small lineages (Figure 2A). Group I colonizes several Lepidoptera superfamilies, Clade I.a shows no host conservatism harboring a very diverse host range, whereas Clade I.b shows a medium level of host conservatism with a shifting from Noctuoidea to Tortricoidea and Papilionoidea. Group II shows a clear split between Clades II.a, II.b and II.c, with clades II.a and II.b showing a strong conservatism towards Noctuoidea with Clade II.a being specific to Noctuoidea with no apparent host shift. Both clades II.b and II.c show host shifts from Noctuoidea to Geometroidea and Lasiocampoidea and colonization of Bombycoideae, Tortricoidea, Tineoidea and Papilionoidea (Figure 2A).

Insect host plant growth type (Figure 2B), which can be considered as the local habitat of the virus, shows a strong phylogenetic conservatism (Table 1). Our analyses show woody plants as the most likely ancestral ecological niche of the first lepidopteran BVs. The *Betabaculovirus* genus is ancestrally associated with herbaceous plants before colonizing woody plants. The *Alphabaculovirus* genus is ancestrally associated with woody plants. Group I shows a strong association with woody plants with few shifts to herbaceous plants. The ancestors of group II likely fed on woody plants with colonization of herbaceous plants (Clade II.a). The three small alphabaculovirus clades, that are outgroups to Clade I and II, also show a shift to herbaceous plants. The most notable result in these analyses is the long-term associations with a particular insect host plant growth type. Strikingly, we noticed in the Group II of the *Alphabaculovirus* genus a split of virus infecting Noctuoidea, which seems to be the result of the colonization of herbaceous plant local habitat in Clade II.a (Figure 2).

### 3.4. Cophylogeny between Baculoviruses and Their Lepidopteran Hosts

The association between the topology of the BV species phylogeny and that of their lepidopteran hosts (at superfamily, family and subfamily levels) is not significantly different from random. In contrast, we found significant topological congruence (*p* < 0.5), meaning that the BV tree is the mirror image of lepidopteran host tree, for six BV clades at different taxonomic levels (see the six clades denoted by rectangles in Figure 2). The *Alphabaculovirus* genus gathers all the clades with significant topological congruence with host topologies. One clade infecting Tortricoidea hosts was detected in Group I, whereas four clades infecting Noctuoidea hosts were identified, and notably three in Group II. One clade infecting Lasiocampoidea was also detected in Group II.

## 4. Discussion

### 4.1. Reconstructing Phylogenies and Delineating Species

The *Baculoviridae* is by far the best described insect DNA virus family. For the last 50 years, BV use in biotechnology, either as expression vectors [1] or as microbial control agents for insect pests [53], has led to a vast production of molecular data, especially for the genera infecting the Lepidoptera, on which this study is focused. The first objective of this study was to reconstruct the most accurate and exhaustive BV isolate phylogeny in order to set a solid framework for species delimitation and macroevolutionary inference.

The backbone of the tree was built based on 217 whole BV genomes (Figure S1). A secondary ML analysis included an additional 577 isolates, from which we obtained our isolate tree (Figure 1 and Figure S2). We chose to cluster these viral isolates into species, which are the basis of biological classification, in order to study BV speciation at a macroevolutionary scale. BVs like most large DNA viruses are slow-evolving viruses with an approximate mutation rate of 10^−6^/10^−7^ (expressed as the number of substitutions per nucleotide per generation, defined as a cell infection in viruses) [54]. This mutation rate approaches those observed in bacteria and lower Eukaryotes. Consequently, we decided to use a phylogenetic-species-concept-based clustering approach, the mPTP model [37], to delimit BV species and to compare the results to the commonly used genetic-distances-based method (intra-species distance ≤0.015; up to 0.05 with complementary information [7]). Our species delimitation results were mostly consistent with the current taxonomy proposed by the ICTV [14] (Table S3). The only difference is that three species were found to each include two species classified by the ICTV (Table S3). Out of 165 BV species, we were able to characterize 97 that are not yet included in the ICTV report. The BV species phylogeny reflects current knowledge of BV diversity and phylogenetic relationships (Figure 1 and Figure 2). Furthermore, this study is the first to use a phylogenetic clustering approach for species delimitation in viruses, showing that its utility goes beyond vertebrates [55], invertebrates [56,57] and bacteria [12] taxa. Moreover, this approach for species delimitation fully respects the phylogenetic species concept and is less arbitrary than commonly used genetic distance approaches, which do not take into account differences in molecular evolutionary rates or sampling proportions and may thus vary depending on the biology of the lineage studied and on the gene used for phylogenetic reconstruction. Nevertheless, both species delimitation approaches gave relatively consistent results with an overlap of 157 species determined by both methods. The few differences in the genetic distances-based method were mostly observed in heterogeneous species clusters, with isolates infecting one or several hosts and showing high genetic diversity typically resulting in clusters with genetic distances between 0.015 and 0.050 [7]. The phylogenetic-based approach does not contradict the results of the genetic-distances-based approach but gives additional information to resolve the uncertainties of the genetic-distances-based approach.

### 4.2. Evolution of Host Use and Taxa Sampling

Most of the BV species identified in our study have isolates that infect only one lepidopteran host species. Strikingly, we generally found that closely related hosts belonging to the same genus were infected by different viral species (for example in the genera *Spodoptera*, *Lymantria*, or *Malacosoma*). This leads us to question the ecological reality and biological meaning of some isolates, such as *Trichoplusia ni* NPV and *Busseola fusca* NPV within the *Helicoverpa armigera* NPV clade (Figure 1). However, generalist viruses, capable of infecting different host species, belonging or not to the same genus, exist and the hosts they infect generally have overlapping ecological niches (same host plants). As an example, several isolates from different species of nymphalid butterflies that feed on nettles (*Urtica dioica*) form a single alphabaculovirus species *Vanessa atalanta* NPV.

As parasites replicating exclusively in host cells, BVs are involved in durable and intimate obligate interactions with their host, implicating long-term coevolution. The phylogenetic conservation results suggest an ancestral and frequent association with hosts of the Noctuoidea superfamily. This could possibly reflect the actual evolution of lepidopteran BVs and their current host range, as with ~42,000 out of ~157,000 described lepidopteran species the Noctuoidea is the most diversified superfamily (in comparison, the second most abundant superfamily is the Geometroidea with ~23,000 species) [31]. Yet, BV genetic data from public databases is clearly biased towards agro-economically important lepidopteran pests, characterized by large populations, important for sustaining BV populations. The addition of new BV samples from our collection tends to reduce the bias for pests, but BVs from pests still dominate our taxa sampling as these viruses have been isolated and resequenced many times (i.e., large clusters of *Cydia pomonella* GV or *Helicoverpa armigera* NPV; Figure 1) and as at least 62 out of 161 putative lepidopteran BV species are associated with pests (Figure 2A; Table S2). As numerous lepidopteran pests belong to the Noctuoidea superfamily, this probably increases the representation of Noctuoidea infecting BVs in our dataset and could have biased our phylogenetic conservatism results (77 out of 161 putative lepidopteran BV species analyzed attack Noctuoidea, Figure 2A). Only a diverse BV sampling, more representative of the lepidopteran diversity could confirm if Noctuoidea played a key role in BV evolution.

### 4.3. Cophylogeny and Host Shifts

Cophylogenetic analyses show no topological congruence between Lepidoptera and the BV species tree, but do show significant cophylogenetic signal between certain internal nodes of the BV species phylogeny and the associated insect host species nodes (Figure 2). This means that present host-use patterns in BVs result mainly from a pattern of host conservatism punctuated with occasional shifting among pre-existing insect lineages [58,59]. This coevolutionary association concurs with a general process of colonization by host tracking [60,61] as previously suggested at the macroevolutionary level of several insect DNA virus families [18]. However, this type of coevolutionary association is only observable for crown groups of viruses infecting hosts belonging to the same family and is lost in more basal nodes. Indeed, over a short timeframe BV speciation remains intimately linked to the speciation of their host, following the biogeography of their hosts and ultimately in certain lineages we observed BV phylogenies that are the mirror images of their host’s. Yet on a larger evolutionary scale, the insect-host coevolutionary relationship signal is confused, strongly suggesting that other factors act on BV evolution.

### 4.4. Ecological Specialization

The distinctive feature of the BV life cycle compared to most viruses is that they produce a transmission stage, which persists outside of the host and has the ability to resist environmental degradation. This feature is also found in other insect viruses such as entomopoxviruses and cypoviruses, which have similar life cycles and OBs. This highlights the importance and the persistence of this dissemination process in insect viruses [62]. As BVs only infect larvae and need to be ingested to initiate infection, they have an intimate association with the plants that their hosts feed on. In addition, there is an increasing body of evidence showing that host plant chemistry can moderate the BV infection process [25,28]. Thus, host plant characteristics could define the local biotopes of BVs. We therefore searched for conservatism with the type of plant used by insect hosts, distinguishing woody perennial (including shrubby and suffrutescent plants) and herbaceous plants. Results show ancestral associations to particular plant groups over several tens of million years (Figure 2B), suggesting a pattern of plant-use conservatism punctuated with sporadic shifts between plant growths. This underlines the predominant role of host plant association in BV evolution. As a consequence, BV diversification entangles patterns of host and local biotope conservatism.

Virus ecological niches are considered in general as defined only by their hosts, underestimating other factors and notably the influence of the environment. The BV niche combines a set of insect and insect host plant biotic conditions. Viruses consume the resources provided by their hosts in a host tracking fashion and consequently are influenced by their geographical distributions; hosts are therefore the set of biotic conditions where primary speciation takes places through adaptation to insect immunity and competition with other parasites. Host spectrum and hosts shifts are contained within local environments represented by a group of host plants. Host plant-use therefore defines a second ring of biotic environment that BVs experience. The association with particular types of plants persists over millions of years and drives BVs towards particular insect hosts. This is strikingly observable for the group II of the *Alphabaculovirus* genus, where BV infecting Noctuoidea species are split in two distinct clades, in one clade (clade II.a) Noctuoidea species are associated with herbaceous plants and in the other clade (clade II.b) they are associated with woody plants (Figure 2).

At the level of the evolutionary history of BVs, our study points out the specialization of BVs highlighted with topological congruence of certain BV-hosts associations. At the macroevolutionary timescale, BVs are specialized to a particular insect order and notably to three orders of holometabolous insects [15]. The BVs infecting Lepidoptera separate into two major groups with different types of OBs, the NPVs and the GVs. GVs (*Betabaculovirus*) seem to be ancestrally associated to herbaceous plants and to have colonized woody plants later. Strikingly, some GVs are associated with internal feeding hosts such as the codling moth, *Cydia pomonella*, the potato tuber moth, *Phthorimaea operculella* or the tea leaf-roller micromoth *Caloptilia theivora* and these types of hosts are not infected by NPVs. The morphology of GV OBs, which are much smaller than those of NPVs, may confer a more targeted dispersal strategy to increase the likelihood of reaching hosts concealed within the plant. Therefore, it is predicted that leaf-mining and/or stem-boring *Lepidoptera* should more likely be infected by GVs than by NPVs.

NPVs (*Alphabaculovirus*) appear to be ancestrally associated with woody plants and to have colonized herbaceous plants afterwards. NPVs belonging to group II are often very specialized to their hosts according to topological congruence with their associated hosts and the clear split of herbaceous (clade II.a) and woody (clade II.b) clades. By contrast, NPVs belonging to group I show a complex pattern of associations to several lepidopteran superfamilies on herbaceous and woody plants, suggesting more frequent host switches than those observed for group II or GVs. Remarkably, this group includes the well-known *Autographa californica* MNPV, which has a large host range, spanning different Lepidoptera species belonging to distantly related families [63] (Figure 1; Figure S2). This BV is a generalist virus, which is an uncommon trait in BVs. Generalism could favor host switching and explain loss of BV-host phylogenetic congruence in certain BV lineages, notably in the clade I.a of the *Alphabaculovirus* genus.

The host is by far the most important component of virus ecological niches, but not the only one. Most previous studies focused on microevolutionary patterns of diversification, resulting in host distribution outcomes to explain virus dynamics [64,65]. Here, we discuss the macroevolutionary patterns of an entire virus family based on a reconstruction, as exhaustive as possible, of its history. BVs have a peculiar life cycle where insect hosts and their associated plants are entangled. Viral transmission and fitness are increased with the typical production of OBs for environmental dissemination combined with the modification of host behavior, like the enhancement of climbing behavior [66,67]. Plants are therefore the vessel of viral transmission. Plants attacked by many lepidopteran species could support the evolution of generalist viruses. This in turn could promote host shifts with the subsequent specialization of particular viral lineages. Conversely plants attacked by few specialist Lepidoptera should foster the evolution of specialist viruses. Multitrophic interactions have thus shaped BV evolution as shown by the combined patterns of insect host and insect host plant conservatism, punctuated by occasional shifts among pre-existing insect lineages. However, the direct evolutionary interactions between plants and BVs remain undetermined.

## 5. Conclusions

Our current understanding of BV diversity and evolution has been fueled by decades of research on biological control and biotechnological applications. Here, we presented a complete evolutionary framework of the known BV diversity, highlighting the complex ecological interactions of these viruses with their hosts. Our study is the first to use a phylogenetic clustering approach for species delimitation in viruses characterizing many new BV species. Our analyses show that host shifts played a major role in the diversification of BVs. It also shows that the colonization of a new ecological niche (herbaceous plants) lead to the radiation of some BV lineages. BV species richness results from millions of years of evolutionary interactions between the host plant ecology and chemistry and the physiology and ecology of both BVs and their associated insect hosts. This is conveyed at the genome level by an average of 150 genes, of which only 37 are core genes shared by all BVs, and a further 26 shared by all lepidopteran BVs [32]. The remaining so-called accessory genes may encode specific adaptive proteins [68] that could prove useful to improve expression vectors or agricultural biocontrol applications. Indeed, within the catalogue of BV genes, there are still many genes of unknown functions, including some that could enhance viral replication and protein production capacity, or that could modulate virulence or host specificity. It is thus quite probable that by its diversity the family *Baculoviridae* potentially still hides a treasure trove of genes and molecules that could lead to innovative biotechnology.

## Figures and Tables

**Table 1:** Phylogeny-trait correlations estimated under different statistical methods.

Traits	AI ^1^	PS ^1^	PD ^1^	NT ^1^	NR ^1^	UniFrac ^1^	MC ^2^
Host superfamily	<0.001	<0.001	<0.001	<0.001	<0.001	<0.001	<0.001/21
Host family	<0.001	<0.001	<0.001	<0.001	<0.001	<0.001	<0.001/21
Host subfamily	<0.001	<0.001	<0.001	<0.001	<0.001	<0.001	<0.001/7
Host biogeography distribution (ecozone)	<0.001	<0.001	<0.001	<0.001	0.58	0.029	0.019/4
Insect host plant growth type (Herb/Woody)	<0.001	<0.001	<0.001	<0.001	<0.001	<0.001	<0.001/22

^1^*p* value of AI: association index; PS: parsimony score; PD: phylogenetic diversity index; NT: nearest taxa index; NR: net relatedness index; UniFrac: unique fraction index; MC: maximum monophyletic clade size probability; ^2^ *p* value/size of MC: Maximum monophyletic clade size probability and size (number of taxa) of the largest monophyletic clade associated to one character of the trait.

## Supplementary Materials

The following are available online at http://www.mdpi.com/1999-4915/10/7/366/s1, Figure S1: Baculovirus core-genome phylogeny, Figure S2: Baculovirus isolate phylogeny (one panel Figure 1), Figure S3: Baculovirus isolate phylogeny including mPTP species delimitation results, Figure S4: Baculovirus isolate phylogeny including SpDelim species delimitation results, Table S1: Baculovirus isolate sequence database, Table S2: Host ecology database, Table S3: Baculovirus species delimitation.
