# Supplementary material for: Biodiversity, Evolution and Ecological Specialization of Baculoviruses: A Treasure Trove for Future Applied Research
Source: Viruses. 2018 Jul 11;10(7):366. doi: 10.3390/v10070366 (PMC6071083; doi:10.3390/v10070366)
Supplement: Supplementary file 1 [file viruses-10-00366-s001.pdf]

Supplementary Material

Biodiversity, evolution and ecological specialization of baculoviruses: a treasure trove for future applied research

Julien Thézé[1,2]; Carlos Lopez-Vaamonde[1,3]; Jenny S. Cory[4]; Elisabeth A. Herniou[1]

[1] Institut de Recherche sur la Biologie de l'Insecte, UMR 7261, CNRS - Université de Tours, 37200 Tours, France; elisabeth.herniou@univ-tours.fr

[2] Department of Zoology, University of Oxford, South Parks Road, Oxford, OX1 3SY, UK; julien.theze@zoo.ox.ac.uk

[3] INRA, UR633 Zoologie Forestière, 45075 Orléans, France; carlos.lopezvaamonde@inra.fr

[4] Department of Biological Sciences, Simon Fraser University, Burnaby, V5A 1S6, British Columbia, Canada; jennifer_cory@sfu.ca

* Correspondence: elisabeth.herniou@univ-tours.fr; Tel.: +33-247-367381

Supplementary figure legends

Figure S1. Baculovirus core-genome phylogeny. The tree was obtained from maximum likelihood inference analysis of the concatenated amino acid alignment of the 37 baculovirus core genes. Statistical support for nodes in the ML tree was assessed using a bootstrap approach (with 100 replicates).

Figure S2. Baculovirus isolate phylogeny (one panel Figure 1). The tree was obtained from a maximum likelihood inference analysis of the concatenated codon-based alignment (794 taxa) of four lepidopteran baculovirus core genes with the baculovirus core-genome phylogeny used as backbone tree. External clades coloured in red correspond to clusters determined by both the mPTP and SpDelim species delimitation analysis and in blue the clusters not determined by SpDelim. Baculovirus isolates generated in this study are highlighted in green. Statistical support for nodes in the tree corresponds to bootstraps (with 100 replicates).

Figure S3. Baculovirus isolate phylogeny including mPTP species delimitation results. The tree was obtained from a maximum likelihood inference analysis of the concatenated codon-based alignment (794 taxa) of four lepidopteran baculovirus core genes with the baculovirus core-genome phylogeny used as backbone tree. External clades coloured in red correspond to clusters determined by the mPTP species delimitation analysis.

Figure S4. Baculovirus isolate phylogeny including SpDelim species delimitation results. The tree was obtained from a maximum likelihood inference analysis of the concatenated codon-based alignment (794 taxa) of four lepidopteran baculovirus core genes with the baculovirus core-genome phylogeny used as backbone tree. External clades coloured with various colours correspond to clusters determined by the SpDelim species delimitation analysis.

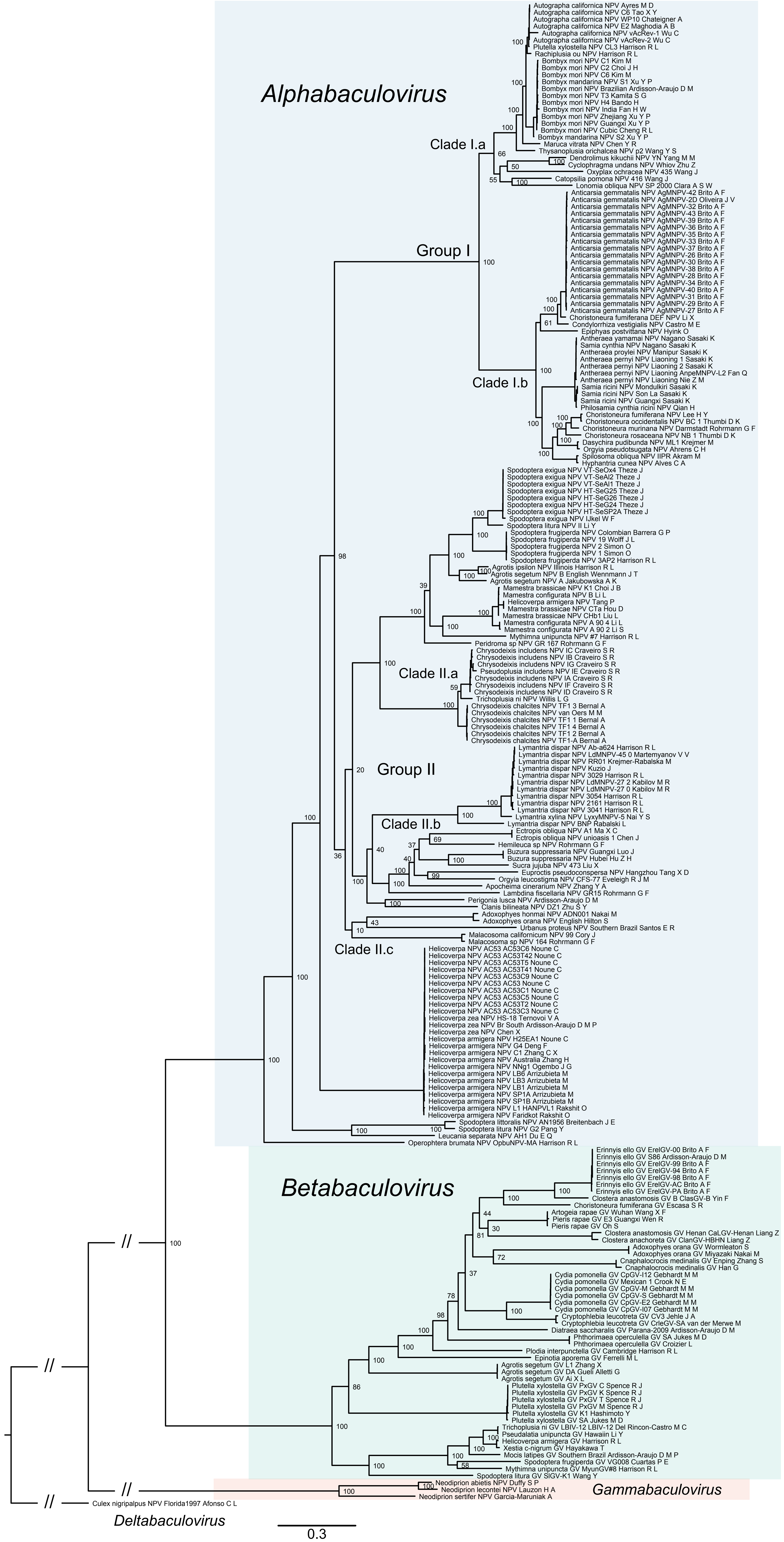

Alphabaculovirus
Autographa californica NPV Ayres M D
Autographa californica NPV C6 Tao X Y
Autographa californica NPV WP10 Chateigner A
Autographa californica NPV E2 Maghodia A B
Autographa californica NPV vAcRev-1 Wu C
Autographa californica NPV vAcRev-2 Wu C
Plutella xylostella NPV CL3 Harrison R L
Rachiplusia ou NPV Harrison R L
Bombyx mori NPV C1 Kim M
Bombyx mori NPV C2 Choi J H
Bombyx mori NPV C6 Kim M
Bombyx mandarina NPV S1 Xu Y P
Bombyx mori NPV Brazilian Ardisson-Araujo D M
Bombyx mori NPV T3 Kamita S G
Bombyx mori NPV H4 Bando H
Bombyx mori NPV India Fan H W
Bombyx mori NPV Zhejiang Xu Y P
Bombyx mori NPV Guangxi Xu Y P
Bombyx mori NPV Cubic Cheng R L
Bombyx mandarina NPV S2 Xu Y P
Maruca vitrata NPV Chen Y R
Thysanoplusia orichalcea NPV p2 Wang Y S
Dendrolimus kikuchii NPV YN Yang M M
Cyclophragma undans NPV Whiov Zhu Z
Oxyplax ochracea NPV 435 Wang J
Catopsilia pomona NPV 416 Wang J
Lonomia obliqua NPV SP 2000 Clara A S W
Clade I.a
Group I
Anticarsia gemmatalis NPV AgMNPV-42 Brito A F
Anticarsia gemmatalis NPV AgMNPV-2D Oliveira J V
Anticarsia gemmatalis NPV AgMNPV-32 Brito A F
Anticarsia gemmatalis NPV AgMNPV-43 Brito A F
Anticarsia gemmatalis NPV AgMNPV-39 Brito A F
Anticarsia gemmatalis NPV AgMNPV-36 Brito A F
Anticarsia gemmatalis NPV AgMNPV-35 Brito A F
Anticarsia gemmatalis NPV AgMNPV-33 Brito A F
Anticarsia gemmatalis NPV AgMNPV-37 Brito A F
Anticarsia gemmatalis NPV AgMNPV-26 Brito A F
Anticarsia gemmatalis NPV AgMNPV-30 Brito A F
Anticarsia gemmatalis NPV AgMNPV-38 Brito A F
Anticarsia gemmatalis NPV AgMNPV-28 Brito A F
Anticarsia gemmatalis NPV AgMNPV-34 Brito A F
Anticarsia gemmatalis NPV AgMNPV-40 Brito A F
Anticarsia gemmatalis NPV AgMNPV-31 Brito A F
Anticarsia gemmatalis NPV AgMNPV-29 Brito A F
Anticarsia gemmatalis NPV AgMNPV-27 Brito A F
Choristoneura fumiferana DEF NPV Li X
Condylorrhiza vestigialis NPV Castro M E
Epiphyas postvittana NPV Hyink O
Antheraea yamamai NPV Nagano Sasaki K
Samia cynthia NPV Nagano Sasaki K
Antheraea proylei NPV Manipur Sasaki K
Antheraea pernyi NPV Liaoning 1 Sasaki K
Antheraea pernyi NPV Liaoning 2 Sasaki K
Antheraea pernyi NPV Liaoning AnpeMNPV-L2 Fan Q
Antheraea pernyi NPV Liaoning Nie Z M
Samia ricini NPV Mondulkiri Sasaki K
Samia ricini NPV Son La Sasaki K
Samia ricini NPV Guangxi Sasaki K
Philosamia cynthia ricini NPV Qian H
Choristoneura fumiferana NPV Lee H Y
Choristoneura occidentalis NPV BC 1 Thumbi D K
Choristoneura murinana NPV Darmstadt Rohrmann G F
Choristoneura rosaceana NPV NB 1 Thumbi D K
Dasychira pudibunda NPV ML1 Krejmer M
Orgyia pseudotsugata NPV Ahrens C H
Spilosoma obliqua NPV IIPR Akram M
Hyphantria cunea NPV Alves C A
Clade I.b
Spodoptera exigua NPV VT-SeOx4 Theze J
Spodoptera exigua NPV VT-SeAl2 Theze J
Spodoptera exigua NPV VT-SeAl1 Theze J
Spodoptera exigua NPV HT-SeG25 Theze J
Spodoptera exigua NPV HT-SeG26 Theze J
Spodoptera exigua NPV HT-SeG24 Theze J
Spodoptera exigua NPV HT-SeSP2A Theze J
Spodoptera exigua NPV IJkel W F
Spodoptera litura NPV II Li Y
Spodoptera frugiperda NPV Colombian Barrera G P
Spodoptera frugiperda NPV 19 Wolff J L
Spodoptera frugiperda NPV 2 Simon O
Spodoptera frugiperda NPV 1 Simon O
Spodoptera frugiperda NPV 3AP2 Harrison R L
Agrotis ipsilon NPV Illinois Harrison R L
Agrotis segetum NPV B English Wennmann J T
Agrotis segetum NPV A Jakubowska A K
Mamestra brassicae NPV K1 Choi J B
Mamestra configurata NPV B Li L
Helicoverpa armigera NPV Tang P
Mamestra configurata NPV CTa Hou D
Mamestra brassicae NPV CHb1 Liu L
Mamestra configurata NPV A 90 4 Li L
Mamestra configurata NPV A 90 2 Li S
Mythimna unipuncta NPV #7 Harrison R L
Peridroma sp NPV GR 167 Rohrmann G F
Chrysodeixis includens NPV IC Craveiro S R
Chrysodeixis includens NPV IB Craveiro S R
Chrysodeixis includens NPV IG Craveiro S R
Pseudoplusia includens NPV IE Craveiro S R
Chrysodeixis includens NPV IA Craveiro S R
Chrysodeixis includens NPV IF Craveiro S R
Chrysodeixis includens NPV ID Craveiro S R
Trichoplusia ni NPV Willis L G
Chrysodeixis chalcites NPV TF1 3 Bernal A
Chrysodeixis chalcites NPV van Oers M M
Chrysodeixis chalcites NPV TF1 1 Bernal A
Chrysodeixis chalcites NPV TF1 4 Bernal A
Chrysodeixis chalcites NPV TF1 2 Bernal A
Chrysodeixis chalcites NPV TF1-A Bernal A
Clade II.a
Group II
Lymantria dispar NPV Ab-a624 Harrison R L
Lymantria dispar NPV LdMNPV-45 0 Martemyanov V V
Lymantria dispar NPV RR01 Krejmer-Rabalska M
Lymantria dispar NPV Kuzio J
Lymantria dispar NPV 3029 Harrison R L
Lymantria dispar NPV LdMNPV-27 2 Kabilov M R
Lymantria dispar NPV LdMNPV-27 0 Kabilov M R
Lymantria dispar NPV 3054 Harrison R L
Lymantria dispar NPV 2161 Harrison R L
Lymantria dispar NPV 3041 Harrison R L
Lymantria xylina NPV LyxyMNPV-5 Nai Y S
Lymantria dispar NPV BNP Rabalski L
Ectropis obliqua NPV A1 Ma X C
Ectropis obliqua NPV unioasis 1 Chen J
Hemileuca sp NPV Rohrmann G F
Buzura suppressaria NPV Guangxi Luo J
Buzura suppressaria NPV Hubei Hu Z H
Sucra jujuba NPV 473 Liu X
Euproctis pseudoconspersa NPV Hangzhou Tang X D
Orgyia leucostigma NPV CFS-77 Eveleigh R J M
Apocheima cinerarium NPV Zhang Y A
Lambdina fiscellaria NPV GR15 Rohrmann G F
Perigonia lusca NPV Ardisson-Araujo D M
Clanis bilineata NPV DZ1 Zhu S Y
Clade II.b
Adoxophyes honmai NPV ADN001 Nakai M
Adoxophyes orana NPV English Hilton S
Urbanus proteus NPV Southern Brazil Santos E R
Malacosoma californicum NPV 99 Cory J
Malacosoma sp NPV 164 Rohrmann G F
Clade II.c
Helicoverpa NPV AC53 AC53C6 Noune C
Helicoverpa NPV AC53 AC53T42 Noune C
Helicoverpa NPV AC53 AC53T5 Noune C
Helicoverpa NPV AC53 AC53T41 Noune C
Helicoverpa NPV AC53 AC53C9 Noune C
Helicoverpa NPV AC53 AC53 Noune C
Helicoverpa NPV AC53 AC53C1 Noune C
Helicoverpa NPV AC53 AC53C5 Noune C
Helicoverpa NPV AC53 AC53T2 Noune C
Helicoverpa NPV AC53 AC53C3 Noune C
Helicoverpa zea NPV HS-18 Ternovoi V A
Helicoverpa zea NPV Br South Ardisson-Araujo D M P
Helicoverpa zea NPV Chen X
Helicoverpa armigera NPV H25EA1 Noune C
Helicoverpa armigera NPV G4 Deng F
Helicoverpa armigera NPV C1 Zhang C X
Helicoverpa armigera NPV Australia Zhang H
Helicoverpa armigera NPV NNg1 Ogembo J G
Helicoverpa armigera NPV LB6 Arrizubieta M
Helicoverpa armigera NPV LB3 Arrizubieta M
Helicoverpa armigera NPV LB1 Arrizubieta M
Helicoverpa armigera NPV SP1A Arrizubieta M
Helicoverpa armigera NPV SP1B Arrizubieta M
Helicoverpa armigera NPV L1 HANPVL1 Rakshit O
Helicoverpa armigera NPV Faridkot Rakshit O
Spodoptera littoralis NPV AN1956 Breitenbach J E
Spodoptera litura NPV G2 Pang Y
Leucania separata NPV AH1 Du E Q
Operophtera brumata NPV OpbuNPV-MA Harrison R L
Betabaculovirus
Erinnyis ello GV ErelGV-00 Brito A F
Erinnyis ello GV S86 Ardisson-Araujo D M
Erinnyis ello GV ErelGV-99 Brito A F
Erinnyis ello GV ErelGV-94 Brito A F
Erinnyis ello GV ErelGV-98 Brito A F
Erinnyis ello GV ErelGV-AC Brito A F
Erinnyis ello GV ErelGV-PA Brito A F
Clostera anastomosis GV B ClasGV-B Yin F
Choristoneura fumiferana GV Escasa S R
Artogeia rapae GV Wuhan Wang X F
Pieris rapae GV E3 Guangxi Wen R
Pieris rapae GV Oh S
Clostera anastomosis GV Henan CaLGV-Henan Liang Z
Clostera anachoreta GV ClanGV-HBHN Liang Z
Adoxophyes orana GV Wormleaton S
Adoxophyes orana GV Miyazaki Nakai M
Cnaphalocrocis medinalis GV Enping Zhang S
Cnaphalocrocis medinalis GV Han G
Cydia pomonella GV CpGV-I12 Gebhardt M M
Cydia pomonella GV Mexican 1 Crook N E
Cydia pomonella GV CpGV-M Gebhardt M M
Cydia pomonella GV CpGV-S Gebhardt M M
Cydia pomonella GV CpGV-E2 Gebhardt M M
Cydia pomonella GV CpGV-I07 Gebhardt M M
Cryptophlebia leucotreta GV CV3 Jehle J A
Cryptophlebia leucotreta GV CrleGV-SA van der Merwe M
Diatraea saccharalis GV Parana-2009 Ardisson-Araujo D M
Phthorimaea operculella GV SA Jukes M D
Phthorimaea operculella GV Croizier L
Plodia interpunctella GV Cambridge Harrison R L
Epinotia aporema GV Ferrelli M L
Agrotis segetum GV L1 Zhang X
Agrotis segetum GV DA Gueli Alletti G
Agrotis segetum GV Ai X L
Plutella xylostella GV PxGV C Spence R J
Plutella xylostella GV PxGV K Spence R J
Plutella xylostella GV PxGV T Spence R J
Plutella xylostella GV PxGV M Spence R J
Plutella xylostella GV K1 Hashimoto Y
Plutella xylostella GV SA Jukes M D
Trichoplusia ni GV LBIV-12 LBIV-12 Del Rincon-Castro M C
Pseudaletia unipuncta GV Hawaiin Li Y
Helicoverpa armigera GV Harrison R L
Xestia c-nigrum GV Hayakawa T
Mocis latipes GV Southern Brazil Ardisson-Araujo D M P
Spodoptera frugiperda GV VG008 Cuartas P E
Mythimna unipuncta GV MyunGV#8 Harrison R L
Spodoptera litura GV SlGV-K1 Wang Y
Neodiprion abietis NPV Duffy S P
Neodiprion lecontei NPV Lauzon H A
Neodiprion sertifer NPV Garcia-Maruniak A
Gammabaculovirus
Culex nigripalpus NPV Florida1997 Afonso C L
Deltabaculovirus
0.3

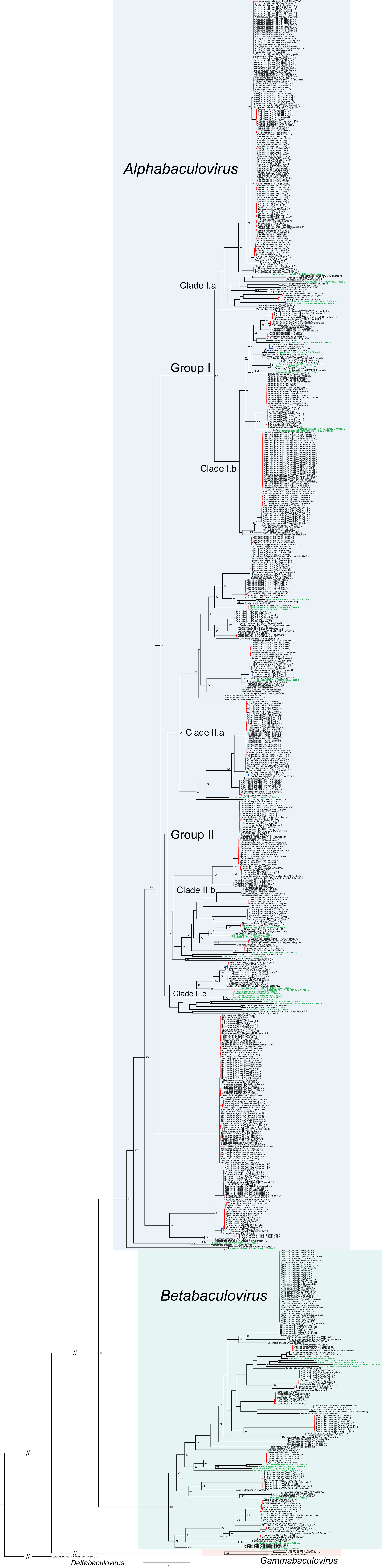

Alphabaculovirus
Clade I.a
Group I
Clade I.b
Clade II.a
Group II
Clade II.b
Clade II.c
Betabaculovirus
Deltabaculovirus
Gammabaculovirus
0.3

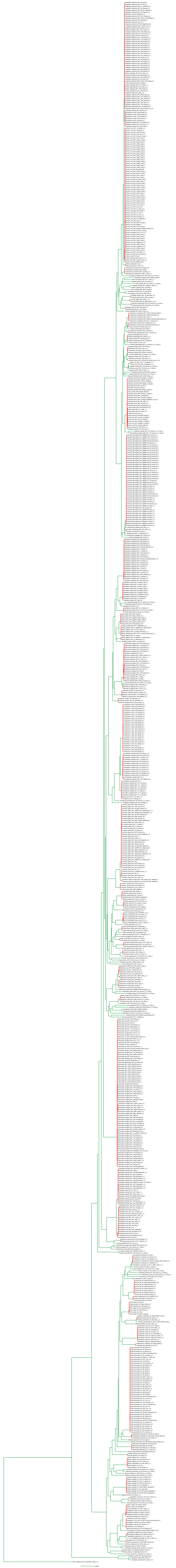

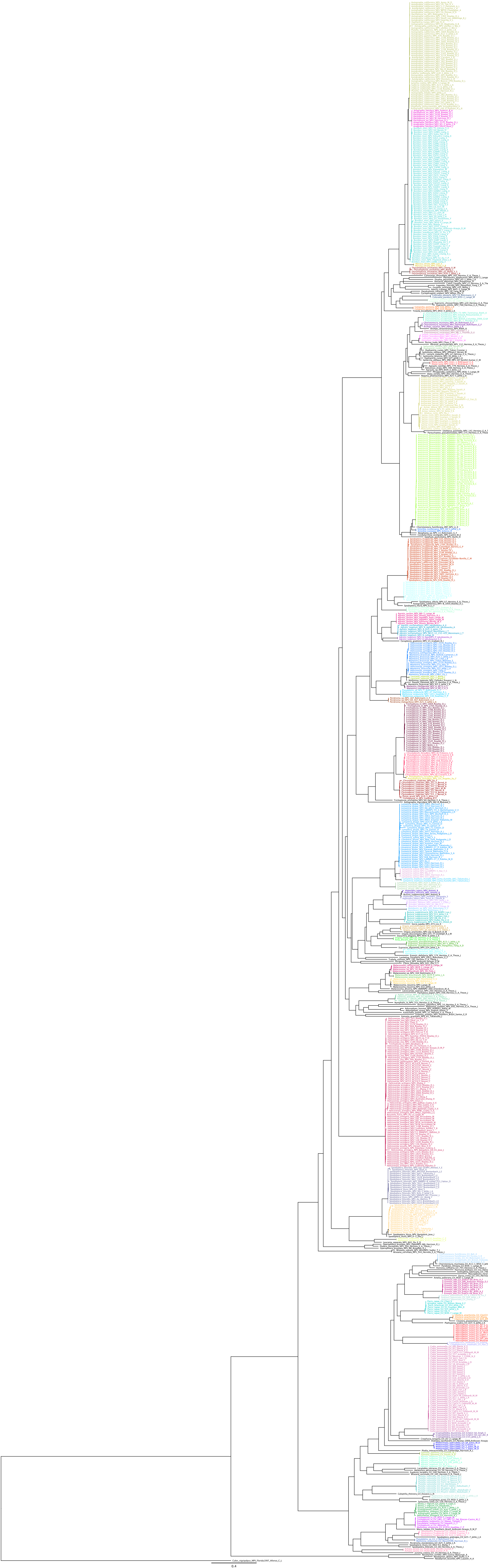
0.4

Table S1. Baculovirus isolate sequence database

Virus name	Isolate	Strain	Author	Complete genome	lef-8 accession number	lef-9 accession number	pif-2 accession number	polh accession number
Abraxas grossulariata NPV	112	-	Herniou E A/Thézé J.	No	AY449781.1	-	AY449761.1	MH454186
Achaea faber NPV	64	-	Herniou E A/Thézé J.	No	MH454109	MH454147	MH458171	MH454187
Achaea janata GV	-	Hyderabad	Kumar P N.	No	-	-	-	JX426115.1
Achaea janata GV	-	Hyderabad	Naveen Kumar P.	No	-	JX987080.1	-	JX392405.1
Actias selene NPV	-	S1	Jehle J A.	No	AY706527.1	AY706592.1	-	AY706680.1
Actias selene NPV	-	S2	Jehle J A.	No	AY706525.1	AY706590.1	-	AY706678.1
Actias selene NPV	-	ST28	Skowron M A.	No	-	KR057235.1	-	KR057236.1
Adoxophyes honmai NPV	ADN001	-	Nakai M.	Yes	AP006270.1	AP006270.1	AP006270.1	AP006270.1
Adoxophyes orana GV	E1	-	Wormleaton S L.	No	-	-	-	AF337646.1
Adoxophyes orana GV	Miyazaki	-	Nakai M.	Yes	KM226332.1	KM226332.1	KM226332.1	KM226332.1
Adoxophyes orana GV	-	A2-3	Lange M.	No	AY519259.1	AY519260.1	-	AY519258.1
Adoxophyes orana GV	-	A6-5	Jehle J A.	No	AY706529.1	AY706594.1	-	AY706657.1
Adoxophyes orana GV	-	Capex1-L2	Kundu J K.	No	-	-	-	EU107362.1
Adoxophyes orana GV	-	Capex-L9	Kundu J K.	No	-	-	-	EU107363.1
Adoxophyes orana GV	-	H	Kundu J K.	No	-	-	-	EU107360.1
Adoxophyes orana GV	-	S45	Jehle J A.	No	AY706530.1	AY706595.1	-	AY706658.1
Adoxophyes orana GV	-	-	Wormleaton S.	Yes	AF547984.1	AF547984.1	AF547984.1	AF547984.1
Adoxophyes orana NPV	-	English	Hilton S.	Yes	EU591746.1	EU591746.1	EU591746.1	EU591746.1
Aglais urticae NPV	a7	-	Herniou E A/Thézé J.	No	MH454110	MH454148	MH458172	MH454188
Agraulis sp NPV	-	M34-3	Jehle J A.	No	AY706532.1	AY706597.1	-	AY706682.1
Agraulis vanillae NPV	779	-	Herniou E A/Thézé J.	No	MH454111	MH454149	-	MH454189
Agrotis exclamationis GV	-	S46	Jehle J A.	No	AY706531.1	AY706596.1	-	AY706659.1
Agrotis exclamationis NPV	-	JW11-12_232-422	Wennmann J T.	No	-	-	-	KJ995661.1
Agrotis exclamationis NPV	-	-	Jakubowska A K.	No	-	-	-	GQ475265.1
Agrotis ipsilon MNPV	Illinois	-	Harrison R L.	Yes	EU839994.1	EU839994.1	EU839994.1	EU839994.1
Agrotis ipsilon MNPV	-	AgipNPV_Agip	Lange M.	No	-	-	-	AY136483.1
Agrotis ipsilon MNPV	-	AgipNPV_Agse	Lange M.	No	-	-	-	AY136484.1
Agrotis ipsilon MNPV	-	Illinois	Barney W E.	No	-	-	-	DQ014543.1
Agrotis ipsilon MNPV	-	Kentucky	Barney W E.	No	-	-	-	DQ014542.1
Agrotis ipsilon MNPV	-	M6-2	Lange M.	No	AY519205.1	AY519206.1	-	AY519204.1
Agrotis segetum GV	DA	-	Gueli Alletti G.	Yes	KR584663.1	KR584663.1	KR584663.1	KR584663.1
Agrotis segetum GV	L1	-	Zhang X.	Yes	KC994902.1	KC994902.1	KC994902.1	KC994902.1
Agrotis segetum GV	-	A17-5	Jehle J A.	No	AY706533.1	AY706598.1	-	AY706660.1
Agrotis segetum GV	-	S47	Jehle J A.	No	AY706534.1	AY706599.1	-	AY706661.1
Agrotis segetum GV	-	-	Ai X L.	Yes	AY522332.4	AY522332.4	AY522332.4	AY522332.4
Agrotis segetum NPV A	-	A12-3	Jehle J A.	No	AY706535.1	AY706600.1	-	AY706683.1
Agrotis segetum NPV A	-	AgseNPV-P	Jakubowska A.	No	AY971676.2	-	AY971677.1	AY971675.1
Agrotis segetum NPV A	-	AgseNPV-UK	Jakubowska A.	No	AY971678.1	-	AY971679.1	-
Agrotis segetum NPV A	-	-	Jakubowska A K.	Yes	DQ123841.1	DQ123841.1	DQ123841.1	DQ123841.1
Agrotis segetum NPV A	-	-	Lange M.	No	-	-	-	AY136482.1
Agrotis segetum NPV B	-	English	Wennmann J T.	Yes	KM102981.1	KM102981.1	KM102981.1	KM102981.1
Amelia pallorana GV	-	M30-1	Lange M.	No	AY519208.1	AY519209.1	-	AY519207.1
Pareuchaetes pseudoinsulata NPV	175	-	Herniou E A/Thézé J.	No	MH454112	MH454150	MH458173	MH454190
Amorbia cuneacapsa NPV	-	A8-3	Jehle J A.	No	AY706537.1	AY706602.1	-	AY706685.1
Amorbia cuneana NPV	-	-	Sciocco A.	No	EU698889.1	EU698890.1	-	EU698891.1
Amsacta albistriga NPV	-	-	Premkumar A.	No	-	-	-	AF118850.1
Anagrapha falcifera MNPV	-	3135	Rowley D L.	No	JN674780.1	JN674776.1	-	JN674772.1
Anagrapha falcifera MNPV	-	A5-3	Jehle J A.	No	AY706539.1	AY706604.1	-	AY706686.1
Anagrapha falcifera MNPV	-	DN10	Rose J.	No	KC800710.1	KC800711.1	-	KC800712.1
Anagrapha falcifera MNPV	-	-	Federici B A.	No	-	-	-	U64896.1
Andraca bipunctata GV	-	S48	Jehle J A.	No	AY706538.1	AY706603.1	-	AY706662.1
Andraca bipunctata GV	-	-	Zhang D.	No	-	-	-	AY518318.1
Anomis sabulifera NPV	14	-	Herniou E A/Thézé J.	No	MH454113	MH454151	-	MH454191
Antheraea pernyi NPV	A	-	Kobayashi J.	No	-	-	-	AB062454.1
Antheraea pernyi NPV	Liaoning	1	Sasaki K.	Yes	LC194889.1	LC194889.1	LC194889.1	LC194889.1
Antheraea pernyi NPV	Liaoning	2	Sasaki K.	Yes	LC375540.1	LC375540.1	LC375540.1	LC194890.1
Antheraea pernyi NPV	Liaoning	3	Sasaki K.	No	-	-	-	LC375540.1
Antheraea pernyi NPV	Liaoning	AnpeMNPV-L2	Fan Q.	Yes	EF207986.1	EF207986.1	EF207986.1	EF207986.1
Antheraea pernyi NPV	Liaoning	-	Nie Z M.	Yes	DQ486030.3	DQ486030.3	DQ486030.3	DQ486030.3
Antheraea pernyi NPV	-	S4	Jehle J A.	No	DQ231349.1	DQ231348.1	-	DQ231347.1
Antheraea pernyi NPV	-	S5	Jehle J A.	No	AY706540.1	AY706605.1	-	AY706687.1
Antheraea pernyi NPV	-	-	Shi S L.	No	-	-	-	AY685706.1
Antheraea pernyi NPV	-	-	Yuwen H.	No	-	-	-	M57429.1
Antheraea proylei NPV	Manipur	-	Sasaki K.	Yes	LC375539.1	LC375539.1	LC375539.1	LC375539.1
Antheraea yamamai NPV	Nagano	1	Sasaki K.	No	-	-	-	LC194891.1
Antheraea yamamai NPV	Nagano	2	Sasaki K.	No	-	-	-	LC375537.1
Antheraea yamamai NPV	Nagano	-	Sasaki K.	Yes	LC375537.1	LC375537.1	LC375537.1	-
Anthophila fabriciana GV	790	-	Herniou E A/Thézé J.	No	MH454114	MH454152	MH458174	MH454192
Anticarsia gemmatalis MNPV	-	AgMNPV-00_01	Ferreira B C.	No	-	-	KF765835.1	-
Anticarsia gemmatalis MNPV	-	AgMNPV-01_02	Ferreira B C.	No	-	-	KF765836.1	-
Anticarsia gemmatalis MNPV	-	AgMNPV-02_03	Ferreira B C.	No	-	-	KF765837.1	-
Anticarsia gemmatalis MNPV	-	AgMNPV-26	Brito A F.	Yes	KR815455.1	KR815455.1	KR815455.1	KR815455.1
Anticarsia gemmatalis MNPV	-	AgMNPV-27	Brito A F.	Yes	KR815456.1	KR815456.1	KR815456.1	KR815456.1
Anticarsia gemmatalis MNPV	-	AgMNPV-28	Brito A F.	Yes	KR815457.1	KR815457.1	KR815457.1	KR815457.1
Anticarsia gemmatalis MNPV	-	AgMNPV-29	Brito A F.	Yes	KR815458.1	KR815458.1	KR815458.1	KR815458.1
Anticarsia gemmatalis MNPV	-	AgMNPV-2D	Oliveira J V.	Yes	DQ813662.2	DQ813662.2	DQ813662.2	DQ813662.2
Anticarsia gemmatalis MNPV	-	AgMNPV-30	Brito A F.	Yes	KR815459.1	KR815459.1	KR815459.1	KR815459.1
Anticarsia gemmatalis MNPV	-	AgMNPV-31	Brito A F.	Yes	KR815460.1	KR815460.1	KR815460.1	KR815460.1
Anticarsia gemmatalis MNPV	-	AgMNPV-32	Brito A F.	Yes	KR815461.1	KR815461.1	KR815461.1	KR815461.1
Anticarsia gemmatalis MNPV	-	AgMNPV-33	Brito A F.	Yes	KR815462.1	KR815462.1	KR815462.1	KR815462.1
Anticarsia gemmatalis MNPV	-	AgMNPV-34	Brito A F.	Yes	KR815463.1	KR815463.1	KR815463.1	KR815463.1
Anticarsia gemmatalis MNPV	-	AgMNPV-35	Brito A F.	Yes	KR815464.1	KR815464.1	KR815464.1	KR815464.1
Anticarsia gemmatalis MNPV	-	AgMNPV-36	Brito A F.	Yes	KR815465.1	KR815465.1	KR815465.1	KR815465.1
Anticarsia gemmatalis MNPV	-	AgMNPV-37	Brito A F.	Yes	KR815466.1	KR815466.1	KR815466.1	KR815466.1
Anticarsia gemmatalis MNPV	-	AgMNPV-38	Brito A F.	Yes	KR815467.1	KR815467.1	KR815467.1	KR815467.1
Anticarsia gemmatalis MNPV	-	AgMNPV-39	Brito A F.	Yes	KR815468.1	KR815468.1	KR815468.1	KR815468.1
Anticarsia gemmatalis MNPV	-	AgMNPV-40	Brito A F.	Yes	KR815469.1	KR815469.1	KR815469.1	KR815469.1
Anticarsia gemmatalis MNPV	-	AgMNPV-42	Brito A F.	Yes	KR815470.1	KR815470.1	KR815470.1	KR815470.1
Anticarsia gemmatalis MNPV	-	AgMNPV-43	Brito A F.	Yes	KR815471.1	KR815471.1	KR815471.1	KR815471.1
Anticarsia gemmatalis MNPV	-	AgMNPV-79	Ferreira B C.	No	-	-	KF765838.1	-
Anticarsia gemmatalis MNPV	-	AgMNPV-84_85	Ferreira B C.	No	-	-	KF765839.1	-
Anticarsia gemmatalis MNPV	-	AgMNPV-85_86	Ferreira B C.	No	-	-	KF765840.1	-
Anticarsia gemmatalis MNPV	-	AgMNPV-86_87	Ferreira B C.	No	-	-	KF765841.1	-
Anticarsia gemmatalis MNPV	-	AgMNPV-87_88	Ferreira B C.	No	-	-	KF765842.1	-
Anticarsia gemmatalis MNPV	-	AgMNPV-88_89	Ferreira B C.	No	-	-	KF765843.1	-
Anticarsia gemmatalis MNPV	-	AgMNPV-89_90	Ferreira B C.	No	-	-	KF765844.1	-
Anticarsia gemmatalis MNPV	-	AgMNPV-90_91	Ferreira B C.	No	-	-	KF765845.1	-
Anticarsia gemmatalis MNPV	-	AgMNPV-91_92	Ferreira B C.	No	-	-	KF765846.1	-
Anticarsia gemmatalis MNPV	-	AgMNPV-92_93	Ferreira B C.	No	-	-	KF765847.1	-
Anticarsia gemmatalis MNPV	-	AgMNPV-93_94	Ferreira B C.	No	-	-	KF765848.1	-
Anticarsia gemmatalis MNPV	-	AgMNPV-94_95	Ferreira B C.	No	-	-	KF765849.1	-
Anticarsia gemmatalis MNPV	-	AgMNPV-95_96	Ferreira B C.	No	-	-	KF765850.1	-
Anticarsia gemmatalis MNPV	-	AgMNPV-96_97	Ferreira B C.	No	-	-	KF765851.1	-
Anticarsia gemmatalis MNPV	-	AgMNPV-97_98	Ferreira B C.	No	-	-	KF765852.1	-
Anticarsia gemmatalis MNPV	-	AgMNPV-98_99	Ferreira B C.	No	-	-	KF765853.1	-
Anticarsia gemmatalis MNPV	-	AgMNPV-99_00	Ferreira B C.	No	-	-	KF765854.1	-
Anticarsia gemmatalis MNPV	-	AgMNPV-Arg	Ferreira B C.	No	-	-	KF765855.1	-
Anticarsia gemmatalis MNPV	-	AgMNPV-CM	Ferreira B C.	No	-	-	KF765856.1	-
Anticarsia gemmatalis MNPV	-	AgMNPV-Dour	Ferreira B C.	No	-	-	KF765857.1	-
Anticarsia gemmatalis MNPV	-	AgMNPV-Ibip	Ferreira B C.	No	-	-	KF765858.1	-

Anticarsia gemmatalis MNPV	-	AgMNPV-Lond	Ferreira B C.	No	-	-	KF765859.1	-
Anticarsia gemmatalis MNPV	-	AgMNPV-PF	Ferreira B C.	No	-	-	KF765861.1	-
Anticarsia gemmatalis MNPV	-	AgMNPV-Pelot	Ferreira B C.	No	-	-	KF765860.1	-
Anticarsia gemmatalis MNPV	-	AgMNPV-Urug	Ferreira B C.	No	-	-	KF765862.1	-
Anticarsia gemmatalis NPV	2D	-	Zanotto P M.	No	-	-	-	Y17753.2
Apocheima cinerarium NPV	-	S7	Jehle J A.	No	AY706541.1	AY706606.1	-	AY706688.1
Apocheima cinerarium NPV	-	-	Qu L J.	No	-	-	-	DQ238113.1
Apocheima cinerarium NPV	-	-	Zhang Y A.	Yes	FJ914221.1	FJ914221.1	FJ914221.1	FJ914221.1
Aporia crataegi NPV	-	M45-3	Lange M.	No	AY519211.1	AY519212.1	-	AY519210.1
Archips cerasivoranus NPV	-	-	Rieth A.	No	-	-	-	U40834.1
Archips rosanus NPV	-	A8no2	Jehle J A.	No	DQ231335.1	DQ231334.1	-	-
Aroa discalis NPV	63	-	Herniou E A/Thézé J.	No	MH454115	MH454153	-	MH454193
Artogeia rapae GV	-	Wuhan	Wang X F.	Yes	GQ884143.1	GQ884143.1	GQ884143.1	GQ884143.1
Attacus ricini NPV	-	-	Hu J.	No	-	-	-	S68462.1
Autographa biloba NPV	-	-	Jehle J A.	No	AY737722.1	AY737723.1	-	AY737724.1
Autographa californica MNPV	-	1004	Rowley D L.	No	JN674655.1	JN674818.1	-	JN674794.1
Autographa californica MNPV	-	1176	Rowley D L.	No	JN674656.1	JN674819.1	-	JN674795.1
Autographa californica MNPV	-	1180	Rowley D L.	No	JN674657.1	JN674820.1	-	JN674796.1
Autographa californica MNPV	-	1199	Rowley D L.	No	JN674658.1	JN674821.1	-	JN674797.1
Autographa californica MNPV	-	1361	Rowley D L.	No	JN674659.1	JN674822.1	-	JN674798.1
Autographa californica MNPV	-	1412	Rowley D L.	No	JN674660.1	JN674823.1	-	JN674799.1
Autographa californica MNPV	-	1417	Rowley D L.	No	JN674661.1	JN674824.1	-	JN674800.1
Autographa californica MNPV	-	162	Rowley D L.	No	HQ246151.1	HQ246021.1	-	HQ246018.1
Autographa californica MNPV	-	1756	Rowley D L.	No	JN674662.1	JN674825.1	-	JN674801.1
Autographa californica MNPV	-	2162	Rowley D L.	No	JN674663.1	JN674826.1	-	JN674802.1
Autographa californica MNPV	-	228	Rowley D L.	No	JN674643.1	JN674806.1	-	JN674782.1
Autographa californica MNPV	-	282	Rowley D L.	No	JN674645.1	JN674808.1	-	JN674784.1
Autographa californica MNPV	-	3001	Rowley D L.	No	JN674664.1	JN674827.1	-	JN674803.1
Autographa californica MNPV	-	3035	Rowley D L.	No	HQ246153.1	HQ246023.1	-	HQ246020.1
Autographa californica MNPV	-	3092	Rowley D L.	No	JN674665.1	JN674828.1	-	JN674804.1
Autographa californica MNPV	-	3114	Rowley D L.	No	JN674644.1	JN674807.1	-	JN674783.1
Autographa californica MNPV	-	396	Rowley D L.	No	JN674647.1	JN674810.1	-	JN674786.1
Autographa californica MNPV	-	397	Rowley D L.	No	JN674648.1	JN674811.1	-	JN674787.1
Autographa californica MNPV	-	458	Rowley D L.	No	HQ246152.1	HQ246022.1	-	HQ246019.1
Autographa californica MNPV	-	465	Rowley D L.	No	JN674642.1	JN674805.1	-	JN674781.1
Autographa californica MNPV	-	555	Rowley D L.	No	JN674650.1	JN674813.1	-	JN674789.1
Autographa californica MNPV	-	570	Rowley D L.	No	JN674651.1	JN674814.1	-	JN674790.1
Autographa californica MNPV	-	578	Rowley D L.	No	JN674652.1	JN674815.1	-	JN674791.1
Autographa californica MNPV	-	582	Rowley D L.	No	JN674653.1	JN674816.1	-	JN674792.1
Autographa californica MNPV	-	683	Rowley D L.	No	JN674654.1	JN674817.1	-	JN674793.1
Autographa californica MNPV	-	A12-2	Jehle J A.	No	AY706528.1	AY706593.1	-	AY706681.1
Autographa californica MNPV	-	Biotrol_VTN	Rowley D L.	No	JN674646.1	JN674809.1	-	JN674785.1
Autographa californica MNPV	-	S43	Jehle J A.	No	AY706526.1	AY706591.1	-	AY706679.1
Autographa californica MNPV	-	WP10	Chateigner A.	Yes	KM609482.1	KM609482.1	KM609482.1	KM609482.1
Autographa californica NPV	C6	-	Possee R D.	No	-	-	-	M75679.1
Autographa californica NPV	C6	-	Tao X Y.	Yes	KF022001.1	KF022001.1	KF022001.1	KF022001.1
Autographa californica NPV	E2	-	Guarino L A.	No	-	-	M96361.1	-
Autographa californica NPV	E2	-	Maghodia A B.	Yes	KM667940.1	KM667940.1	KM667940.1	KM667940.1
Autographa californica NPV	L-1	-	Passarelli A L.	No	U04879.1	-	-	-
Autographa californica NPV	Vail_8	-	Popham H J R.	No	-	-	-	AF025997.1
Autographa californica NPV	-	-	Ayres M D.	Yes	L22858.1	L22858.1	L22858.1	L22858.1
Autographa californica NPV	-	-	Carstens E B.	No	-	-	-	M13056.1
Autographa californica NPV	-	-	Gearing K L.	No	-	-	-	D00700.1
Autographa californica NPV	-	-	Gonzalez M A.	No	-	-	-	M25054.1
Autographa californica NPV	-	-	Hooft van Iddekinge B J.	No	-	-	-	K01149.1
Autographa californica NPV	-	-	Pennock G D.	No	-	-	-	K02700.1
Autographa californica NPV	-	vAcRev-1	Wu C.	Yes	KU697902.1	-	KU697902.1	KU697902.1
Autographa californica NPV	-	vAcRev-2	Wu C.	Yes	KU697903.1	KU697903.1	KU697903.1	KU697903.1
Autographa californica NPV-B	-	1059	Rowley D L.	No	JN674669.1	JN674670.1	-	JN674671.1
Autographa gamma GV	-	M39-3	Lange M.	No	AY519214.1	AY519215.1	-	AY519213.1
Autographa gamma NPV	-	474	Rowley D L.	No	JN674649.1	JN674812.1	-	JN674788.1
Autographa nigrisigna NPV	Mz-A	-	Mukawa S.	No	AB465489.1	AB465490.1	-	AB465488.1
Autographa nigrisigna NPV	Mz-B	-	Mukawa S.	No	AB465492.1	AB465493.1	-	AB465491.1
Boarmia bistortata NPV	-	A5-4	Lange M.	No	AY519220.1	AY519221.1	-	AY519219.1
Bombyx mandarina NPV	-	S1	Xu Y P.	Yes	FJ882854.1	FJ882854.1	FJ882854.1	FJ882854.1
Bombyx mandarina NPV	-	-	Weide S.	No	-	-	-	DQ483053.1
Bombyx mandarina NPV S2	-	-	Xu Y P.	Yes	JQ071499.1	JQ071499.1	JQ071499.1	JQ071499.1
Bombyx mori NPV	Brazilian	-	Ardisson-Araujo D M.	Yes	KJ186100.1	KJ186100.1	KJ186100.1	KJ186100.1
Bombyx mori NPV	Cubic	-	Cheng R L.	Yes	JQ991009.1	JQ991009.1	JQ991009.1	JQ991009.1
Bombyx mori NPV	Guangxi	-	Xu Y P.	Yes	JQ991011.1	JQ991011.1	JQ991011.1	JQ991011.1
Bombyx mori NPV	India	-	Fan H W.	Yes	JQ991010.1	JQ991010.1	JQ991010.1	JQ991010.1
Bombyx mori NPV	K1	-	Kang S K.	No	-	-	-	U75359.1
Bombyx mori NPV	Thailand	-	Zhou J B.	No	-	-	-	JN862922.1
Bombyx mori NPV	YN1	-	Tang F F.	No	-	-	-	KR139829.1
Bombyx mori NPV	Zhejiang	-	Xu Y P.	Yes	JQ991008.1	JQ991008.1	JQ991008.1	JQ991008.1
Bombyx mori NPV	-	C1	Kim M.	Yes	KF306215.1	KF306215.1	KF306215.1	KF306215.1
Bombyx mori NPV	-	C2	Choi J H.	Yes	KF306216.1	KF306216.1	KF306216.1	KF306216.1
Bombyx mori NPV	-	C6	Kim M.	Yes	KF306217.1	KF306217.1	KF306217.1	KF306217.1
Bombyx mori NPV	-	D1	Hashimoto Y.	No	AB009987.1	-	-	-
Bombyx mori NPV	-	GXBB	Liang X.	No	-	-	-	JQ291662.1
Bombyx mori NPV	-	GXBY	Liang X.	No	-	-	-	JQ291663.1
Bombyx mori NPV	-	GXCW	Liang X.	No	-	-	-	JQ291664.1
Bombyx mori NPV	-	GXDA	Liang X.	No	-	-	-	JQ291665.1
Bombyx mori NPV	-	GXFM	Liang X.	No	-	-	-	JQ291666.1
Bombyx mori NPV	-	GXGB	Liang X.	No	-	-	-	JQ291667.1
Bombyx mori NPV	-	GXGN	Liang X.	No	-	-	-	JQ291668.1
Bombyx mori NPV	-	GXHJ	Liang X.	No	-	-	-	JQ291669.1
Bombyx mori NPV	-	GXHP	Liang X.	No	-	-	-	JQ291670.1
Bombyx mori NPV	-	GXHS	Liang X.	No	-	-	-	JQ291671.1
Bombyx mori NPV	-	GXHX	Liang X.	No	-	-	-	JQ291672.1
Bombyx mori NPV	-	GXLJ	Liang X.	No	-	-	-	JQ291676.1
Bombyx mori NPV	-	GXLS	Liang X.	No	-	-	-	JQ291677.1
Bombyx mori NPV	-	GXLeY	Liang X.	No	-	-	-	JQ291673.1
Bombyx mori NPV	-	GXLingY	Liang X.	No	-	-	-	JQ291674.1
Bombyx mori NPV	-	GXLiuC	Liang X.	No	-	-	-	JQ291675.1
Bombyx mori NPV	-	GXLuoC1	Liang X.	No	-	-	-	JQ291678.1
Bombyx mori NPV	-	GXLuoC2	Liang X.	No	-	-	-	JQ291679.1
Bombyx mori NPV	-	GXMS1	Liang X.	No	-	-	-	JQ291680.1
Bombyx mori NPV	-	GXMS2	Liang X.	No	-	-	-	JQ291681.1
Bombyx mori NPV	-	GXNM	Liang X.	No	-	-	-	JQ291682.1
Bombyx mori NPV	-	GXNN	Liang X.	No	-	-	-	JQ291683.1
Bombyx mori NPV	-	GXNP	Liang X.	No	-	-	-	JQ291684.1
Bombyx mori NPV	-	GXPB	Liang X.	No	-	-	-	JQ291685.1
Bombyx mori NPV	-	GXPG	Liang X.	No	-	-	-	JQ291686.1
Bombyx mori NPV	-	GXPN	Liang X.	No	-	-	-	JQ291687.1
Bombyx mori NPV	-	GXQT	Liang X.	No	-	-	-	JQ291688.1
Bombyx mori NPV	-	GXRA	Liang X.	No	-	-	-	JQ291689.1
Bombyx mori NPV	-	GXRS	Liang X.	No	-	-	-	JQ291690.1
Bombyx mori NPV	-	GXRX	Liang X.	No	-	-	-	JQ291691.1
Bombyx mori NPV	-	GXSL	Liang X.	No	-	-	-	JQ291692.1
Bombyx mori NPV	-	GXTL	Liang X.	No	-	-	-	JQ291693.1
Bombyx mori NPV	-	GXTX	Liang X.	No	-	-	-	JQ291694.1
Bombyx mori NPV	-	GXWM	Liang X.	No	-	-	-	JQ291695.1
Bombyx mori NPV	-	GXWX	Liang X.	No	-	-	-	JQ291696.1
Bombyx mori NPV	-	GXXC	Liang X.	No	-	-	-	JQ291697.1

Bombyx mori NPV	-	GXXD1	Liang X.	No	-	-	-	JQ291698.1
Bombyx mori NPV	-	GXXD2	Liang X.	No	-	-	-	JQ291699.1
Bombyx mori NPV	-	GXXZ	Liang X.	No	-	-	-	JQ291700.1
Bombyx mori NPV	-	GXYF	Liang X.	No	-	-	-	JQ291701.1
Bombyx mori NPV	-	GXYJ	Liang X.	No	-	-	-	JQ291702.1
Bombyx mori NPV	-	GXYZ1	Liang X.	No	-	-	-	JQ291703.1
Bombyx mori NPV	-	GXYZ2	Liang X.	No	-	-	-	JQ291704.1
Bombyx mori NPV	-	GXZP	Liang X.	No	-	-	-	JQ291705.1
Bombyx mori NPV	-	GXZS	Liang X.	No	-	-	-	JQ291706.1
Bombyx mori NPV	-	H4	Bando H.	Yes	LC150780.1	LC150780.1	LC150780.1	LC150780.1
Bombyx mori NPV	-	M28-4	Lange M.	No	AY519217.1	AY519218.1	-	AY519216.1
Bombyx mori NPV	-	S12	Jehle J A.	No	DQ231340.1	DQ231341.1	-	DQ231339.1
Bombyx mori NPV	-	S9	Jehle J A.	No	DQ231337.1	DQ231338.1	-	DQ231336.1
Bombyx mori NPV	-	T3	Kamita S G.	Yes	L33180.1	L33180.1	L33180.1	L33180.1
Bombyx mori NPV	-	-	Chu R.	No	-	-	-	X63614.1
Bombyx mori NPV	-	-	Iatrou K.	No	-	-	-	M10043.1
Bombyx mori NPV	-	-	Kaewwises M.	No	-	-	-	AY779044.1
Bombyx mori NPV	-	-	Maeda S.	No	-	-	-	M30925.1
Busseola fusca NPV	-	A2-4	Lange M.	No	AY519223.2	AY519224.1	-	AY519222.1
Buzura suppressaria NPV	HB	-	Hu Z H.	No	-	-	-	X70844.1
Buzura suppressaria NPV	-	GX-BsNPV	Luo J.	No	KJ685549.1	-	-	-
Buzura suppressaria NPV	-	Guangxi	Luo J.	Yes	KM986882.1	KM986882.1	KM986882.1	KM986882.1
Buzura suppressaria NPV	-	Hubei	Hu Z H.	Yes	KF611977.1	KF611977.1	KF611977.1	KF611977.1
Buzura suppressaria NPV	-	S13	Jehle J A.	No	DQ231343.1	DQ231344.1	-	DQ231342.1
Buzura suppressaria NPV	-	Terai_K1	Ghosh B.	No	-	-	-	KX665535.2
Buzura suppressaria NPV	-	-	Antony B.	No	-	-	-	JF510034.1
Caloptilia theivora GV	-	-	Kouassi L N.	No	AB598365.1	AB598366.1	-	AB598364.1
Catopsilia pomona NPV	-	416	Wang J.	Yes	KU565883.1	KU565883.1	KU565883.1	KU565883.1
Catopsilia pomona NPV	-	S16	Jehle J A.	No	DQ231346.1	DQ231345.1	-	-
Cerapteryx graminis NPV	V1	-	Graham R I.	No	HQ603183.1	HQ603184.1	-	HQ603182.1
Choristoneura fumiferana DEF MNPV	-	-	Li X.	Yes	AY327402.2	AY327402.2	AY327402.2	AY327402.2
Choristoneura fumiferana GV	-	-	Bah A.	No	-	-	-	AF439352.1
Choristoneura fumiferana GV	-	-	Escasa S R.	Yes	DQ333351.1	DQ333351.1	DQ333351.1	DQ333351.1
Choristoneura fumiferana GV	-	-	Rashidan K K.	No	-	-	AF538602.1	-
Choristoneura fumiferana MNPV	Ireland	-	Poloumienko A.	No	U70991.1	-	-	-
Choristoneura fumiferana MNPV	T3-NPV	Tortrivirus	Rieth A.	No	-	-	-	U40833.1
Choristoneura fumiferana MNPV	-	-	Lee H Y.	Yes	AF512031.3	AF512031.3	AF512031.3	AF512031.3
Choristoneura murinana GV	-	A11-1_M50-3	Jehle J A.	No	AY706543.1	AY706608.1	-	AY706663.1
Choristoneura murinana NPV	Darmstadt	-	Rohrmann G F.	Yes	KF894742.1	KF894742.1	KF894742.1	KF894742.1
Choristoneura murinana NPV	-	26	Rohrmann G F.	No	KC798395.1	-	-	-
Choristoneura occidentalis GV	-	British_Columbia_2006	Graham R I.	No	-	-	-	EU201036.1
Choristoneura occidentalis NPV	-	BC_1	Thumbi D K.	Yes	KC961303.1	KC961303.1	KC961303.1	KC961303.1
Choristoneura occidentalis NPV	-	British_Columbia_2006	Graham R I.	No	-	-	-	EU201037.1
Choristoneura rosaceana NPV	-	NB_1	Thumbi D K.	Yes	KC961304.1	KC961304.1	KC961304.1	KC961304.1
Choristoneura rosaceana NPV	-	-	Lucarotti C J.	No	-	-	-	U91940.1
Choristoneura viridis GV	-	22	Rohrmann G F.	No	KC798394.1	-	-	-
Chrysodeixis chalcites NPV	TF1	1	Bernal A.	Yes	JX560539.1	JX560539.1	JX560539.1	JX560539.1
Chrysodeixis chalcites NPV	TF1	2	Bernal A.	Yes	JX560541.1	JX560540.1	JX560540.1	JX560540.1
Chrysodeixis chalcites NPV	TF1	3	Bernal A.	Yes	JX560542.1	JX560541.1	JX560541.1	JX560541.1
Chrysodeixis chalcites NPV	TF1	4	Bernal A.	Yes	-	JX560542.1	JX560542.1	JX560542.1
Chrysodeixis chalcites NPV	TF1	-	Bernal A.	No	JX560540.1	-	-	-
Chrysodeixis chalcites NPV	-	-	Xu F.	No	-	-	-	EU401915.1
Chrysodeixis chalcites NPV	-	-	van Oers M M.	Yes	AY864330.1	AY864330.1	AY864330.1	AY864330.1
Chrysodeixis chalcites NPV TF1-A	-	-	Bernal A.	Yes	JX535500.1	JX535500.1	JX535500.1	JX535500.1
Chrysodeixis includens NPV	-	1	Craveiro S R.	No	-	KC136327.1	KC136320.1	-
Chrysodeixis includens NPV	-	2	Craveiro S R.	No	-	KU669294.1	KU669294.1	-
Chrysodeixis includens NPV	-	GO	Morgado F S.	No	KX255634.1	KX255635.1	-	-
Chrysodeixis includens NPV	-	IA	Craveiro S R.	Yes	KU669289.1	JQ260772.1	JQ260786.1	KU669289.1
Chrysodeixis includens NPV	-	IB	Craveiro S R.	Yes	KU669290.1	JQ260773.1	JQ260787.1	KU669290.1
Chrysodeixis includens NPV	-	IC	Craveiro S R.	Yes	KU669291.1	JQ260774.1	JQ260788.1	KU669291.1
Chrysodeixis includens NPV	-	ID	Craveiro S R.	Yes	KU669292.1	JQ260775.1	JQ260789.1	KU669292.1
Chrysodeixis includens NPV	-	IE	Craveiro S R.	No	JQ260769.1	JQ260776.1	KC136318.1	JQ260797.1
Chrysodeixis includens NPV	-	IF	Craveiro S R.	Yes	KU669293.1	JQ260777.1	JQ260791.1	KU669293.1
Chrysodeixis includens NPV	-	IG	Craveiro S R.	Yes	KU669294.1	JQ260778.1	JQ260792.1	KU669294.1
Clanis bilineata NPV	-	DZ1	Zhu S Y.	Yes	DQ504428.1	DQ504428.1	DQ504428.1	DQ504428.1
Clostera anachoreta GV	-	ClanGV-HBHN	Liang Z.	Yes	HQ116624.1	HQ116624.1	HQ116624.1	HQ116624.1
Clostera anachoreta GV	-	S49	Jehle J A.	No	AY706544.1	AY706609.1	-	AY706664.1
Clostera anachoreta GV	-	-	Zhang X X.	No	AY664543.1	-	-	AY880963.1
Clostera anastomosis GV B	ClasGV-B	-	Yin F.	Yes	KR091910.1	KR091910.1	KR091910.1	KR091910.1
Clostera anastomosis GV Henan	-	CaLGV-Henan	Liang Z.	Yes	KC179784.1	KC179784.1	KC179784.1	KC179784.1
Cnaphalocrocis medinalis GV	Enping	-	Zhang S.	Yes	KU593505.1	KU593505.1	KU593505.1	KU593505.1
Cnaphalocrocis medinalis GV	-	-	Han G.	Yes	KP658210.1	KP658210.1	KP658210.1	KP658210.1
Cnephasia longana GV	-	A2-2	Lange M.	No	AY519226.1	AY519227.1	-	AY519225.1
Coloradia pandora NPV	-	19	Rohrmann G F.	No	KC798393.1	-	-	-
Coloradia pandora NPV	-	M30-2	Lange M.	No	AY519229.1	AY519230.1	-	AY519228.1
Condylorrhiza vestigialis MNPV	-	-	Castro M E.	Yes	KJ631623.1	KJ631623.1	KJ631623.1	KJ631623.1
Cryptophlebia leucotreta GV	-	CV3	Jehle J A.	Yes	AY229987.1	AY229987.1	AY229987.1	AY229987.1
Cryptophlebia leucotreta GV	-	CrleGV-SA	Singh S.	No	-	-	-	AY293731.1
Cryptophlebia leucotreta GV	-	CrleGV-SA	van der Merwe M.	Yes	MF974563.1	MF974563.1	MF974563.1	MF974563.1
Culex nigripalpus NPV	-	Florida1997	Afonso C L.	Yes	AF403738.1	AF403738.1	AF403738.1	-
Cyclophragma undans NPV	-	Whiov	Zhu Z.	Yes	KT957089.1	KT957089.1	KT957089.1	KT957089.1
Cydia pomonella GV	CJ01	-	Shen J.	No	-	-	-	JQ003556.1
Cydia pomonella GV	Mexican_1	-	Crook N E.	Yes	U53466.2	U53466.2	U53466.2	U53466.2
Cydia pomonella GV	-	217	Arneodo J D.	No	KF584307.1	-	-	KF584295.1
Cydia pomonella GV	-	38	Arneodo J D.	No	KF584306.1	-	-	KF584294.1
Cydia pomonella GV	-	616	Arneodo J D.	No	KF584305.1	-	-	KF584293.1
Cydia pomonella GV	-	69	Arneodo J D.	No	KF584304.1	-	-	KF584292.1
Cydia pomonella GV	-	A11-2	Jehle J A.	No	AY706561.1	AY706626.1	-	AY706670.1
Cydia pomonella GV	-	A6-4	Jehle J A.	No	AY706556.1	AY706621.1	-	AY706667.1
Cydia pomonella GV	-	ALE1	Fan J B.	No	-	-	-	KJ184164.1
Cydia pomonella GV	-	ALE2	Fan J B.	No	-	-	-	KJ184165.1
Cydia pomonella GV	-	C1	Arneodo J D.	No	KF584302.1	-	-	KF584290.1
Cydia pomonella GV	-	C6	Arneodo J D.	No	KF584303.1	-	-	KF584291.1
Cydia pomonella GV	-	Col19	Arneodo J D.	No	KF584310.1	-	-	KF584298.1
Cydia pomonella GV	-	CpGV-E2	Gebhardt M M.	Yes	KM217577.1	KM217577.1	KM217577.1	KM217577.1
Cydia pomonella GV	-	CpGV-I07	Gebhardt M M.	Yes	KM217574.1	KM217574.1	KM217574.1	KM217574.1
Cydia pomonella GV	-	CpGV-I12	Gebhardt M M.	Yes	KM217576.1	KM217576.1	KM217576.1	KM217576.1
Cydia pomonella GV	-	CpGV-M	Gebhardt M M.	Yes	KM217575.1	KM217575.1	KM217575.1	KM217575.1
Cydia pomonella GV	-	CpGV-S	Gebhardt M M.	Yes	KM217573.1	KM217573.1	KM217573.1	KM217573.1
Cydia pomonella GV	-	E2	Eberle K E.	No	EU428825.1	-	-	EU428824.1
Cydia pomonella GV	-	G01	Eberle K E.	No	EU370241.1	-	-	EU370250.1
Cydia pomonella GV	-	G01	Sayed S.	No	-	EU370259.1	-	-
Cydia pomonella GV	-	G02	Eberle K E.	No	EU370242.1	-	-	EU370249.1
Cydia pomonella GV	-	G02	Sayed S.	No	-	EU370260.1	-	-
Cydia pomonella GV	-	I01	Eberle K E.	No	EU370235.1	-	-	EU370243.1
Cydia pomonella GV	-	I01	Sayed S.	No	-	EU370253.1	-	-
Cydia pomonella GV	-	I07	Eberle K E.	No	EU370237.1	-	-	EU370245.1
Cydia pomonella GV	-	I07	Sayed S.	No	-	EU370255.1	-	-
Cydia pomonella GV	-	I08	Eberle K E.	No	EU370236.1	-	-	EU370244.1
Cydia pomonella GV	-	I08	Sayed S.	No	-	EU370254.1	-	-
Cydia pomonella GV	-	I12	Eberle K E.	No	EU370238.1	-	-	EU370246.1
Cydia pomonella GV	-	I12	Sayed S.	No	-	EU370256.1	-	-
Cydia pomonella GV	-	I66	Eberle K E.	No	EU370239.1	-	-	EU370248.1
Cydia pomonella GV	-	I66	Sayed S.	No	-	EU370257.1	-	-
Cydia pomonella GV	-	I68	Eberle K E.	No	EU370240.1	-	-	EU370247.1

Cydia pomonella GV	-	I68	Sayed S.	No	-	EU370258.1	-	-
Cydia pomonella GV	-	KS1	Fan J B.	No	-	-	-	KJ184166.1
Cydia pomonella GV	-	M10	Arneodo J D.	No	KF584300.1	-	-	KF584288.1
Cydia pomonella GV	-	M18	Arneodo J D.	No	KF584299.1	-	-	KF584287.1
Cydia pomonella GV	-	M3	Arneodo J D.	No	KF584301.1	-	-	KF584289.1
Cydia pomonella GV	-	M39-1	Jehle J A.	No	AY706557.1	AY706622.1	-	AY706668.1
Cydia pomonella GV	-	P118	Arneodo J D.	No	KF584308.1	-	-	KF584296.1
Cydia pomonella GV	-	P7	Arneodo J D.	No	KF584309.1	-	-	KF584297.1
Cydia pomonella GV	-	WW1	Fan J B.	No	-	-	-	KJ184163.1
Cydia pomonella GV	-	ZY1	Fan J B.	No	-	-	-	KJ184162.1
Cynosarga chrysolopa NPV	302	-	Herniou E A/Thézé J.	No	MH454116	MH454154	-	MH454194
Darna trima GV	545	-	Herniou E A/Thézé J.	No	MH454117	MH454155	MH458175	MH454195
Dasychira plagiata NPV	-	M36-8	Jehle J A.	No	AY706545.1	AY706610.1	-	AY706690.1
Dasychira pudibunda NPV	-	ML1	Krejmer M.	Yes	KP747440.1	KP747440.1	KP747440.1	KP747440.1
Dendrolimus kikuchii NPV	YN	-	Yang M M.	Yes	JX193905.1	JX193905.1	JX193905.1	JX193905.1
Diaphania pulverulentalis NPV	-	-	Priyadharshini P.	No	-	-	-	FJ901339.1
Diatraea saccharalis GV	Parana-2009	DisaGV-Parana-2009	Ardisson-Araujo D M.	Yes	KP296186.1	KP296186.1	KP296186.1	KP296186.1
Dione juno MNPV	tmk1	1	Rodriguez V A.	No	HQ010111.1	HQ010112.1	-	HQ010113.1
Dione juno MNPV	tmk1	2	Rodriguez V A.	No	HQ010114.1	HQ010115.1	-	HQ010116.1
Dirphia peruvianus NPV	-	A3-1	Jehle J A.	No	AY706546.1	AY706611.1	-	AY706691.1
Ectropis grisescens NPV	-	S22	Jehle J A.	No	AY706547.1	AY706612.1	-	AY706692.1
Ectropis obliqua NPV	A1	-	Ma X C.	Yes	DQ837165.1	DQ837165.1	DQ837165.1	DQ837165.1
Ectropis obliqua NPV	unioasis_1	-	Chen J.	Yes	KC960018.1	KC960018.1	KC960018.1	KC960018.1
Cadra cautella NPV	c5	-	Herniou E A/Thézé J.	No	MH454118	MH454156	MH458176	MH454196
Epinotia aporema GV	-	-	Ferrelli M L.	Yes	JN408834.1	JN408834.1	JN408834.1	JN408834.1
Epinotia aporema GV	-	-	Parola A D.	No	-	-	-	AF473703.1
Epinotia granitalis NPV	-	A1	Takatsuka J.	No	AB300385.1	AB300386.1	-	AB300384.1
Epiphyas postvittana NPV	-	-	Hyink O.	Yes	AY043265.1	AY043265.1	AY043265.1	-
Erannis defoliaria NPV	174	-	Herniou E A/Thézé J.	No	MH454119	MH454157	-	MH454197
Erinnyis ello GV	-	ErelGV-00	Brito A F.	Yes	KX859082.1	KX859082.1	KX859082.1	KX859082.1
Erinnyis ello GV	-	ErelGV-94	Brito A F.	Yes	KX859079.1	KX859079.1	KX859079.1	KX859079.1
Erinnyis ello GV	-	ErelGV-98	Brito A F.	Yes	KX859080.1	KX859080.1	KX859080.1	KX859080.1
Erinnyis ello GV	-	ErelGV-99	Brito A F.	Yes	KX859081.1	KX859081.1	KX859081.1	KX859081.1
Erinnyis ello GV	-	ErelGV-AC	Brito A F.	Yes	KX859083.1	KX859083.1	KX859083.1	KX859083.1
Erinnyis ello GV	-	ErelGV-PA	Brito A F.	Yes	KX859084.1	KX859084.1	KX859084.1	KX859084.1
Erinnyis ello GV	-	M34-4	Jehle J A.	No	AY706548.1	AY706613.1	-	AY706665.1
Erinnyis ello GV	-	S86	Ardisson-Araujo D M.	Yes	KJ406702.1	KJ406702.1	KJ406702.1	KJ406702.1
Estigmene acrea GV	-	M30-3	Jehle J A.	No	-	DQ235251.1	-	DQ235250.1
Euplexia lucipara GV	248	-	Herniou E A/Thézé J.	No	MH454120	MH454158	-	MH454198
Euproctis chrysorrhoea NPV	a10	-	Herniou E A/Thézé J.	No	MH454121	MH454159	MH458177	MH454199
Euproctis digramma NPV	-	S24	Jehle J A.	No	AY706549.1	AY706614.1	-	AY706693.1
Euproctis pseudoconspersa NPV	Hangzhou	-	Tang X D.	Yes	FJ227128.1	FJ227128.1	FJ227128.1	FJ227128.1
Euproctis pseudoconspersa NPV	-	A13-1	Jehle J A.	No	AY706551.1	-	-	AY706694.1
Euproctis pseudoconspersa NPV	-	A4-5	Jehle J A.	No	-	AY706616.1	-	-
Euproctis similis NPV	768	-	Herniou E A/Thézé J.	No	MH454122	MH454160	MH458178	MH454200
Euxoa ochrogaster GV	-	A24-1	Jehle J A.	No	AY706550.1	AY706615.1	-	AY706666.1
Galleria mellonella MNPV	-	1138	Rowley D L.	No	JN674666.1	JN674667.1	-	JN674668.1
Galleria mellonella MNPV	-	A11-3	Jehle J A.	No	AY706553.1	AY706618.1	-	AY706696.1
Galleria mellonella MNPV	-	A16-3	Jehle J A.	No	AY706554.1	AY706619.1	-	AY706697.1
Galleria mellonella MNPV	-	A3-6	Jehle J A.	No	AY706555.1	AY706620.1	-	AY706698.1
Harrisina brillians GV	m2	-	Herniou E A/Thézé J.	No	AY449801.1	MH454161	AY449780.1	MH454201
Heliconius erato NPV	789	-	Herniou E A/Thézé J.	No	AY449792.1	MH454162	AY449771.1	MH454202
Helicoverpa NPV AC53	AC53	-	Noune C.	Yes	KJ909666.1	KJ909666.1	KJ909666.1	KJ909666.1
Helicoverpa NPV AC53	AC53C1	-	Noune C.	Yes	KU738896.1	KU738896.1	KU738896.1	KU738896.1
Helicoverpa NPV AC53	AC53C3	-	Noune C.	Yes	KU738897.1	KU738897.1	KU738897.1	KU738897.1
Helicoverpa NPV AC53	AC53C5	-	Noune C.	Yes	KU738898.1	KU738898.1	KU738898.1	KU738898.1
Helicoverpa NPV AC53	AC53C6	-	Noune C.	Yes	KU738899.1	KU738899.1	KU738899.1	KU738899.1
Helicoverpa NPV AC53	AC53C9	-	Noune C.	Yes	KU738900.1	KU738900.1	KU738900.1	KU738900.1
Helicoverpa NPV AC53	AC53T2	-	Noune C.	Yes	KU738901.1	KU738901.1	KU738901.1	KU738901.1
Helicoverpa NPV AC53	AC53T41	-	Noune C.	Yes	KU738902.1	KU738902.1	KU738902.1	KU738902.1
Helicoverpa NPV AC53	AC53T42	-	Noune C.	Yes	KU738903.1	KU738903.1	KU738903.1	KU738903.1
Helicoverpa NPV AC53	AC53T5	-	Noune C.	Yes	KU738904.1	KU738904.1	KU738904.1	KU738904.1
Helicoverpa armigera GV	-	-	Harrison R L.	Yes	EU255577.1	EU255577.1	EU255577.1	EU255577.1
Helicoverpa armigera MNPV	-	1072	Rowley D L.	No	HQ246044.1	HQ246036.1	-	HQ246028.1
Helicoverpa armigera MNPV	-	120	Rowley D L.	No	HQ246040.1	HQ246032.1	-	HQ246024.1
Helicoverpa armigera MNPV	-	131	Rowley D L.	No	HQ246041.1	HQ246033.1	-	HQ246025.1
Helicoverpa armigera MNPV	-	3110	Rowley D L.	No	HQ246045.1	HQ246037.1	-	HQ246029.1
Helicoverpa armigera MNPV	-	3153	Rowley D L.	No	HQ246046.1	HQ246038.1	-	HQ246030.1
Helicoverpa armigera MNPV	-	3154	Rowley D L.	No	HQ246047.1	HQ246039.1	-	HQ246031.1
Helicoverpa armigera MNPV	-	443	Rowley D L.	No	HQ246042.1	HQ246034.1	-	HQ246026.1
Helicoverpa armigera MNPV	-	449	Rowley D L.	No	HQ246043.1	HQ246035.1	-	HQ246027.1
Helicoverpa armigera MNPV	-	-	Tang P.	Yes	EU730893.1	EU730893.1	EU730893.1	EU730893.1
Helicoverpa armigera NPV	A6	-	Le T H.	No	-	-	-	U67258.1
Helicoverpa armigera NPV	AE20	-	Le T H.	No	-	-	-	U67257.1
Helicoverpa armigera NPV	Bangalore	HA_01	Jose J.	No	JN591550.1	-	-	-
Helicoverpa armigera NPV	Bathinda	-	Gupta V K.	No	FJ157288.1	-	-	FJ157292.1
Helicoverpa armigera NPV	E17	-	Le T H.	No	-	-	-	U67256.1
Helicoverpa armigera NPV	F29	-	Le T H.	No	-	-	-	U67255.1
Helicoverpa armigera NPV	Faridkot	-	Rakshit O.	Yes	KM357512.1	KM357515.1	-	KM357499.1
Helicoverpa armigera NPV	HAU	-	Gupta V K.	No	FJ157290.1	-	-	FJ157295.1
Helicoverpa armigera NPV	Jodhan	-	Gupta V K.	No	FJ157289.1	-	-	FJ157294.1
Helicoverpa armigera NPV	L1	HANPVL1	Rakshit O.	Yes	KT013224.1	KT013224.1	KT013224.1	KT013224.1
Helicoverpa armigera NPV	LB1	-	Arrizubieta M.	Yes	KJ701029.1	KJ701029.1	KJ701029.1	KJ701029.1
Helicoverpa armigera NPV	LB3	-	Arrizubieta M.	Yes	KJ701030.1	KJ701030.1	KJ701030.1	KJ701030.1
Helicoverpa armigera NPV	LB6	-	Arrizubieta M.	Yes	KJ701031.1	KJ701031.1	KJ701031.1	KJ701031.1
Helicoverpa armigera NPV	PAU	-	Gupta V K.	No	FJ157287.1	-	-	FJ157291.1
Helicoverpa armigera NPV	PDBC	-	Gupta V K.	No	FJ157286.1	-	-	FJ157293.1
Helicoverpa armigera NPV	SP1A	-	Arrizubieta M.	Yes	KJ701032.1	KJ701032.1	KJ701032.1	KJ701032.1
Helicoverpa armigera NPV	SP1B	-	Arrizubieta M.	Yes	KJ701033.1	KJ701033.1	KJ701033.1	KJ701033.1
Helicoverpa armigera NPV	-	1073	Rowley D L.	No	HQ246108.1	HQ246135.1	-	HQ246081.1
Helicoverpa armigera NPV	-	1113	Rowley D L.	No	HQ246109.1	HQ246136.1	-	HQ246082.1
Helicoverpa armigera NPV	-	1115	Rowley D L.	No	HQ246110.1	HQ246137.1	-	HQ246083.1
Helicoverpa armigera NPV	-	1186	Rowley D L.	No	HQ246112.1	HQ246139.1	-	HQ246085.1
Helicoverpa armigera NPV	-	1221	Rowley D L.	No	HQ246113.1	HQ246140.1	-	HQ246086.1
Helicoverpa armigera NPV	-	1240	Rowley D L.	No	HQ246114.1	HQ246141.1	-	HQ246087.1
Helicoverpa armigera NPV	-	126	Rowley D L.	No	HQ246099.1	HQ246126.1	-	HQ246072.1
Helicoverpa armigera NPV	-	138	Rowley D L.	No	HQ246100.1	HQ246127.1	-	HQ246073.1
Helicoverpa armigera NPV	-	141	Rowley D L.	No	HQ246101.1	HQ246128.1	-	HQ246074.1
Helicoverpa armigera NPV	-	1623	Rowley D L.	No	HQ246116.1	HQ246143.1	-	HQ246089.1
Helicoverpa armigera NPV	-	1625	Rowley D L.	No	HQ246117.1	HQ246144.1	-	HQ246090.1
Helicoverpa armigera NPV	-	1825	Rowley D L.	No	HQ246118.1	HQ246145.1	-	HQ246091.1
Helicoverpa armigera NPV	-	2066	Rowley D L.	No	HQ246119.1	HQ246146.1	-	HQ246092.1
Helicoverpa armigera NPV	-	2588	Rowley D L.	No	HQ246120.1	HQ246147.1	-	HQ246093.1
Helicoverpa armigera NPV	-	3010	Rowley D L.	No	HQ246121.1	HQ246148.1	-	HQ246094.1
Helicoverpa armigera NPV	-	3104	Rowley D L.	No	HQ246122.1	HQ246149.1	-	HQ246095.1
Helicoverpa armigera NPV	-	75	Rowley D L.	No	HQ246098.1	HQ246125.1	-	HQ246071.1
Helicoverpa armigera NPV	-	Bangalore	Jency J.	No	-	-	-	JQ612524.1
Helicoverpa armigera NPV	-	C1	Zhang C X.	Yes	AF303045.2	AF303045.2	AF303045.2	AF303045.2
Helicoverpa armigera NPV	-	Faridkot	Jency J.	No	-	-	-	KC174715.1
Helicoverpa armigera NPV	-	H25EA1	Noune C.	Yes	KJ922128.1	KJ922128.1	KJ922128.1	KJ922128.1
Helicoverpa armigera NPV	-	Ludhiana	Ashika T R.	No	-	-	-	KY432399.1
Helicoverpa armigera NPV	-	Ludhiana	Rakshit O.	No	-	-	-	KM268536.1
Helicoverpa armigera NPV	-	Palampur	Guleria S.	No	-	-	-	LK031772.1
Helicoverpa armigera NPV	-	hingoli	Ashika T R.	No	-	-	-	KF895395.1
Helicoverpa armigera NPV	-	-	Chen X.	No	-	-	-	U97657.1

Helicoverpa armigera NPV	-	-	Khan S.	No	AY118080.1	-	-	AF157012.2
Helicoverpa armigera NPV	-	-	Zhang C.	No	-	-	-	U95055.1
Helicoverpa armigera NPV G4	-	-	Deng F.	Yes	AF271059.2	AF271059.2	AF271059.2	AF271059.2
Helicoverpa armigera NPV	NNg1	-	Ogembo J G.	Yes	AP010907.1	AP010907.1	AP010907.1	AP010907.1
Helicoverpa armigera NPV	Australia	-	Zhang H.	Yes	JN584482.1	JN584482.1	JN584482.1	JN584482.1
Helicoverpa assulta NPV	-	Korean	Woo S D.	No	-	-	-	DQ157735.1
Helicoverpa gelotopoeon NPV	ar	-	Ferrelli M L.	No	KP340515.1	KP340516.1	-	KP340517.1
Helicoverpa zea NPV	Elkar	-	Le T H.	No	U67265.1	-	-	U67264.1
Helicoverpa zea NPV	Gemstar-35022	-	Rowley D L.	No	HQ246097.1	HQ246124.1	-	HQ246070.1
Helicoverpa zea NPV	HS-18	-	Ternovoi V A.	Yes	KJ004000.1	KJ004000.1	KJ004000.1	KJ004000.1
Helicoverpa zea NPV	-	1013	Rowley D L.	No	HQ246105.1	HQ246132.1	-	HQ246078.1
Helicoverpa zea NPV	-	1024	Rowley D L.	No	HQ246106.1	HQ246133.1	-	HQ246079.1
Helicoverpa zea NPV	-	1027	Rowley D L.	No	HQ246107.1	HQ246134.1	-	HQ246080.1
Helicoverpa zea NPV	-	1180	Rowley D L.	No	HQ246111.1	HQ246138.1	-	HQ246084.1
Helicoverpa zea NPV	-	1578	Rowley D L.	No	HQ246115.1	HQ246142.1	-	HQ246088.1
Helicoverpa zea NPV	-	3108	Rowley D L.	No	HQ246123.1	HQ246150.1	-	HQ246096.1
Helicoverpa zea NPV	-	543	Rowley D L.	No	HQ246102.1	HQ246129.1	-	HQ246075.1
Helicoverpa zea NPV	-	566	Rowley D L.	No	HQ246103.1	HQ246130.1	-	HQ246076.1
Helicoverpa zea NPV	-	668	Rowley D L.	No	HQ246104.1	HQ246131.1	-	HQ246077.1
Helicoverpa zea NPV	-	Br_South	Ardisson-Araujo D M P.	Yes	KM596835.1	KM596835.1	KM596835.1	KM596835.1
Helicoverpa zea NPV	-	-	Chen X.	Yes	AF334030.1	AF334030.1	AF334030.1	AF334030.1
Hemerocampa vetusta NPV	-	A24-5	Jehle J A.	No	AY706558.1	AY706623.1	-	AY706699.1
Hemileuca sp NPV	-	165	Rohrmann G F.	No	KC798397.1	-	-	-
Hemileuca sp NPV	-	-	Rohrmann G F.	Yes	KF158713.1	KF158713.1	KF158713.1	KF158713.1
Homona coffearia GV	745	-	Herniou E A/Thézé J.	No	MH454123	MH454163	MH458179	MH454203
Hoplodrina ambigua GV	-	M39-2	Lange M.	No	AY519232.1	AY519233.1	-	AY519231.1
Hyphantria cunea GV	-	A18-3_A5-1	Jehle J A.	No	AY706559.1	-	-	-
Hyphantria cunea GV	-	Hc1	Erbas Z.	No	KX156845.1	KX156846.1	-	KX156844.1
Hyphantria cunea NPV	-	S27	Jehle J A.	No	AY706560.1	AY706625.1	-	AY706700.1
Hyphantria cunea NPV	-	Tokyo	Croizier L.	No	-	-	-	D14573.1
Hyphantria cunea NPV	-	-	Alves C A.	Yes	AP009046.1	AP009046.1	AP009046.1	AP009046.1
Hyphantria cunea NPV	-	-	Lee H H.	No	-	-	-	AF300872.1
Hyposidra infixaria NPV	-	-	Antony B.	No	-	-	-	JF510036.1
Hyposidra talaca NPV	-	HytaNPV	Dasgupta S.	No	-	-	-	KP027542.1
Hyposidra talaca NPV	-	Terai_K1	Ghosh B.	No	-	-	-	KX665534.1
Hyposidra talaca NPV	-	-	Antony B.	No	-	-	-	JF510035.1
Idaea seriata NPV	402	-	Herniou E A/Thézé J.	No	MH454124	MH454164	-	MH454204
Iragoides fasciata NPV	-	Hangzhou	Yang L R.	No	-	-	-	FJ362523.1
Junonia coenia GV	19	-	Herniou E A/Thézé J.	No	MH454125	MH454165	-	MH454205
Junonia coenia NPV	-	M30-5	Lange M.	No	AY519235.1	AY519236.1	-	AY519234.1
Kotochalia junodi NPV	32	-	Herniou E A/Thézé J.	No	MH454126	MH454166	MH458180	MH454206
Lacanobia oleracea GV	a6	-	Herniou E A/Thézé J.	No	MH454127	MH454167	MH458181	MH454207
Lambdina fiscellaria NPV	-	GR15	Rohrmann G F.	Yes	KP752043.1	KP752043.1	KP752043.1	KP752043.1
Lasiocampa quercus NPV	202	-	Herniou E A/Thézé J.	No	MH454128	MH454168	MH458182	MH454208
Leucania separata NPV	AH1	-	Du E Q.	Yes	AY394490.1	AY394490.1	AY394490.1	AY394490.1
Leucania separata NPV	-	1	Wang J.	No	-	-	-	U30302.1
Leucania separata NPV	-	2	Wang J.	No	-	-	-	S83128.1
Leucoma salicis NPV	-	-	Jakubowska A K.	No	AY729809.1	-	AY729810.1	AY729808.1
Lonomia obliqua MNPV	-	SP_2000	Clara A S W.	Yes	KP763670.1	KP763670.1	KP763670.1	KP763670.1
Lonomia obliqua MNPV	-	-	Wolff J L C.	No	-	-	-	AF232690.2
Lymantria dispar MNPV	3029	-	Harrison R L.	Yes	KM386655.1	KM386655.1	KM386655.1	KM386655.1
Lymantria dispar MNPV	3041	-	Harrison R L.	Yes	KT626571.1	KT626571.1	KT626571.1	KT626571.1
Lymantria dispar MNPV	3054	-	Harrison R L.	Yes	KT626570.1	KT626570.1	KT626570.1	KT626570.1
Lymantria dispar MNPV	A21-MPV	-	Bischoff D S.	No	-	AF019970.1	-	-
Lymantria dispar MNPV	Ab-a624	-	Harrison R L.	Yes	KT626572.1	KT626572.1	KT626572.1	KT626572.1
Lymantria dispar MNPV	Chistoozernyi	-	Bakhvalov S A.	No	-	-	-	KJ685904.1
Lymantria dispar MNPV	Karasuk	-	Bakhvalov S A.	No	-	-	-	KJ685905.1
Lymantria dispar MNPV	Massachusetts	-	Podgwaite J D.	No	-	-	-	KJ685907.1
Lymantria dispar MNPV	New_Jersey	-	Podgwaite J D.	No	-	-	-	KJ685908.1
Lymantria dispar MNPV	New_York	-	Podgwaite J D.	No	-	-	-	KJ685909.1
Lymantria dispar MNPV	Tatarsk	-	Bakhvalov S A.	No	-	-	-	KJ685906.1
Lymantria dispar MNPV	-	1010	Harrison R L.	No	KF695055.1	-	-	-
Lymantria dispar MNPV	-	2161	Harrison R L.	Yes	KF695050.2	KF695050.2	KF695050.2	KF695050.2
Lymantria dispar MNPV	-	3057	Harrison R L.	No	KF695059.1	-	-	KF695051.1
Lymantria dispar MNPV	-	3058	Harrison R L.	No	KF695060.1	-	-	-
Lymantria dispar MNPV	-	3063	Harrison R L.	No	KF695061.1	-	-	-
Lymantria dispar MNPV	-	3065	Harrison R L.	No	KF695062.1	-	-	KF695052.1
Lymantria dispar MNPV	-	3152	Harrison R L.	No	KF695063.1	KF695066.1	-	KF695053.1
Lymantria dispar MNPV	-	A24-6	Jehle J A.	No	DQ235246.1	DQ235249.1	-	-
Lymantria dispar MNPV	-	BNP	Rabalski L.	Yes	KU377538.1	KU377538.1	KU377538.1	KU377538.1
Lymantria dispar MNPV	-	HrB	Harrison R L.	No	KF695064.1	-	-	KF695054.1
Lymantria dispar MNPV	-	Kashmir	Gani M.	No	KY296840.1	KY296841.1	-	KY296839.1
Lymantria dispar MNPV	-	LdMNPV-27_0	Kabilov M R.	Yes	KY249580.1	KY249580.1	KY249580.1	KY249580.1
Lymantria dispar MNPV	-	LdMNPV-27_2	Kabilov M R.	Yes	KP027546.2	KP027546.2	KP027546.2	KP027546.2
Lymantria dispar MNPV	-	LdMNPV-45_0	Martemyanov V V.	Yes	KU862282.1	KU862282.1	KU862282.1	KU862282.1
Lymantria dispar MNPV	-	Ld_Ninohe#1	Takatsuka J.	No	LC109268.1	LC109271.1	-	LC109265.1
Lymantria dispar MNPV	-	RR01	Krejmer-Rabalska M.	Yes	KX618634.1	KX618634.1	KX618634.1	KX618634.1
Lymantria dispar MNPV	-	T1	Gencer D.	No	KP939239.1	KP939240.1	-	KP939241.1
Lymantria dispar MNPV	-	T2	Gencer D.	No	KP939236.1	KP939237.1	-	KP939238.1
Lymantria dispar MNPV	-	T3	Gencer D.	No	KP939242.1	KP939243.1	-	KP939244.1
Lymantria dispar MNPV	-	T4	Gencer D.	No	KP939233.1	KP939234.1	-	KP939235.1
Lymantria dispar MNPV	-	-	An C.	No	-	-	-	AF499687.1
Lymantria dispar MNPV	-	-	Kuzio J.	Yes	AF081810.1	AF081810.1	AF081810.1	AF081810.1
Lymantria mathura mutiple NPV	-	Lyma_Kunohe_A#1	Takatsuka J.	No	LC109266.1	LC109269.1	-	LC109263.1
Lymantria mathura mutiple NPV	-	Lyma_Kunohe_B#1	Takatsuka J.	No	LC109267.1	LC109270.1	-	LC109264.1
Lymantria monacha NPV	-	463	Harrison R L.	No	KF695070.1	KF695071.1	-	KF695072.1
Lymantria monacha NPV	-	A14-3	Jehle J A.	No	AY706562.1	AY706627.1	-	AY706701.1
Lymantria monacha NPV	-	A19-3	Jehle J A.	No	AY706563.1	AY706628.1	-	AY706702.1
Lymantria xylina NPV	-	3061	Harrison R L.	No	KF695067.1	KF695068.1	-	KF695069.1
Lymantria xylina NPV	-	LyxyMNPV-5	Nai Y S.	Yes	GQ202541.1	GQ202541.1	GQ202541.1	GQ202541.1
Lymantria xylina NPV	-	S31	Jehle J A.	No	AY706564.1	AY706629.1	-	AY706703.1
Lymantria xylina NPV	-	-	Nai Y S.	No	FJ182055.1	FJ182056.1	-	-
Lymantria xylina NPV	-	-	Wu C Y.	No	-	-	-	AY842284.1
Lymantria xylina NPV 2	-	-	Nai Y S.	No	FJ182054.1	FJ182053.1	-	FJ182057.1
Mahasena corbetti NPV	692	-	Herniou E A/Thézé J.	No	MH454129	MH454169	-	MH454209
Malacosoma sp NPV	-	18	Rohrmann G F.	No	KC798392.1	-	-	-
Malacosoma americanum NPV	-	M39-4	Jehle J A.	No	AY706565.1	AY706630.1	-	AY706704.1
Malacosoma americanum NPV	-	-	Zeng F.	No	-	-	-	AY589504.1
Malacosoma californicum NPV	-	99	Cory J.	Yes	lef-8_MacoNPV_99	lef-9_MacoNPV_99	pif-2_MacoNPV_99	polyhedrin_MacoNPV_99
Malacosoma californicum NPV	-	M30-6	Lange M.	No	AY519238.1	AY519239.1	-	AY519237.1
Malacosoma disstria NPV	MdMNPV-A92	-	Erlandson M A.	No	-	-	-	U61732.1
Malacosoma neustria NPV	-	A2-6	Lange M.	No	AY519244.1	AY519245.1	-	AY519243.1
Malacosoma neustria NPV	-	S32	Jehle J A.	No	AY706569.1	AY706634.1	-	AY706708.1
Malacosoma neustria NPV	-	-	Dmitrenko V V.	No	-	-	-	X55658.1
Malacosoma neustria NPV	-	-	Jankevica L.	No	-	-	-	AJ277555.1
Malacosoma neustria NPV	-	-	Lange M.	No	-	-	-	AY127899.1
Malacosoma sp NPV	164	-	Rohrmann G F.	Yes	KU659594.1	KU659594.1	KU696418.1	KU707951.1
Malacosoma sp NPV	-	M28-2	Lange M.	No	AY519241.1	AY519242.1	-	AY519240.1
Mamestra brassicae MNPV	CHb1	-	Liu L.	Yes	JX138237.2	JX138237.2	JX138237.2	JX138237.2
Mamestra brassicae MNPV	K1	-	Choi J B.	Yes	JQ798165.1	JQ798165.1	JQ798165.1	JQ798165.1
Mamestra brassicae MNPV	Oxford	-	Cameron I R.	No	-	-	-	M20927.1
Mamestra brassicae MNPV	Tokyo	-	Mukawa S.	No	-	-	-	AB198073.1
Mamestra brassicae MNPV	-	A10-1	Jehle J A.	No	AY706566.1	AY706631.1	-	AY706705.1
Mamestra brassicae MNPV	-	A3-5	Jehle J A.	No	AY706567.1	AY706632.1	-	AY706706.1
Mamestra brassicae MNPV	-	CTa	Hou D.	Yes	KJ871680.1	KJ871680.1	KJ871680.1	KJ871680.1

Mamestra brassicae MNPV	-	S33	Jehle J A.	No	AY706568.1	AY706633.1	-	AY706707.1
Mamestra configurata NPV A	90_2	-	Li S.	Yes	U59461.2	U59461.2	U59461.2	U59461.2
Mamestra configurata NPV A	-	90_4	Li L.	Yes	AF539999.1	AF539999.1	AF539999.1	AF539999.1
Mamestra configurata NPV B	-	-	Li L.	Yes	AY126275.1	AY126275.1	AY126275.1	AY126275.1
Maruca vitrata NPV	-	-	Chen Y R.	Yes	EF125867.1	EF125867.1	EF125867.1	EF125867.1
Maruca vitrata NPV	-	-	Lee S.	No	-	-	-	DQ399596.1
Melanchra persicariae GV	26	-	Herniou E A/Thézé J.	No	MH454130	MH454170	MH458183	MH454210
Mocis latipes GV	-	Southern_Brazil	Ardisson-Araujo D M P.	Yes	KR011718.1	KR011718.1	KR011718.1	KR011718.1
Mythimna separata NPV	Geihoku	-	Kouassi L N.	No	AB308407.1	AB308408.1	-	AB308406.1
Mythimna unipuncta GV	-	KY410	Keathley C P.	No	JN701916.1	JN701911.1	-	JN701906.1
Mythimna unipuncta GV	-	MyunGV#8	Harrison R L.	Yes	KX855660.2	KX855660.2	KX855660.2	KX855660.2
Mythimna unipuncta NPV	#7	-	Harrison R L.	Yes	MF375894.1	MF375894.1	MF375894.1	MF375894.1
Mythimna unipuncta NPV	-	1411	Keathley C P.	No	JN701913.1	JN701908.1	-	JN701903.1
Mythimna unipuncta NPV	-	330	Keathley C P.	No	JN701912.1	JN701907.1	-	JN701902.1
Mythimna unipuncta NPV	-	KY310	Keathley C P.	No	JN701915.1	JN701910.1	-	JN701905.1
Mythimna unipuncta NPV	-	KY511	Keathley C P.	No	JN701914.1	JN701909.1	-	JN701904.1
Macroplectra nararia GV	254	-	Herniou E A/Thézé J.	No	AY449782.1	MH454171	AY449762.1	MH454211
Neodiprion abietis NPV	-	-	Duffy S P.	Yes	DQ317692.1	DQ317692.1	DQ317692.1	DQ317692.1
Neodiprion lecontei NPV	-	-	Lauzon H A.	Yes	AY349019.1	AY349019.1	AY349019.1	AY349019.1
Neodiprion sertifer NPV	-	-	Garcia-Maruniak A.	Yes	AY430810.1	AY430810.1	AY430810.1	AY430810.1
Neophasia sp NPV	-	11	Rohrmann G F.	No	KC798390.1	-	-	-
Nepytia phantasmaria NPV	-	A25-5	Jehle J A.	No	AY706571.1	AY706636.1	-	AY706709.1
Nymphalis io NPV	353	-	Herniou E A/Thézé J.	No	MH454131	MH454172	AY545990.1	MH454212
Operophtera bruceata NPV	-	ME	Broadley H J.	No	-	-	-	KY064007.1
Operophtera brumata NPV	-	OpbuNPV-MA	Harrison R L.	Yes	MF614691.1	MF614691.1	MF614691.1	MF614691.1
Orgyia antiqua NPV	a9	-	Herniou E A/Thézé J.	No	MH454132	MH454173	MH458184	MH454213
Orgyia mixta NPV	67	-	Herniou E A/Thézé J.	No	MH454133	MH454174	-	MH454214
Orgyia anartoides NPV	Oa-8	-	Bulach D M.	No	-	-	-	AF068188.1
Orgyia leucostigma NPV	-	CFS-77	Eveleigh R J M.	Yes	EU309041.1	EU309041.1	EU309041.1	EU309041.1
Orgyia pseudotsugata MNPV	-	-	Ahrens C H.	Yes	U75930.2	U75930.2	U75930.2	U75930.2
Orgyia pseudotsugata MNPV	-	-	Leisy D.	No	-	-	-	M14885.1
Orgyia pseudotsugata NPV	-	-	Jakubowska A.	No	AY895151.1	-	AY895152.1	AY895150.1
Oxyplax ochracea NPV	-	435	Wang J.	Yes	MF143631.1	MF143631.1	MF143631.1	MF143631.1
Pachypasa papyri NPV	436	-	Herniou E A/Thézé J.	No	MH454134	MH454175	MH458185	MH454215
Pandemis limitata GV	-	M36-1	Lange M.	No	AY519247.1	AY519248.1	-	AY519246.1
Panolis flammea NPV	i3	-	Herniou E A/Thézé J.	No	MH454135	MH454176	MH458186	MH454216
Peridroma morpontora GV	-	A25-3	Jehle J A.	No	AY706573.1	AY706638.1	-	AY706672.1
Peridroma sp NPV	-	167	Rohrmann G F.	No	KC798398.1	-	-	-
Peridroma sp NPV	-	GR_167	Rohrmann G F.	Yes	KM009991.1	KM009991.1	KM009991.1	KM009991.1
Peridroma margaritosa NPV	-	A25-4	Jehle J A.	No	AY706572.1	AY706637.1	-	AY706710.1
Perigonia lusca NPV	-	-	Ardisson-Araujo D M.	Yes	KM596836.1	KM596836.1	KM596836.1	KM596836.1
Perina nuda NPV	-	-	Chou C M.	No	-	-	-	U22824.1
Phalera bucephala NPV	204	-	Herniou E A/Thézé J.	No	-	MH454177	-	MH454217
Philosamia cynthia ricini NPV	-	-	Qian H.	Yes	JX404026.1	JX404026.1	JX404026.1	JX404026.1
Phryganidia californica NPV	-	M36-3	Lange M.	No	AY519250.1	AY519251.1	-	AY519249.1
Phthorimaea operculella GV	-	1	Jukes M D.	No	KF724712.1	-	-	-
Phthorimaea operculella GV	-	2	Jukes M D.	No	KU666536.1	-	-	-
Phthorimaea operculella GV	-	SA	Jukes M D.	Yes	-	KU666536.1	KU666536.1	KU666536.1
Phthorimaea operculella GV	-	-	Croizier L.	Yes	AF499596.1	AF499596.1	AF499596.1	AF499596.1
Pieris brassicae GV	-	S54	Jehle J A.	No	DQ235252.1	-	-	DQ235253.1
Pieris rapae GV	E3	Guangxi	Wen R.	Yes	GU111736.1	GU111736.1	GU111736.1	GU111736.1
Pieris rapae GV	-	M36-7	Lange M.	No	AY519253.1	AY519254.1	-	AY519252.1
Pieris rapae GV	-	S55	Jehle J A.	No	AY706575.1	AY706640.1	-	AY706673.1
Pieris rapae GV	-	-	Chen K.	No	-	-	-	AY428513.1
Pieris rapae GV	-	-	Oh S.	Yes	JX968491.1	JX968491.1	JX968491.1	JX968491.1
Plathypena scabra GV	-	A25-6	Jehle J A.	No	AY706579.1	AY706644.1	-	AY706675.1
Plodia interpunctella GV	-	Cambridge	Harrison R L.	Yes	KX151395.1	KX151395.1	KX151395.1	KX151395.1
Plusia acuta NPV	-	A14-5	Jehle J A.	No	AY706577.1	AY706642.1	-	AY706712.1
Plutella maculipennis NPV	-	A15-2	Jehle J A.	No	AY706578.1	AY706643.1	-	AY706713.1
Plutella xylostella GV	K1	-	Hashimoto Y.	Yes	AF270937.1	AF270937.1	AF270937.1	AF270937.1
Plutella xylostella GV	-	PLXYGV-SA001	Abdulkadir F.	No	-	-	-	KJ939451.1
Plutella xylostella GV	-	PlxyGV-SA001	Abdulkadir F.	No	-	KM359682.1	-	-
Plutella xylostella GV	-	PlxyGV-SA001	Adbulkadir F.	No	KM288415.1	-	-	-
Plutella xylostella GV	-	PxGV_C	Spence R J.	Yes	KU529791.1	KU529791.1	KU529791.1	KU529791.1
Plutella xylostella GV	-	PxGV_K	Spence R J.	Yes	KU529792.1	KU529792.1	KU529792.1	KU529792.1
Plutella xylostella GV	-	PxGV_M	Spence R J.	Yes	KU529793.1	KU529793.1	KU529793.1	KU529793.1
Plutella xylostella GV	-	PxGV_T	Spence R J.	Yes	KU529794.1	KU529794.1	KU529794.1	KU529794.1
Plutella xylostella GV	-	SA	Jukes M D.	Yes	KU666537.1	KU666537.1	KU666537.1	KU666537.1
Plutella xylostella MNPV	-	CL3	Harrison R L.	Yes	DQ457003.1	DQ457003.1	DQ457003.1	DQ457003.1
Polygonia c-album NPV	380	-	Herniou E A/Thézé J.	No	MH454136	MH454178	MH458187	MH454218
Pseudalatia unipuncta GV	Hawaii	-	Tanada Y.	No	AB290317.1	AB290318.1	-	AB290316.1
Pseudalatia unipuncta GV	Hawaiin	-	Li Y.	Yes	EU678671.1	EU678671.1	EU678671.1	EU678671.1
Pseudaletia sp GV	-	2	Rohrmann G F.	No	KC798388.1	-	-	-
Pseudaletia sp NPV	-	7	Rohrmann G F.	No	KC798389.1	-	-	-
Pseudoplusia includens NPV	-	458	Rowley D L.	No	HQ246015.1	HQ246016.1	-	HQ246017.1
Pseudoplusia includens NPV	-	Los_Angeles	Xu F.	No	-	-	-	EU401917.1
Pseudoplusia includens NPV	-	-	Xu F.	No	-	-	-	EU401916.1
Pseudoplusia includens NPV IE	-	-	Craveiro S R.	Yes	KJ631622.1	KJ631622.1	KJ631622.1	KJ631622.1
Pterolocera amplicornis NPV	-	M36-2	Lange M.	No	AY519256.1	AY519257.1	-	AY519255.1
Rachiplusia nu MNPV	-	-	Rodriguez V A.	No	-	-	-	DQ345451.1
Rachiplusia ou MNPV	RI	-	Harrison R L.	No	-	-	-	AF068270.1
Rachiplusia ou MNPV	-	1239	Rowley D L.	No	JN674777.1	JN674773.1	-	JN674769.1
Rachiplusia ou MNPV	-	2436	Rowley D L.	No	JN674778.1	JN674774.1	-	JN674770.1
Rachiplusia ou MNPV	-	3036	Rowley D L.	No	JN674779.1	JN674775.1	-	JN674771.1
Rachiplusia ou MNPV	-	-	Harrison R L.	Yes	AY145471.1	AY145471.1	AY145471.1	AY145471.1
Samia cynthia NPV	Nagano	-	Sasaki K.	Yes	LC375538.1	LC375538.1	LC375538.1	LC375538.1
Samia cynthia NPV	-	S36	Jehle J A.	No	AY706574.1	AY706639.1	-	AY706711.1
Samia ricini NPV	Guangxi	1	Sasaki K.	No	-	-	-	LC194892.1
Samia ricini NPV	Guangxi	2	Sasaki K.	No	-	-	-	LC194893.1
Samia ricini NPV	Guangxi	3	Sasaki K.	No	-	-	-	LC375541.1
Samia ricini NPV	Guangxi	-	Sasaki K.	Yes	LC375541.1	LC375541.1	LC375541.1	-
Samia ricini NPV	Mondulkiri	-	Sasaki K.	Yes	LC375542.1	LC375542.1	LC375542.1	LC375542.1
Samia ricini NPV	Son_La	-	Sasaki K.	Yes	LC375543.1	LC375543.1	LC375543.1	LC375543.1
Scotogramma trifolii GV	-	A26-3	Jehle J A.	No	AY706583.1	AY706648.1	-	AY706676.1
Spilarctia obliqua NPV	-	IISR-NPV-02	Senthil Kumar C M.	No	KT149869.1	KP172302.1	-	KP172301.1
Lemyra imparilis NPV	n2	-	Herniou E A/Thézé J.	No	MH454137	MH454179	-	MH454219
Spilosoma lutea GV	126	-	Herniou E A/Thézé J.	No	MH454138	MH454180	MH458188	MH454220
Spilosoma obliqua NPV	-	IIPR	Akram M.	Yes	KY550224.1	KY550224.1	KY550224.1	KY550224.1
Spilosoma obliqua NPV	-	IIPRnpv1	Akram M.	No	-	-	-	KT877650.1
Spilosoma phasma NPV	-	S3	Jehle J A.	No	AY706536.1	AY706601.1	-	AY706684.1
Spodoptera androgea GV	-	A25-7	Jehle J A.	No	DQ235248.1	DQ235247.1	-	-
Spodoptera exempta NPV	k11	-	Herniou E A/Thézé J.	No	MH454139	MH454181	-	MH454221
Spodoptera exempta NPV	-	var1	Graham R I.	No	-	-	-	JX488468.1
Spodoptera exigua MNPV	-	HT-SeG24	Theze J.	Yes	HG425346.1	HG425346.1	HG425346.1	HG425346.1
Spodoptera exigua MNPV	-	HT-SeG25	Theze J.	Yes	HG425347.2	HG425347.2	HG425347.2	HG425347.2
Spodoptera exigua MNPV	-	HT-SeG26	Theze J.	Yes	HG425348.1	HG425348.1	HG425348.1	HG425348.1
Spodoptera exigua MNPV	-	HT-SeSP2A	Theze J.	Yes	HG425349.2	HG425349.2	HG425349.2	HG425349.2
Spodoptera exigua MNPV	-	VT-SeAl1	Theze J.	Yes	HG425343.1	HG425343.1	HG425343.1	HG425343.1
Spodoptera exigua MNPV	-	VT-SeAl2	Theze J.	Yes	HG425344.1	HG425344.1	HG425344.1	HG425344.1
Spodoptera exigua MNPV	-	VT-SeOx4	Theze J.	Yes	HG425345.1	HG425345.1	HG425345.1	HG425345.1
Spodoptera exigua MNPV	-	YV	Darsouei R.	No	KT956220.1	-	-	KT956221.1
Spodoptera exigua MNPV	-	-	El-Dougdoug K A.	No	-	-	-	GQ392064.1
Spodoptera exigua MNPV	-	-	IJkel W F.	Yes	AF169823.1	AF169823.1	AF169823.1	AF169823.1
Spodoptera frugiperda GV	-	A12-4	Jehle J A.	No	AY706584.1	AY706649.1	-	AY706677.1
Spodoptera frugiperda GV	-	VG008	Cuartas P E.	Yes	KM371112.1	KM371112.1	KM371112.1	KM371112.1

Spodoptera frugiperda GV	-	VG014	Cuartas P.	No	KJ698694.1	KJ698692.1	-	KJ698696.1
Spodoptera frugiperda MNPV	-	1	Rowley D L.	No	GQ923694.1	-	-	GQ923749.1
Spodoptera frugiperda MNPV	-	1	Simon O.	Yes	HM595733.1	HM595733.1	HM595733.1	HM595733.1
Spodoptera frugiperda MNPV	-	19	Wolff J L.	Yes	EU258200.1	EU258200.1	EU258200.1	EU258200.1
Spodoptera frugiperda MNPV	-	2	Rowley D L.	No	GQ923695.1	-	-	GQ923750.1
Spodoptera frugiperda MNPV	-	2	Simon O.	Yes	JF899325.1	JF899325.1	JF899325.1	JF899325.1
Spodoptera frugiperda MNPV	-	2705	Rowley D L.	No	GQ923706.1	-	-	GQ923761.1
Spodoptera frugiperda MNPV	-	281	Rowley D L.	No	GQ923700.1	-	-	GQ923755.1
Spodoptera frugiperda MNPV	-	3	Rowley D L.	No	GQ923696.1	-	-	GQ923751.1
Spodoptera frugiperda MNPV	-	3146	Rowley D L.	No	GQ923707.1	-	-	GQ923762.1
Spodoptera frugiperda MNPV	-	3AP2	Harrison R L.	Yes	EF035042.2	EF035042.2	EF035042.2	EF035042.2
Spodoptera frugiperda MNPV	-	4	Rowley D L.	No	GQ923697.1	-	-	GQ923752.1
Spodoptera frugiperda MNPV	-	459	Rowley D L.	No	GQ923701.1	-	-	GQ923756.1
Spodoptera frugiperda MNPV	-	5	Rowley D L.	No	GQ923698.1	-	-	GQ923753.1
Spodoptera frugiperda MNPV	-	6	Rowley D L.	No	GQ923699.1	-	-	GQ923754.1
Spodoptera frugiperda MNPV	-	635	Rowley D L.	No	GQ923702.1	-	-	GQ923757.1
Spodoptera frugiperda MNPV	-	636	Rowley D L.	No	GQ923703.1	-	-	GQ923758.1
Spodoptera frugiperda MNPV	-	637	Rowley D L.	No	GQ923704.1	-	-	GQ923759.1
Spodoptera frugiperda MNPV	-	638	Rowley D L.	No	GQ923705.1	-	-	GQ923760.1
Spodoptera frugiperda MNPV	-	Colombian	Barrera G P.	Yes	KF891883.1	KF891883.1	KF891883.1	KF891883.1
Spodoptera frugiperda MNPV	-	Guasave	Escobedo-Bonilla C M.	No	-	-	-	KC845532.1
Spodoptera frugiperda MNPV	-	-	Gonzalez M A.	No	-	-	-	J04333.1
Spodoptera littoralis GV	66	-	Herniou E A/Thézé J.	No	AY449790.1	MH454182	AY449769.1	MH454222
Spodoptera littoralis MNPV	-	Az	Martins T.	No	-	-	-	AY600451.1
Spodoptera littoralis NPV	SlMNPV-M2	-	Croizier L.	No	-	-	-	D01017.1
Spodoptera littoralis NPV	Spli1	-	Takatsuka J.	No	-	-	-	LC109272.1
Spodoptera littoralis NPV	-	1213	Breitenbach J E.	No	JX454577.1	JX454592.1	-	JX454584.1
Spodoptera littoralis NPV	-	1263	Breitenbach J E.	No	JX454575.1	JX454598.1	-	JX454590.1
Spodoptera littoralis NPV	-	1628	Breitenbach J E.	No	JX454578.1	JX454593.1	-	JX454585.1
Spodoptera littoralis NPV	-	2424	Breitenbach J E.	No	JX454579.1	JX454594.1	-	JX454586.1
Spodoptera littoralis NPV	-	3003	Breitenbach J E.	No	JX454580.1	JX454595.1	-	JX454587.1
Spodoptera littoralis NPV	-	3017	Breitenbach J E.	No	JX454581.1	JX454596.1	-	JX454588.1
Spodoptera littoralis NPV	-	3032	Breitenbach J E.	No	JX454582.1	JX454597.1	-	JX454589.1
Spodoptera littoralis NPV	-	454	Breitenbach J E.	No	JX454576.1	JX454591.1	-	JX454583.1
Spodoptera littoralis NPV	-	A26-5	Jehle J A.	No	AY706585.1	AY706650.1	-	AY706717.1
Spodoptera littoralis NPV	-	A9-1	Jehle J A.	No	AY706586.1	AY706651.1	-	AY706718.1
Spodoptera littoralis NPV	-	AN1956	Breitenbach J E.	Yes	JX454574.1	JX454574.1	JX454574.1	JX454574.1
Spodoptera littoralis NPV	-	Egy-SLNPV	Ahmed Y E.	No	-	-	-	KY072799.1
Spodoptera littoralis NPV	-	SlMNPV-B_isolate_E15	Faktor O.	No	Y10669.1	-	-	-
Spodoptera littoralis NPV	-	-	Seufi A.	No	-	-	-	AY442260.1
Spodoptera litura GV	-	SlGV-K1	Wang Y.	Yes	DQ288858.2	DQ288858.2	DQ288858.2	DQ288858.2
Spodoptera litura MNPV	B-0-4	-	Zhu J.	No	-	-	FJ384666.1	-
Spodoptera litura MNPV	G10-3	-	Zhu J.	No	-	-	-	AY549964.1
Spodoptera litura MNPV	K-3	-	Zhu J.	No	-	-	-	AY552474.1
Spodoptera litura NPV	Bangalore	-	Jose J.	No	JF276035.1	-	-	-
Spodoptera litura NPV	E8	-	Bulach D M.	No	-	-	-	AF068189.1
Spodoptera litura NPV	G2	-	Pang Y.	Yes	AF325155.1	AF325155.1	AF325155.1	AF325155.1
Spodoptera litura NPV	GZ-1	-	Wei Y J.	No	-	-	-	AF037262.1
Spodoptera litura NPV	K1	-	Woo S.	No	-	-	-	DQ152923.1
Spodoptera litura NPV	Satsuma	-	Kouassi L N.	No	AB326103.1	AB326104.1	-	AB326102.1
Spodoptera litura NPV	Splt1	-	Takatsuka J.	No	-	-	-	LC109273.1
Spodoptera litura NPV	-	A17-3	Jehle J A.	No	AY706580.1	AY706645.1	-	AY706714.1
Spodoptera litura NPV	-	Act-1	Kouassi L N.	No	AB451187.1	-	-	-
Spodoptera litura NPV	-	Lab-1	Kouassi L N.	No	AB451186.1	-	-	-
Spodoptera litura NPV	-	Os-7	Kouassi L N.	No	AB451185.1	-	-	-
Spodoptera litura NPV	-	S37	Jehle J A.	No	AY706581.1	AY706646.1	-	AY706715.1
Spodoptera litura NPV	-	SlNPV-K2	Wang Y.	No	-	-	-	DQ350142.1
Spodoptera litura NPV	-	-	Kouassi L N.	No	AB583682.1	AB458508.1	-	-
Spodoptera litura NPV	-	-	Li C.	No	-	-	AF216302.1	-
Spodoptera litura NPV II	-	-	Li Y.	Yes	EU780426.1	EU780426.1	EU780426.1	EU780426.1
Spodoptera albula NPV	k7	-	Herniou E A/Thézé J.	No	MH454140	MH454183	-	MH454223
Spodoptera terricola NPV	-	A26-1	Jehle J A.	No	AY706582.1	AY706647.1	-	AY706716.1
Sucra jujuba NPV	-	473	Liu X.	Yes	KJ676450.1	KJ676450.1	KJ676450.1	KJ676450.1
Thysanoplusia orichalcea NPV	-	A28-1	Jehle J A.	No	AY706587.1	AY706652.1	-	AY706719.1
Thysanoplusia orichalcea NPV	-	-	Cheng X W.	No	-	-	-	AF169480.1
Thysanoplusia orichalcea NPV	-	-	Wang L.	No	-	EU153368.1	-	-
Thysanoplusia orichalcea NPV	-	p2	Wang Y S.	Yes	JX467702.1	JX467702.1	JX467702.1	JX467702.1
Tineola bisselliella NPV	-	M50-4	Jehle J A.	No	AY706588.1	AY706653.1	-	AY706720.1
Trabala vishnou NPV	195	-	Herniou E A/Thézé J.	No	MH454141	-	-	MH454224
Trichoplusia ni GV	-	M10-5	Lange M.	No	AY519202.1	AY519203.1	-	AY519201.1
Trichoplusia ni GV	-	-	Akiyoshi D.	No	-	-	-	K02910.1
Trichoplusia ni GV LBIV-12	-	LBIV-12	Del Rincon-Castro M C.	Yes	KU752557.1	KU752557.1	KU752557.1	KU752557.1
Trichoplusia ni NPV	-	1004	Rowley D L.	No	JN674759.1	JN674735.1	-	JN674711.1
Trichoplusia ni NPV	-	1141	Rowley D L.	No	JN674760.1	JN674736.1	-	JN674712.1
Trichoplusia ni NPV	-	1185	Rowley D L.	No	JN674761.1	JN674737.1	-	JN674713.1
Trichoplusia ni NPV	-	1237	Rowley D L.	No	JN674762.1	JN674738.1	-	JN674714.1
Trichoplusia ni NPV	-	1241	Rowley D L.	No	JN674763.1	JN674739.1	-	JN674715.1
Trichoplusia ni NPV	-	207	Rowley D L.	No	JN674745.1	JN674721.1	-	JN674697.1
Trichoplusia ni NPV	-	209	Rowley D L.	No	JN674746.1	JN674722.1	-	JN674698.1
Trichoplusia ni NPV	-	227	Rowley D L.	No	JN674747.1	JN674723.1	-	JN674699.1
Trichoplusia ni NPV	-	230	Rowley D L.	No	JN674748.1	JN674724.1	-	JN674700.1
Trichoplusia ni NPV	-	239	Rowley D L.	No	JN674749.1	JN674725.1	-	JN674701.1
Trichoplusia ni NPV	-	242	Rowley D L.	No	JN674750.1	JN674726.1	-	JN674702.1
Trichoplusia ni NPV	-	246	Rowley D L.	No	JN674751.1	JN674727.1	-	JN674703.1
Trichoplusia ni NPV	-	252	Rowley D L.	No	JN674752.1	JN674728.1	-	JN674704.1
Trichoplusia ni NPV	-	2541	Rowley D L.	No	JN674764.1	JN674740.1	-	JN674716.1
Trichoplusia ni NPV	-	270	Rowley D L.	No	JN674753.1	JN674729.1	-	JN674705.1
Trichoplusia ni NPV	-	2700	Rowley D L.	No	JN674765.1	JN674741.1	-	JN674717.1
Trichoplusia ni NPV	-	2703	Rowley D L.	No	JN674766.1	JN674742.1	-	JN674718.1
Trichoplusia ni NPV	-	271	Rowley D L.	No	JN674754.1	JN674730.1	-	JN674706.1
Trichoplusia ni NPV	-	282	Rowley D L.	No	JN674755.1	JN674731.1	-	JN674707.1
Trichoplusia ni NPV	-	3073	Rowley D L.	No	JN674767.1	JN674743.1	-	JN674719.1
Trichoplusia ni NPV	-	3091	Rowley D L.	No	JN674768.1	JN674744.1	-	JN674720.1
Trichoplusia ni NPV	-	397	Rowley D L.	No	JN674756.1	JN674732.1	-	JN674708.1
Trichoplusia ni NPV	-	399	Rowley D L.	No	JN674757.1	JN674733.1	-	JN674709.1
Trichoplusia ni NPV	-	455	Rowley D L.	No	JN674758.1	JN674734.1	-	JN674710.1
Trichoplusia ni NPV	-	-	Abrahams R.	No	-	AY542547.1	-	-
Trichoplusia ni NPV	-	-	Fielding B C.	No	-	-	-	AF093405.1
Trichoplusia ni NPV	-	-	Willis L G.	Yes	DQ017380.1	DQ017380.1	DQ017380.1	DQ017380.1
Trichoplusia orichalcea NPV	b9	-	Herniou E A/Thézé J.	No	MH454142	-	-	MH454225
Urbanus proteus NPV	-	Southern_Brazil	Santos E R.	Yes	KR011717.2	KR011717.2	KR011717.2	KR011717.2
Utetheisa pulchella NPV	142	-	Herniou E A/Thézé J.	No	MH454143	-	-	MH454226
Vanessa atalanta NPV	54	-	Herniou E A/Thézé J.	No	MH454144	MH454184	MH458189	MH454227
Vanessa cardui NPV	367	-	Herniou E A/Thézé J.	No	MH454145	MH454185	MH458190	MH454228
Wiseana cervinata GV	342	-	Herniou E A/Thézé J.	No	MH454146	-	MH458191	MH454229
Wiseana cervinata NPV	344	-	Herniou E A/Thézé J.	No	AY449784.1	-	AY449764.1	MH454230
Wiseana signata NPV	WisiSNPV	-	Sadler T J.	No	-	-	-	AF016916.1
Xestia c-nigrum GV	alpha-4	-	Goto C.	No	U70914.1	U70913.1	U70891.1	U70069.1
Xestia c-nigrum GV	-	-	Hayakawa T.	Yes	AF162221.1	AF162221.1	AF162221.1	AF162221.1

Table S2. Host ecology database

Order	Superfamily	Family	Subfamily	Species	Pest?	Ecozone	Host plant growth
Diptera	Culicidae	Culicidae	Culicinae	*Culex nigripalpus*	Yes	Neotropical	Detritophagous
Hymenoptera	Tenthredinoidea	Diprionidae	Diprioninae	*Neodiprion abietis*	Yes	Nearctic	Woody
Hymenoptera	Tenthredinoidea	Diprionidae	Diprioninae	*Neodiprion lecontei*	Yes	Nearctic	Woody
Hymenoptera	Tenthredinoidea	Diprionidae	Diprioninae	*Neodiprion sertifer*	Yes	Palearctic	Woody
Lepidoptera	Bombycoidea	Saturniidae	Saturniinae	*Actias selene*		Indo-Malaya	Woody
Lepidoptera	Bombycoidea	Saturniidae	Saturniinae	*Antheraea pernyi*		Cosmopolitan	Woody
Lepidoptera	Bombycoidea	Saturniidae	Saturniinae	*Antheraea proylei*		Indo-Malaya	Woody
Lepidoptera	Bombycoidea	Saturniidae	Saturniinae	*Antheraea yamamai*		Palearctic	Woody
Lepidoptera	Bombycoidea	Saturniidae	Saturniinae	*Samia cynthia*		Cosmopolitan	Woody
Lepidoptera	Bombycoidea	Saturniidae	Hemileucinae	*Coloradia pandora*		Nearctic	Woody
Lepidoptera	Bombycoidea	Saturniidae	Hemileucinae	*Dirphia peruvianus*		Neotropical	Woody
Lepidoptera	Bombycoidea	Saturniidae	Hemileucinae	*Hemileuca sp*		Nearctic	Woody
Lepidoptera	Bombycoidea	Saturniidae	Hemileucinae	*Lonomia obliqua*		Neotropical	Woody
Lepidoptera	Bombycoidea	Bombycidae	Endromidae	*Andraca bipunctata*	Yes	Indo-Malaya	Woody
Lepidoptera	Bombycoidea	Bombycidae	Bombycinae	*Bombyx mandarina*		Palearctic	Woody
Lepidoptera	Bombycoidea	Bombycidae	Bombycinae	*Bombyx mori*		Cosmopolitan	Woody
Lepidoptera	Bombycoidea	Sphingidae	Smerinthinae	*Clanis bilineata*		Cosmopolitan	Woody
Lepidoptera	Bombycoidea	Sphingidae	Macroglossinae	*Erinnyis ello*		Americas	Herb/Woody
Lepidoptera	Bombycoidea	Sphingidae	Macroglossinae	*Perigonia lusca*		Neotropical	Woody
Lepidoptera	Bombycoidea	Anthelidae	Anthelinae	*Pterolocera amplicornis*		Australasia	Herbaceous
Lepidoptera	Choreutoidea	Choreutidae	Choreutinae	*Anthophila fabriciana*		Palearctic	Herbaceous
Lepidoptera	Gelechioidea	Gelechiidae	Gelechiinae	*Phthorimaea operculella*	Yes	Cosmopolitan	Herbaceous
Lepidoptera	Geometroidea	Geometridae	Ennominae	*Abraxas_grossulariata*		Palearctic	Woody
Lepidoptera	Geometroidea	Geometridae	Ennominae	*Apocheima cinerarium*	Yes	Palearctic	Woody
Lepidoptera	Geometroidea	Geometridae	Ennominae	*Boarmia bistortata*		Palearctic	Woody
Lepidoptera	Geometroidea	Geometridae	Ennominae	*Buzura suppressaria*		Indo-Malaya	Woody
Lepidoptera	Geometroidea	Geometridae	Ennominae	*Ectropis grisescens*		Indo-Malaya	Woody
Lepidoptera	Geometroidea	Geometridae	Ennominae	*Ectropis obliqua*		Indo-Malaya	Woody
Lepidoptera	Geometroidea	Geometridae	Ennominae	*Erannis defoliaria*		Palearctic	Woody
Lepidoptera	Geometroidea	Geometridae	Ennominae	*Hyposidra infixaria*		Indo-Malaya	Woody
Lepidoptera	Geometroidea	Geometridae	Ennominae	*Hyposidra talaca*		Indo-Malaya	Woody
Lepidoptera	Geometroidea	Geometridae	Ennominae	*Lambdina fiscellaria*		Nearctic	Woody
Lepidoptera	Geometroidea	Geometridae	Ennominae	*Nepytia phantasmaria*		Nearctic	Woody
Lepidoptera	Geometroidea	Geometridae	Ennominae	*Sucra jujuba*	Yes	Indo-Malaya	Woody
Lepidoptera	Geometroidea	Geometridae	Sterrhinae	*Idaea seriata*		Palearctic	Woody
Lepidoptera	Geometroidea	Geometridae	Larentiinae	*Operophtera bruceata*	Yes	Nearctic	Woody
Lepidoptera	Geometroidea	Geometridae	Larentiinae	*Operophtera brumata*	Yes	Holarctic	Woody
Lepidoptera	Gracillarioidea	Gracillariidae	Gracillariinae	*Caloptilia theivora*	Yes	Indo-Malaya	Woody
Lepidoptera	Hepialoidea	Hepialidae	Hepialidae	*Wiseana cervinata*	Yes	Australasia	Herbaceous
Lepidoptera	Hepialoidea	Hepialidae	Hepialidae	*Wiseana signata*		Australasia	Herbaceous
Lepidoptera	Lasiocampoidea	Lasiocampidae	Pinarinae	*Cyclophragma undans*		Palearctic	Woody
Lepidoptera	Lasiocampoidea	Lasiocampidae	Lasiocampinae	*Dendrolimus kikuchii*		Indo-Malaya	Woody
Lepidoptera	Lasiocampoidea	Lasiocampidae	Lasiocampinae	*Lasiocampa quercus*		Palearctic	Woody
Lepidoptera	Lasiocampoidea	Lasiocampidae	Malacosomatinae	*Malacosoma americanum*		Nearctic	Woody
Lepidoptera	Lasiocampoidea	Lasiocampidae	Malacosomatinae	*Malacosoma californicum*		Nearctic	Woody
Lepidoptera	Lasiocampoidea	Lasiocampidae	Malacosomatinae	*Malacosoma disstria*		Nearctic	Woody
Lepidoptera	Lasiocampoidea	Lasiocampidae	Malacosomatinae	*Malacosoma neustria*		Palearctic	Woody
Lepidoptera	Lasiocampoidea	Lasiocampidae	Pinarinae	*Pachypasa papyri*	Yes	Afrotropical	Woody
Lepidoptera	Lasiocampoidea	Lasiocampidae	Lasiocampinae	*Trabala vishnou*	Yes	Indo-Malaya	Woody
Lepidoptera	Noctuoidea	Erebidae	Erebinae	*Achaea faber*	Yes	Afrotropical	Woody
Lepidoptera	Noctuoidea	Erebidae	Erebinae	*Achaea janata*	Yes	Indo-Malaya	Herb/Woody
Lepidoptera	Noctuoidea	Noctuidae	Noctuinae	*Agrotis exclamationis*	Yes	Palearctic	Herbaceous
Lepidoptera	Noctuoidea	Noctuidae	Noctuinae	*Agrotis ipsilon*	Yes	Cosmopolitan	Herbaceous
Lepidoptera	Noctuoidea	Noctuidae	Noctuinae	*Agrotis segetum*	Yes	Palearctic	Herbaceous
Lepidoptera	Noctuoidea	Erebidae	Arctiinae	*Amsacta albistriga*		Indo-Malaya	Herbaceous
Lepidoptera	Noctuoidea	Noctuidae	Plusiinae	*Anagrapha falcifera*	Yes	Nearctic	Herbaceous
Lepidoptera	Noctuoidea	Noctuidae	Scoliopteryginae	*Anomis sabulifera*	Yes	Paleotropical	Woody
Lepidoptera	Noctuoidea	Noctuidae	Eulepidotinae	*Anticarsia gemmatalis*	Yes	Americas	Herbaceous
Lepidoptera	Noctuoidea	Erebidae	Catocalinae	*Aroa discalis*		Afrotropical	Herbaceous
Lepidoptera	Noctuoidea	Noctuidae	Plusiinae	*Autographa biloba*	Yes	Nearctic	Herbaceous
Lepidoptera	Noctuoidea	Noctuidae	Plusiinae	*Autographa californica*	Yes	Nearctic	Herbaceous
Lepidoptera	Noctuoidea	Noctuidae	Plusiinae	*Autographa gamma*	Yes	Cosmopolitan	Herbaceous
Lepidoptera	Noctuoidea	Noctuidae	Plusiinae	*Autographa nigrisigna*	Yes	Indo-Malaya	Herbaceous
Lepidoptera	Noctuoidea	Noctuidae	Amphipyrinae	*Busseola fusca*	Yes	Afrotropical	Herbaceous
Lepidoptera	Noctuoidea	Noctuidae	Noctuinae	*Cerapteryx graminis*		Holarctic	Herbaceous
Lepidoptera	Noctuoidea	Noctuidae	Plusiinae	*Chrysodeixis chalcites*	Yes	Cosmopolitan	Herbaceous
Lepidoptera	Noctuoidea	Notodontidae	Pygaerinae	*Clostera anachoreta*		Palearctic	Woody
Lepidoptera	Noctuoidea	Notodontidae	Pygaerinae	*Clostera anastomosis*		Palearctic	Woody
Lepidoptera	Noctuoidea	Notodontidae	Thaumetopoeinae	*Cynosarga chrysolopa*		Australasia	Woody
Lepidoptera	Noctuoidea	Limacodidae	Limacodinae	*Darna trima*		Indo-Malaya	Woody
Lepidoptera	Noctuoidea	Erebidae	Lymantriinae	*Dasychira plagiata*		Nearctic	Woody
Lepidoptera	Noctuoidea	Erebidae	Lymantriinae	*Dasychira pudibunda*		Palearctic	Woody
Lepidoptera	Noctuoidea	Erebidae	Arctiinae	*Estigmene acrea*		Nearctic	Herb/Woody
Lepidoptera	Noctuoidea	Noctuidae	Noctuinae	*Euplexia lucipara*		Palearctic	Herbaceous
Lepidoptera	Noctuoidea	Erebidae	Lymantriinae	*Euproctis chrysorrhoea*		Holarctic	Woody
Lepidoptera	Noctuoidea	Erebidae	Lymantriinae	*Euproctis digramma*		Indo-Malaya	Woody
Lepidoptera	Noctuoidea	Erebidae	Lymantriinae	*Euproctis pseudoconspersa*		Palearctic	Woody
Lepidoptera	Noctuoidea	Erebidae	Lymantriinae	*Euproctis similis*		Nearctic	Woody

Lepidoptera	Noctuoidea	Erebidae	Lymantriinae	*Hemerocampa vetusta*		Nearctic	Woody
Lepidoptera	Noctuoidea	Erebidae	Arctiinae	*Hyphantria cunea*		Holarctic	Woody
Lepidoptera	Noctuoidea	Erebidae	Arctiinae	*Lemyra imparilis*		Palearctic	Woody
Lepidoptera	Noctuoidea	Erebidae	Lymantriinae	*Leucoma salicis*		Holarctic	Woody
Lepidoptera	Noctuoidea	Erebidae	Lymantriinae	*Lymantria dispar*	Yes	Holarctic	Woody
Lepidoptera	Noctuoidea	Erebidae	Lymantriinae	*Lymantria mathura*		Palearctic	Woody
Lepidoptera	Noctuoidea	Erebidae	Lymantriinae	*Lymantria monacha*		Palearctic	Woody
Lepidoptera	Noctuoidea	Erebidae	Lymantriinae	*Lymantria xylina*		Indo-Malaya	Woody
Lepidoptera	Noctuoidea	Erebidae	Lymantriinae	*Orgyia anartoides*	Yes	Australasia	Woody
Lepidoptera	Noctuoidea	Erebidae	Lymantriinae	*Orgyia antiqua*	Yes	Cosmopolitan	Woody
Lepidoptera	Noctuoidea	Erebidae	Lymantriinae	*Orgyia leucostigma*	Yes	Nearctic	Woody
Lepidoptera	Noctuoidea	Erebidae	Lymantriinae	*Orgyia mixta*	Yes	Afrotropical	Woody
Lepidoptera	Noctuoidea	Erebidae	Lymantriinae	*Orgyia pseudotsugata*	Yes	Nearctic	Woody
Lepidoptera	Noctuoidea	Erebidae	Lymantriinae	*Perina nuda*	Yes	Indo-Malaya	Woody
Lepidoptera	Noctuoidea	Erebidae	Arctiinae	*Pareuchaetes pseudoinsulata*		Pantropical	Woody
Lepidoptera	Noctuoidea	Erebidae	Arctiinae	*Spilosoma lutea*		Palearctic	Herb/Woody
Lepidoptera	Noctuoidea	Erebidae	Arctiinae	*Spilosoma obliqua*		Indo-Malaya	Herb/Woody
Lepidoptera	Noctuoidea	Erebidae	Arctiinae	*Spilosoma phasma*		Palearctic	Woody
Lepidoptera	Noctuoidea	Erebidae	Arctiinae	*Utetheisa pulchella*		Paleotropical	Herbaceous
Lepidoptera	Noctuoidea	Noctuidae	Hadeninae	*Mocis latipes*		Neotropical	Herbaceous
Lepidoptera	Noctuoidea	Noctuidae	Hadeninae	*Pseudaletia sp*	Yes	Cosmopolitan	Herbaceous
Lepidoptera	Noctuoidea	Noctuidae	Hadeninae	*Mythimna unipuncta*	Yes	Cosmopolitan	Herbaceous
Lepidoptera	Noctuoidea	Noctuidae	Heliothinae	*Helicoverpa armigera*	Yes	Cosmopolitan	Herbaceous
Lepidoptera	Noctuoidea	Noctuidae	Heliothinae	*Helicoverpa assulta*		Pantropical	Herbaceous
Lepidoptera	Noctuoidea	Noctuidae	Heliothinae	*Helicoverpa gelotopoeon*		Neotropica	Herbaceous
Lepidoptera	Noctuoidea	Noctuidae	Heliothinae	*Helicoverpa zea*	Yes	Cosmopolitan	Herbaceous
Lepidoptera	Noctuoidea	Noctuidae	Hypeninae	*Hypena scabra*	Yes	Nearctic	Herb/Woody
Lepidoptera	Noctuoidea	Noctuidae	Noctuinae	*Euxoa ochrogaster*		Holarctic	Herbaceous
Lepidoptera	Noctuoidea	Noctuidae	Noctuinae	*Hoplodrina ambigua*		Palearctic	Herbaceous
Lepidoptera	Noctuoidea	Noctuidae	Noctuinae	*Lacanobia oleracea*		Palearctic	Herbaceous
Lepidoptera	Noctuoidea	Noctuidae	Noctuinae	*Leucania separata*	Yes	Indo-Malaya	Herbaceous
Lepidoptera	Noctuoidea	Noctuidae	Noctuinae	*Mamestra brassicae*	Yes	Palearctic	Herbaceous
Lepidoptera	Noctuoidea	Noctuidae	Noctuinae	*Mamestra configurata*	Yes	Nearctic	Herbaceous
Lepidoptera	Noctuoidea	Noctuidae	Noctuinae	*Melanchra persicariae*		Palearctic	Woody
Lepidoptera	Noctuoidea	Noctuidae	Noctuinae	*Mythimna separata*	Yes	Indo-Malaya	Herbaceous
Lepidoptera	Noctuoidea	Noctuidae	Noctuinae	*Mythimna unipuncta*	Yes	Cosmopolitan	Herbaceous
Lepidoptera	Noctuoidea	Noctuidae	Noctuinae	*Panolis flammea*	Yes	Palearctic	Woody
Lepidoptera	Noctuoidea	Noctuidae	Noctuinae	*Peridroma margaritosa*	Yes	Cosmopolitan	Herb/Woody
Lepidoptera	Noctuoidea	Noctuidae	Noctuinae	*Peridroma morpontora*	Yes	Indo-Malaya	Woody
Lepidoptera	Noctuoidea	Noctuidae	Noctuinae	*Scotogramma trifolii*	Yes	Cosmopolitan	Herb/Woody
Lepidoptera	Noctuoidea	Noctuidae	Noctuinae	*Spodoptera albula*	Yes	Neotropical	Herbaceous
Lepidoptera	Noctuoidea	Noctuidae	Noctuinae	*Spodoptera androgea*	Yes	Neotropical	Herbaceous
Lepidoptera	Noctuoidea	Noctuidae	Noctuinae	*Spodoptera exempta*	Yes	Pantropical	Herbaceous
Lepidoptera	Noctuoidea	Noctuidae	Noctuinae	*Spodoptera exigua*	Yes	Cosmopolitan	Herbaceous
Lepidoptera	Noctuoidea	Noctuidae	Noctuinae	*Spodoptera frugiperda*	Yes	Cosmopolitan	Herbaceous
Lepidoptera	Noctuoidea	Noctuidae	Noctuinae	*Spodoptera littoralis*	Yes	Afrotropical	Herbaceous
Lepidoptera	Noctuoidea	Noctuidae	Noctuinae	*Spodoptera litura*	Yes	Cosmopolitan	Herbaceous
Lepidoptera	Noctuoidea	Noctuidae	Noctuinae	*Spodoptera terricola*	Yes	Cosmopolitan	Herbaceous
Lepidoptera	Noctuoidea	Noctuidae	Noctuinae	*Xestia c-nigrum*	Yes	Holarctic	Herbaceous
Lepidoptera	Noctuoidea	Noctuidae	Plusiinae	*Plusia acuta*	Yes	Pantropical	Herbaceous
Lepidoptera	Noctuoidea	Noctuidae	Plusiinae	*Pseudoplusia includens*	Yes	Nearctic	Herbaceous
Lepidoptera	Noctuoidea	Noctuidae	Plusiinae	*Rachiplusia nu*	Yes	Neotropical	Herbaceous
Lepidoptera	Noctuoidea	Noctuidae	Plusiinae	*Rachiplusia ou*	Yes	Nearctic	Herbaceous
Lepidoptera	Noctuoidea	Noctuidae	Plusiinae	*Thysanoplusia orichalcea*	Yes	Cosmopolitan	Herbaceous
Lepidoptera	Noctuoidea	Noctuidae	Plusiinae	*Trichoplusia ni*	Yes	Cosmopolitan	Herbaceous
Lepidoptera	Noctuoidea	Noctuidae	Plusiinae	*Trichoplusia orichalcea*	Yes	Pantropical	Herbaceous
Lepidoptera	Noctuoidea	Notodontidae	Phalerinae	*Phalera bucephala*		Palearctic	Woody
Lepidoptera	Noctuoidea	Notodontidae	Dioptinae	*Phryganidia californica*	Yes	Nearctic	Woody
Lepidoptera	Papilionoidea	Nymphalidae	Heliconiinae	*Agraulis vanillae*		Americas	Herbaceous
Lepidoptera	Papilionoidea	Nymphalidae	Heliconiinae	*Dione juno*		Neotropical	Herbaceous
Lepidoptera	Papilionoidea	Nymphalidae	Heliconiinae	*Heliconius erato*		Neotropical	Herbaceous
Lepidoptera	Papilionoidea	Nymphalidae	Nymphalinae	*Aglais urticae*		Palearctic	Herbaceous
Lepidoptera	Papilionoidea	Nymphalidae	Nymphalinae	*Junonia coenia*		Nearctic	Herbaceous
Lepidoptera	Papilionoidea	Nymphalidae	Nymphalinae	*Nymphalis io*		Palearctic	Herbaceous
Lepidoptera	Papilionoidea	Nymphalidae	Nymphalinae	*Polygonia c-album*		Palearctic	Woody
Lepidoptera	Papilionoidea	Nymphalidae	Nymphalinae	*Vanessa atalanta*		Cosmopolitan	Herbaceous
Lepidoptera	Papilionoidea	Nymphalidae	Nymphalinae	*Vanessa cardui*		Cosmopolitan	Herbaceous
Lepidoptera	Papilionoidea	Pieridae	Pierinae	*Aporia crataegi*		Palearctic	Woody
Lepidoptera	Papilionoidea	Pieridae	Pierinae	*Neophasia sp*		Nearctic	Woody
Lepidoptera	Papilionoidea	Pieridae	Pierinae	*Pieris brassicae*	Yes	Cosmopolitan	Herbaceous
Lepidoptera	Papilionoidea	Pieridae	Pierinae	*Pieris rapae*	Yes	Cosmopolitan	Herbaceous
Lepidoptera	Papilionoidea	Pieridae	Coliadinae	*Catopsilia pomona*		Indo-Malaya	Woody
Lepidoptera	Papilionoidea	Hesperiidae	Pyrginae	*Urbanus proteus*	Yes	Neotropical	Herbaceous
Lepidoptera	Pyraloidea	Crambidae	Pyraustinae	*Cnaphalocrocis medinalis*		Indo-Malaya	Herbaceous
Lepidoptera	Pyraloidea	Crambidae	Spilomelinae	*Condylorrhiza vestigialis*		Americas	Woody
Lepidoptera	Pyraloidea	Crambidae	Spilomelinae	*Diaphania pulverulentalis*		Indo-Malaya	Woody
Lepidoptera	Pyraloidea	Crambidae	Spilomelinae	*Maruca vitrata*		Pantropical	Woody
Lepidoptera	Pyraloidea	Crambidae	Crambinae	*Diatraea saccharalis*	Yes	Neotropical	Herbaceous
Lepidoptera	Pyraloidea	Pyralidae	Phycitinae	*Cadra cautella*		Cosmopolitan	Woody
Lepidoptera	Pyraloidea	Pyralidae	Phycitinae	*Plodia interpunctella*	Yes	Cosmopolitan	Herb/Woody
Lepidoptera	Pyraloidea	Pyralidae	Galleriinae	*Galleria mellonella*	Yes	Cosmopolitan	Detritophagous
Lepidoptera	Tineoidea	Psychidae	Psychidae	*Kotochalia junodi*		Afrotropical	Woody

Lepidoptera	Tineoidea	Psychidae	Psychidae	*Mahasena corbetti*		Indo-Malaya	Woody
Lepidoptera	Tineoidea	Tineidae	Tineinae	*Tineola bisselliella*	Yes	Cosmopolitan	Detritophagous
Lepidoptera	Tortricoidea	Tortricidae	Olethreutinae	*Cryptophlebia leucotreta*		Paleotropical	Woody
Lepidoptera	Tortricoidea	Tortricidae	Olethreutinae	*Cydia pomonella*	Yes	Cosmopolitan	Woody
Lepidoptera	Tortricoidea	Tortricidae	Olethreutinae	*Epinotia aporema*	Yes	Neotropical	Woody
Lepidoptera	Tortricoidea	Tortricidae	Olethreutinae	*Epinotia granitalis*		Palearctic	Woody
Lepidoptera	Tortricoidea	Tortricidae	Tortricinae	*Adoxophyes honmai*	Yes	Palearctic	Woody
Lepidoptera	Tortricoidea	Tortricidae	Tortricinae	*Adoxophyes orana*	Yes	Palearctic	Woody
Lepidoptera	Tortricoidea	Tortricidae	Tortricinae	*Amelia pallorana*	Yes	Nearctic	Herbaceous
Lepidoptera	Tortricoidea	Tortricidae	Tortricinae	*Amorbia cuneacapsa*	Yes	Nearctic	Woody
Lepidoptera	Tortricoidea	Tortricidae	Tortricinae	*Archips cerasivoranus*	Yes	Nearctic	Woody
Lepidoptera	Tortricoidea	Tortricidae	Tortricinae	*Archips rosanus*	Yes	Holarctic	Woody
Lepidoptera	Tortricoidea	Tortricidae	Tortricinae	*Choristoneura fumiferana*	Yes	Nearctic	Woody
Lepidoptera	Tortricoidea	Tortricidae	Tortricinae	*Choristoneura murinana*		Holarctic	Woody
Lepidoptera	Tortricoidea	Tortricidae	Tortricinae	*Choristoneura occidentalis*	Yes	Nearctic	Woody
Lepidoptera	Tortricoidea	Tortricidae	Tortricinae	*Choristoneura rosaceana*		Cosmopolitan	Woody
Lepidoptera	Tortricoidea	Tortricidae	Tortricinae	*Choristoneura viridis*		Nearctic	Woody
Lepidoptera	Tortricoidea	Tortricidae	Tortricinae	*Cnephasia longana*		Cosmopolitan	Herbaceous
Lepidoptera	Tortricoidea	Tortricidae	Tortricinae	*Epiphyas postvittana*		Australasia	Herb/Woody
Lepidoptera	Tortricoidea	Tortricidae	Tortricinae	*Homona coffearia*		Indo-Malaya	Woody
Lepidoptera	Tortricoidea	Tortricidae	Tortricinae	*Pandemis limitata*	Yes	Nearctic	Woody
Lepidoptera	Yponomeutoidea	Yponomeutidae	Plutellinae	*Plutella maculipennis*	Yes	Cosmopolitan	Herbaceous
Lepidoptera	Yponomeutoidea	Yponomeutidae	Plutellinae	*Plutella xylostella*	Yes	Cosmopolitan	Herbaceous
Lepidoptera	Zygaenoidea	Zygaenidae	Procridinae	*Harrisina brillians*		Nearctic	Woody
Lepidoptera	Zygaenoidea	Limacodidae	Limacodinae	*Iragoides fasciata*		Palearctic	Woody
Lepidoptera	Zygaenoidea	Limacodidae	Limacodinae	*Macroplectra nararia*		Indo-Malaya	Woody
Lepidoptera	Zygaenoidea	Limacodidae	Limacodinae	*Oxyplax ochracea*	Yes	Palearctic	Woody

Table S3. Baculovirus species delimitation

Putative baculovirus species†	Baculovirus Isolates
**Autographa californica NPV-A**<br>***Autographa californica multiple nucleopolyhedrovirus***	Autographa californica NPV Ayres M D<br>Autographa californica NPV C6 Tao X Y<br>Autographa californica NPV L-1 Passarelli A L<br>Autographa californica NPV E2 Guarino L A<br>Autographa californica NPV WP10 Chateigner A<br>Autographa californica NPV C6 Possee R D<br>Rachiplusia nu NPV Rodriguez V A<br>Autographa californica NPV 1361 Rowley D L<br>Autographa californica NPV Hooft van Iddekinge B J<br>Autographa californica NPV Gearing K L<br>Hyphantria cunea NPV Lee H H<br>Autographa californica NPV E2 Maghodia A B<br>Autographa californica NPV vAcRev-1 Wu C<br>Autographa californica NPV vAcRev-2 Wu C<br>Plutella maculipennis NPV A15-2 Jehle J A<br>Autographa californica NPV 1004 Rowley D L<br>Autographa californica NPV A12-2 Jehle J A<br>Autographa gamma NPV 474 Rowley D L<br>Autographa californica NPV 1199 Rowley D L<br>Autographa californica NPV 1412 Rowley D L<br>Autographa californica NPV 3092 Rowley D L<br>Autographa californica NPV 683 Rowley D L<br>Autographa californica NPV 570 Rowley D L<br>Autographa californica NPV 578 Rowley D L<br>Autographa californica NPV 1756 Rowley D L<br>Autographa californica NPV 555 Rowley D L<br>Autographa californica NPV 2162 Rowley D L<br>Autographa californica NPV 3114 Rowley D L<br>Autographa californica NPV Carstens E B<br>Autographa californica NPV 582 Rowley D L<br>Autographa californica NPV 162 Rowley D L<br>Autographa californica NPV 228 Rowley D L<br>Autographa californica NPV 397 Rowley D L<br>Autographa californica NPV 458 Rowley D L<br>Autographa nigrisigna NPV Mz-B Mukawa S<br>Autographa californica NPV 396 Rowley D L<br>Galleria mellonella NPV A16-3 Jehle J A<br>Autographa californica NPV 465 Rowley D L<br>Autographa californica NPV 3035 Rowley D L<br>Autographa californica NPV Pennock G D<br>Autographa californica NPV Biotrol VTN Rowley D L<br>Junonia coenia NPV M30-5 Lange M<br>Galleria mellonella NPV A11-3 Jehle J A<br>Galleria mellonella NPV A3-6 Jehle J A<br>Galleria mellonella NPV 1138 Rowley D L<br>Plutella xylostella NPV CL3 Harrison R L<br>Autographa biloba NPV Jehle J A<br>Autographa californica NPV 282 Rowley D L<br>Autographa californica NPV 1417 Rowley D L<br>Autographa californica NPV 3001 Rowley D L<br>Autographa californica NPV 1180 Rowley D L<br>Autographa californica NPV S43 Jehle J A<br>Autographa californica NPV 1176 Rowley D L<br>Diaphania pulverulentalis NPV Priyadharshini P<br>Autographa californica NPV Vail 8 Popham H J R
**Rachiplusia ou NPV**	Anagrapha falcifera NPV Federici B A<br>Rachiplusia ou NPV 3036 Rowley D L<br>Rachiplusia ou NPV 2436 Rowley D L<br>Rachiplusia ou NPV 1239 Rowley D L<br>Rachiplusia ou NPV RI Harrison R L<br>Rachiplusia ou NPV Harrison R L<br>Anagrapha falcifera NPV 3135 Rowley D L<br>Anagrapha falcifera NPV A5-3 Jehle J A<br>Anagrapha falcifera NPV DN10 Rose J

**Bombyx mori NPV**<br>*Bombyx mori nucleopolyhedrovirus*	Bombyx mori NPV T3 Kamita S G<br>Bombyx mori NPV H4 Bando H<br>Bombyx mori NPV GXWX Liang X<br>Bombyx mori NPV India Fan H W<br>Bombyx mori NPV GXLuoC2 Liang X<br>Bombyx mori NPV GXLS Liang X<br>Bombyx mori NPV GXLeY Liang X<br>Bombyx mori NPV GXZS Liang X<br>Bombyx mori NPV GXPN Liang X<br>Bombyx mori NPV GXPB Liang X<br>Bombyx mori NPV GXXC Liang X<br>Bombyx mori NPV GXPG Liang X<br>Bombyx mori NPV GXWM Liang X<br>Bombyx mori NPV GXRA Liang X<br>Bombyx mori NPV GXTX Liang X<br>Bombyx mori NPV GXNN Liang X<br>Bombyx mori NPV GXNP Liang X<br>Bombyx mori NPV GXMS1 Liang X<br>Bombyx mori NPV GXRX Liang X<br>Bombyx mori NPV GXRS Liang X<br>Bombyx mori NPV GXFM Liang X<br>Bombyx mori NPV Kaewwises M<br>Bombyx mori NPV GXLiuC Liang X<br>Bombyx mori NPV GXYZ1 Liang X<br>Bombyx mori NPV GXTL Liang X<br>Bombyx mori NPV GXYJ Liang X<br>Bombyx mori NPV GXLingY Liang X<br>Bombyx mori NPV GXZP Liang X<br>Bombyx mori NPV GXYZ2 Liang X<br>Bombyx mori NPV GXQT Liang X<br>Bombyx mori NPV GXXD1 Liang X<br>Bombyx mori NPV GXSL Liang X<br>Bombyx mori NPV GXMS2 Liang X<br>Bombyx mori NPV GXXZ Liang X<br>Bombyx mori NPV GXLJ Liang X<br>Bombyx mori NPV GXXD2 Liang X<br>Bombyx mori NPV GXCW Liang X<br>Bombyx mori NPV GXGB Liang X<br>Bombyx mori NPV GXDA Liang X<br>Bombyx mori NPV YN1 Tang F F<br>Bombyx mori NPV C6 Kim M<br>Bombyx mori NPV K1 Kang S K<br>Bombyx mandarina NPV Weide S<br>Bombyx mori NPV C1 Kim M<br>Bombyx mori NPV C2 Choi J H<br>Bombyx mori NPV S9 Jehle J A<br>Bombyx mori NPV D1 Hashimoto Y<br>Bombyx mori NPV Iatrou K<br>Bombyx mori NPV M28-4 Lange M<br>Bombyx mori NPV Maeda S<br>Bombyx mori NPV GXYF Liang X<br>Bombyx mori NPV Brazilian Ardisson-Araujo D M<br>Bombyx mori NPV GXLuoC1 Liang X<br>Bombyx mandarina NPV S1 Xu Y P<br>Bombyx mori NPV GXGN Liang X<br>Bombyx mori NPV GXHJ Liang X<br>Bombyx mori NPV GXHS Liang X<br>Bombyx mori NPV GXBY Liang X<br>Bombyx mori NPV Zhejiang Xu Y P<br>Bombyx mori NPV GXHP Liang X<br>Bombyx mori NPV Guangxi Xu Y P<br>Bombyx mori NPV GXNM Liang X<br>Bombyx mori NPV GXHX Liang X<br>Bombyx mori NPV S12 Jehle J A<br>Bombyx mori NPV Cubic Cheng R L<br>Bombyx mori NPV Chu R<br>Bombyx mandarina NPV S2 Xu Y P<br>Bombyx mori NPV Thailand Zhou J B<br>Bombyx mori NPV GXBB Liang X
**Maruca vitrata NPV**<br>*Maruca vitrata nucleopolyhedrovirus*	Maruca vitrata NPV Lee S<br>Maruca vitrata NPV Chen Y R
**Thysanoplusia orichalcea NPV**<br>*Thysanoplusia orichalcea nucleopolyhedrovirus*	Thysanoplusia orichalcea NPV Cheng X W<br>Thysanoplusia orichalcea NPV Wang L<br>Thysanoplusia orichalcea NPV p2 Wang Y S<br>Thysanoplusia orichalcea NPV A28-1 Jehle J A
**Cynosarga chrysolopa NPV**	Cynosarga chrysolopa NPV 302 Herniou E A/Theze J
**Pterolocera amplicornis NPV**	Pterolocera amplicornis NPV M36-2 Lange M
**Dirphia peruvianus NPV**	Dirphia peruvianus NPV A3-1 Jehle J A
**Amsacta albistriga NPV**	Amsacta albistriga NPV Premkumar A

**Cadra cautella NPV**	Cadra cautella NPV c5 Herniou E A/Theze J
**Iragoides fasciata NPV**	Iragoides fasciata NPV Hangzhou Yang L R
**Oxyplax ochracea NPV**	Oxyplax ochracea NPV 435 Wang J
**Aporia crataegi NPV**	Aporia crataegi NPV M45-3 Lange M
**Dendrolimus kikuchii NPV**	Dendrolimus kikuchii NPV YN Yang M M
**Cyclophragma undans NPV**	Cyclophragma undans NPV Whiov Zhu Z
**Coloradia pandora NPV**	Coloradia pandora NPV 19 Rohrmann G F<br>Coloradia pandora NPV M30-2 Lange M
**Lonomia obliqua NPV**	Lonomia obliqua NPV Wolff J L C<br>Lonomia obliqua NPV SP 2000 Clara A S W
**Euproctis similis NPV**	Euproctis chrysorrhoea NPV a10 Herniou E A/Theze J<br>Euproctis similis NPV 768 Herniou E A/Theze J
**Catopsilia pomona NPV**<br>***Catopsilia pomona nucleopolyhedrovirus***	Catopsilia pomona NPV S16 Jehle J A<br>Catopsilia pomona NPV 416 Wang J
**Tineola bisselliella NPV**	Tineola bisselliella NPV M50-4 Jehle J A
**Choristoneura fumiferana NPV**<br>***Choristoneura fumiferana multiple nucleopolyhedrovirus***	Choristoneura fumiferana NPV T3-NPV Tortrivirus Rieth A<br>Choristoneura fumiferana NPV Ireland Poloumienko A<br>Choristoneura fumiferana NPV Lee H Y<br>Choristoneura occidentalis NPV British Columbia 2006 Graham R I<br>Choristoneura occidentalis NPV BC 1 Thumbi D K
**Choristoneura murinana NPV**<br>***Choristoneura murinana nucleopolyhedrovirus***	Choristoneura murinana NPV 26 Rohrmann G F<br>Choristoneura murinana NPV Darmstadt Rohrmann G F<br>Archips rosanus NPV A8no2 Jehle J A
**Archips cerasivoranus NPV**	Archips cerasivoranus NPV Rieth A
**Choristoneura rosaceana NPV**<br>***Choristoneura rosaceana nucleopolyhedrovirus***	Choristoneura rosaceana NPV Lucarotti C J<br>Choristoneura rosaceana NPV NB 1 Thumbi D K
**Orgyia pseudotsugata NPV-A**<br>***Orgyia pseudotsugata multiple nucleopolyhedrovirus***	Orgyia pseudotsugata NPV Leisy D<br>Orgyia pseudotsugata NPV Ahrens C H<br>Leucoma salicis NPV Jakubowska A K<br>Dasychira pudibunda NPV ML1 Krejmer M
**Perina nuda NPV**	Perina nuda NPV Chou C M
**Abraxas grossulariata NPV**	Abraxas grossulariata NPV 112 Herniou E A/Theze J
**Hyphantria cunea NPV-A**<br>***Hyphantria cunea nucleopolyhedrovirus***	Spilosoma obliqua NPV IIPR Akram M<br>Hyphantria cunea NPV Alves C A<br>Hyphantria cunea NPV Tokyo Croizier L<br>Spilosoma obliqua NPV IIPRnpv1 Akram M
**Lemyra imparilis NPV**	Lemyra imparilis NPV n2 Herniou E A/Theze J
**Spilosoma phasma NPV**	Spilosoma phasma NPV S3 Jehle J A
**Hyphantria cunea NPV-B**	Hyphantria cunea NPV S27 Jehle J A
**Spilarctia obliqua NPV**	Spilarctia obliqua NPV IISR-NPV-02 Senthil Kumar C M
**Dione juno NPV**	Dione juno NPV tmk1 1 Rodriguez V A<br>Dione juno NPV tmk1 2 Rodriguez V A
**Agraulis vanillae NPV**	Agraulis vanillae NPV 779 Herniou E A/Theze J
**Heliconius erato NPV**	Heliconius erato NPV 789 Herniou E A/Theze J
**Agraulis sp NPV**	Agraulis sp NPV M34-3 Jehle J A
**Phryganidia californica NPV**	Phryganidia californica NPV M36-3 Lange M
**Idaea seriata NPV**	Idaea seriata NPV 402 Herniou E A/Theze J
**Nepytia phantasmaria NPV**	Nepytia phantasmaria NPV A25-5 Jehle J A

**Antheraea pernyi NPV**<br>*Antheraea pernyi nucleopolyhedrovirus*	Antheraea yamamai NPV Nagano 1 Sasaki K<br>Antheraea proylei NPV Manipur Sasaki K<br>Antheraea pernyi NPV Liaoning 3 Sasaki K<br>Antheraea yamamai NPV Nagano 2 Sasaki K<br>Antheraea pernyi NPV Yuwen H<br>Antheraea pernyi NPV Shi S L<br>Antheraea yamamai NPV Nagano Sasaki K<br>Samia cynthia NPV Nagano Sasaki K<br>Antheraea pernyi NPV Liaoning 1 Sasaki K<br>Antheraea pernyi NPV A Kobayashi J<br>Antheraea pernyi NPV Liaoning 2 Sasaki K<br>Antheraea pernyi NPV Liaoning AnpeMNPV-L2 Fan Q<br>Antheraea pernyi NPV S5 Jehle J A<br>Antheraea pernyi NPV S4 Jehle J A<br>Antheraea pernyi NPV Liaoning Nie Z M<br>Actias selene NPV ST28 Skowron M A<br>Actias selene NPV S1 Jehle J A<br>Actias selene NPV S2 Jehle J A<br>Attacus ricini NPV Hu J<br>Samia ricini NPV Mondulkiri Sasaki K<br>Samia ricini NPV Guangxi 3 Sasaki K<br>Samia ricini NPV Son La Sasaki K<br>Samia ricini NPV Guangxi 1 Sasaki K<br>Samia ricini NPV Guangxi 2 Sasaki K<br>Samia ricini NPV Guangxi Sasaki K<br>Samia cynthia NPV S36 Jehle J A<br>Philosamia cynthia ricini NPV Qian H
**Utetheisa pulchella NPV**	Utetheisa pulchella NPV 142 Herniou E A/Theze J
**Pareuchaetes pseudoinsulata NPV**	Pareuchaetes pseudoinsulata NPV 175 Herniou E A/Theze J
**Anticarsia gemmatalis NPV**<br>*Anticarsia gemmatalis multiple nucleopolyhedrovirus*	Anticarsia gemmatalis NPV AgMNPV-Ibip Ferreira B C<br>Anticarsia gemmatalis NPV AgMNPV-Dour Ferreira B C<br>Anticarsia gemmatalis NPV AgMNPV-Urug Ferreira B C<br>Anticarsia gemmatalis NPV AgMNPV-98 99 Ferreira B C<br>Anticarsia gemmatalis NPV AgMNPV-2D Oliveira J V<br>Anticarsia gemmatalis NPV AgMNPV-Lond Ferreira B C<br>Anticarsia gemmatalis NPV AgMNPV-97 98 Ferreira B C<br>Anticarsia gemmatalis NPV AgMNPV-93 94 Ferreira B C<br>Anticarsia gemmatalis NPV AgMNPV-94 95 Ferreira B C<br>Anticarsia gemmatalis NPV AgMNPV-01 02 Ferreira B C<br>Anticarsia gemmatalis NPV AgMNPV-89 90 Ferreira B C<br>Anticarsia gemmatalis NPV AgMNPV-87 88 Ferreira B C<br>Anticarsia gemmatalis NPV AgMNPV-88 89 Ferreira B C<br>Anticarsia gemmatalis NPV AgMNPV-02 03 Ferreira B C<br>Anticarsia gemmatalis NPV AgMNPV-86 87 Ferreira B C<br>Anticarsia gemmatalis NPV AgMNPV-85 86 Ferreira B C<br>Anticarsia gemmatalis NPV AgMNPV-99 00 Ferreira B C<br>Anticarsia gemmatalis NPV AgMNPV-90 91 Ferreira B C<br>Anticarsia gemmatalis NPV AgMNPV-79 Ferreira B C<br>Anticarsia gemmatalis NPV AgMNPV-00 01 Ferreira B C<br>Anticarsia gemmatalis NPV AgMNPV-91 92 Ferreira B C<br>Anticarsia gemmatalis NPV AgMNPV-96 97 Ferreira B C<br>Anticarsia gemmatalis NPV AgMNPV-92 93 Ferreira B C<br>Anticarsia gemmatalis NPV AgMNPV-PF Ferreira B C<br>Anticarsia gemmatalis NPV AgMNPV-Arg Ferreira B C<br>Anticarsia gemmatalis NPV AgMNPV-84 85 Ferreira B C<br>Anticarsia gemmatalis NPV AgMNPV-42 Brito A F<br>Anticarsia gemmatalis NPV AgMNPV-32 Brito A F<br>Anticarsia gemmatalis NPV AgMNPV-43 Brito A F<br>Anticarsia gemmatalis NPV AgMNPV-39 Brito A F<br>Anticarsia gemmatalis NPV AgMNPV-Pelot Ferreira B C<br>Anticarsia gemmatalis NPV AgMNPV-95 96 Ferreira B C<br>Anticarsia gemmatalis NPV AgMNPV-36 Brito A F<br>Anticarsia gemmatalis NPV AgMNPV-35 Brito A F<br>Anticarsia gemmatalis NPV AgMNPV-33 Brito A F<br>Anticarsia gemmatalis NPV AgMNPV-CM Ferreira B C<br>Anticarsia gemmatalis NPV AgMNPV-37 Brito A F<br>Anticarsia gemmatalis NPV 2D Zanotto P M<br>Anticarsia gemmatalis NPV AgMNPV-26 Brito A F<br>Anticarsia gemmatalis NPV AgMNPV-30 Brito A F<br>Anticarsia gemmatalis NPV AgMNPV-38 Brito A F<br>Anticarsia gemmatalis NPV AgMNPV-28 Brito A F<br>Anticarsia gemmatalis NPV AgMNPV-34 Brito A F<br>Anticarsia gemmatalis NPV AgMNPV-40 Brito A F<br>Anticarsia gemmatalis NPV AgMNPV-31 Brito A F<br>Anticarsia gemmatalis NPV AgMNPV-29 Brito A F<br>Anticarsia gemmatalis NPV AgMNPV-27 Brito A F
**Choristoneura fumiferana DEF NPV**<br>*Choristoneura fumiferana DEF multiple nucleopolyhedrovirus*	Choristoneura fumiferana DEF NPV Li X

**Amorbia cuneana NPV**	Amorbia cuneacapsa NPV A8-3 Jehle J A<br>Amorbia cuneana NPV Sciocco A
**Neophasia sp NPV**	Neophasia sp NPV 11 Rohrmann G F
**Condylorrhiza vestigialis NPV**	Condylorrhiza vestigialis NPV Castro M E
**Epiphyas postvittana NPV**<br>*Epiphyas postvittana nucleopolyhedrovirus*	Epiphyas postvittana NPV Hyink O
**Spodoptera frugiperda NPV**<br>*Spodoptera frugiperda multiple nucleopolyhedrovirus*	Spodoptera frugiperda NPV 635 Rowley D L<br>Spodoptera frugiperda NPV 636 Rowley D L<br>Spodoptera frugiperda NPV 459 Rowley D L<br>Spodoptera frugiperda NPV 2705 Rowley D L<br>Spodoptera frugiperda NPV Colombian Barrera G P<br>Spodoptera frugiperda NPV 19 Wolff J L<br>Spodoptera frugiperda NPV 1 Rowley D L<br>Spodoptera frugiperda NPV 3146 Rowley D L<br>Spodoptera frugiperda NPV 5 Rowley D L<br>Spodoptera frugiperda NPV 637 Rowley D L<br>Spodoptera frugiperda NPV Guasave Escobedo-Bonilla C M<br>Spodoptera frugiperda NPV 2 Rowley D L<br>Autographa californica NPV Gonzalez M A<br>Spodoptera frugiperda NPV Gonzalez M A<br>Spodoptera frugiperda NPV 2 Simon O<br>Spodoptera frugiperda NPV 1 Simon O<br>Spodoptera frugiperda NPV 281 Rowley D L<br>Spodoptera frugiperda NPV 3 Rowley D L<br>Spodoptera frugiperda NPV 3AP2 Harrison R L<br>Spodoptera frugiperda NPV 4 Rowley D L<br>Spodoptera frugiperda NPV 6 Rowley D L<br>Spodoptera frugiperda NPV 638 Rowley D L
**Spodoptera exigua NPV**<br>*Spodoptera exigua multiple nucleopolyhedrovirus*	Spodoptera exigua NPV VT-SeOx4 Theze J<br>Spodoptera exigua NPV VT-SeAl2 Theze J<br>Spodoptera exigua NPV VT-SeAl1 Theze J<br>Spodoptera exigua NPV HT-SeG25 Theze J<br>Spodoptera exigua NPV HT-SeG26 Theze J<br>Spodoptera exigua NPV HT-SeG24 Theze J<br>Spodoptera exigua NPV HT-SeSP2A Theze J<br>Spodoptera exigua NPV El-Dougdoug K A<br>Spodoptera exigua NPV YV Darsouei R<br>Spodoptera exigua NPV IJkel W F
**Spodoptera albula NPV**	Spodoptera albula NPV k7 Herniou E A/Theze J
**Autographa californica NPV-B**	Autographa californica NPV-B 1059 Rowley D L
**Spodoptera litura NPV-B**	Spodoptera litura NPV II Li Y
**Spodoptera exempta NPV**	Spodoptera exempta NPV var1 Graham R I<br>Spodoptera exempta NPV k11 Herniou E A/Theze J<br>Achaea faber NPV 64 Herniou E A/Theze J
**Agrotis ipsilon NPV**<br>*Agrotis ipsilon multiple nucleopolyhedrovirus*	Agrotis ipsilon NPV M6-2 Lange M<br>Agrotis ipsilon NPV Illinois Harrison R L<br>Agrotis ipsilon NPV AgipNPV Agip Lange M<br>Agrotis ipsilon NPV AgipNPV Agse Lange M<br>Agrotis ipsilon NPV Kentucky Barney W E<br>Agrotis ipsilon NPV Illinois Barney W E
**Agrotis segetum NPV-B**<br>*Agrotis segetum nucleopolyhedrovirus B*	Agrotis exclamationis NPV Jakubowska A K<br>Agrotis segetum NPV A AgseNPV-UK Jakubowska A<br>Agrotis segetum NPV A A12-3 Jehle J A<br>Agrotis segetum NPV B English Wennmann J T<br>Agrotis exclamationis NPV JW11-12 232-422 Wennmann J T<br>Agrotis segetum NPV A Lange M
**Agrotis segetum NPV-A**<br>*Agrotis segetum nucleopolyhedrovirus A*	Agrotis segetum NPV A AgseNPV-P Jakubowska A<br>Agrotis segetum NPV A Jakubowska A K
**Cerapteryx graminis NPV**	Cerapteryx graminis NPV V1 Graham R I

**Mamestra configurata NPV-B**<br>*Mamestra configurata nucleopolyhedrovirus B*<br>*Mamestra brassicae multiple nucleopolyhedrovirus*	Helicoverpa armigera NPV 3154 Rowley D L<br>Helicoverpa armigera NPV 131 Rowley D L<br>Helicoverpa armigera NPV 449 Rowley D L<br>Helicoverpa armigera NPV 120 Rowley D L<br>Helicoverpa armigera NPV 443 Rowley D L<br>Mamestra configurata NPV B Li L<br>Mamestra brassicae NPV Oxford Cameron I R<br>Mamestra brassicae NPV A10-1 Jehle J A<br>Mamestra brassicae NPV K1 Choi J B<br>Mamestra brassicae NPV Tokyo Mukawa S<br>Helicoverpa armigera NPV 3110 Rowley D L<br>Mamestra brassicae NPV CTa Hou D<br>Helicoverpa armigera NPV 1072 Rowley D L<br>Mamestra brassicae NPV S33 Jehle J A<br>Helicoverpa armigera NPV Tang P<br>Helicoverpa armigera NPV 3153 Rowley D L<br>Mamestra brassicae NPV CHb1 Liu L<br>Leucania separata NPV 1 Wang J<br>Leucania separata NPV 2 Wang J<br>Mythimna separata NPV Geihoku Kouassi L N
**Panolis flammea NPV**	Panolis flammea NPV i3 Herniou E A/Theze J
**Mamestra brassicae NPV**	Mamestra brassicae NPV A3-5 Jehle J A
**Mamestra configurata NPV-A**<br>*Mamestra configurata nucleopolyhedrovirus A*	Mamestra configurata NPV A 90 4 Li L<br>Mamestra configurata NPV A 90 2 Li S
**Mythimna unipuncta NPV-B**	Pseudaletia sp NPV 7 Rohrmann G F<br>Mythimna unipuncta NPV #7 Harrison R L<br>Mythimna unipuncta NPV 1411 Keathley C P<br>Mythimna unipuncta NPV 330 Keathley C P
**Peridroma margaritosa NPV**	Peridroma sp NPV 167 Rohrmann G F<br>Peridroma sp NPV GR 167 Rohrmann G F<br>Peridroma margaritosa NPV A25-4 Jehle J A
**Trichoplusia ni NPV**<br>*Trichoplusia ni single nucleopolyhedrovirus*	Trichoplusia ni NPV 1004 Rowley D L<br>Trichoplusia ni NPV 2703 Rowley D L<br>Trichoplusia ni NPV 252 Rowley D L<br>Trichoplusia ni NPV 2700 Rowley D L<br>Trichoplusia ni NPV 1237 Rowley D L<br>Trichoplusia ni NPV 1141 Rowley D L<br>Trichoplusia ni NPV 1185 Rowley D L<br>Trichoplusia ni NPV 1241 Rowley D L<br>Trichoplusia ni NPV 246 Rowley D L<br>Trichoplusia ni NPV 399 Rowley D L<br>Trichoplusia ni NPV 270 Rowley D L<br>Trichoplusia ni NPV 239 Rowley D L<br>Trichoplusia ni NPV 3091 Rowley D L<br>Trichoplusia ni NPV 3073 Rowley D L<br>Trichoplusia ni NPV 282 Rowley D L<br>Trichoplusia ni NPV 397 Rowley D L<br>Trichoplusia ni NPV 227 Rowley D L<br>Trichoplusia ni NPV 207 Rowley D L<br>Trichoplusia ni NPV 455 Rowley D L<br>Trichoplusia ni NPV 2541 Rowley D L<br>Trichoplusia ni NPV 271 Rowley D L<br>Trichoplusia ni NPV Willis L G<br>Trichoplusia ni NPV 242 Rowley D L<br>Trichoplusia ni NPV 209 Rowley D L<br>Trichoplusia ni NPV 230 Rowley D L
**Chrysodeixis includens NPV**<br>*Chrysodeixis includens nucleopolyhedrovirus*	Chrysodeixis includens NPV IG Craveiro S R<br>Pseudoplusia includens NPV IE Craveiro S R<br>Chrysodeixis includens NPV 2 Craveiro S R<br>Chrysodeixis includens NPV 1 Craveiro S R<br>Pseudoplusia includens NPV 458 Rowley D L<br>Chrysodeixis includens NPV IC Craveiro S R<br>Chrysodeixis includens NPV IB Craveiro S R<br>Chrysodeixis includens NPV IA Craveiro S R<br>Chrysodeixis includens NPV IE Craveiro S R<br>Chrysodeixis includens NPV IF Craveiro S R<br>Chrysodeixis includens NPV GO Morgado F S<br>Chrysodeixis includens NPV ID Craveiro S R<br>Pseudoplusia includens NPV Xu F<br>Pseudoplusia includens NPV Los Angeles Xu F
**Chrysodeixis chalcites NPV**<br>*Chrysodeixis chalcites nucleopolyhedrovirus*	Chrysodeixis chalcites NPV Xu F<br>Chrysodeixis chalcites NPV TF1 4 Bernal A<br>Chrysodeixis chalcites NPV TF1 1 Bernal A<br>Chrysodeixis chalcites NPV TF1 3 Bernal A<br>Chrysodeixis chalcites NPV van Oers M M<br>Chrysodeixis chalcites NPV TF1 Bernal A<br>Chrysodeixis chalcites NPV TF1-A Bernal A<br>Chrysodeixis chalcites NPV TF1 2 Bernal A<br>Plusia acuta NPV A14-5 Jehle J A<br>Trichoplusia ni NPV Fielding B C

**Trichoplusia orichalcea NPV**	Trichoplusia orichalcea NPV b9 Herniou E A/Theze J
**Autographa nigrisigna NPV**	Autographa nigrisigna NPV Mz-A Mukawa S
**Lymantria dispar NPV**<br>***Lymantria dispar multiple nucleopolyhedrovirus***	Lymantria dispar NPV 3065 Harrison R L<br>Lymantria dispar NPV 3152 Harrison R L<br>Lymantria dispar NPV Ab-a624 Harrison R L<br>Lymantria dispar NPV LdMNPV-45 0 Martemyanov V V<br>Lymantria dispar NPV Massachusetts Podgwaite J D<br>Lymantria dispar NPV A21-MPV Bischoff D S<br>Lymantria dispar NPV 3063 Harrison R L<br>Lymantria dispar NPV 1010 Harrison R L<br>Lymantria dispar NPV RR01 Krejmer-Rabalska M<br>Lymantria dispar NPV A24-6 Jehle J A<br>Lymantria dispar NPV T1 Gencer D<br>Lymantria dispar NPV T3 Gencer D<br>Lymantria dispar NPV T2 Gencer D<br>Lymantria dispar NPV T4 Gencer D<br>Lymantria dispar NPV 3057 Harrison R L<br>Lymantria dispar NPV New Jersey Podgwaite J D<br>Lymantria dispar NPV Kuzio J<br>Lymantria xylina NPV 2 Nai Y S<br>Lymantria dispar NPV New York Podgwaite J D<br>Lymantria dispar NPV 3029 Harrison R L<br>Lymantria dispar NPV Kashmir Gani M<br>Lymantria dispar NPV Ld Ninohe#1 Takatsuka J<br>Lymantria dispar NPV LdMNPV-27 0 Kabilov M R<br>Lymantria dispar NPV Karasuk Bakhvalov S A<br>Lymantria dispar NPV Tatarsk Bakhvalov S A<br>Lymantria dispar NPV Chistoozernyi Bakhvalov S A<br>Lymantria dispar NPV 3058 Harrison R L<br>Lymantria dispar NPV HrB Harrison R L<br>Lymantria dispar NPV LdMNPV-27 2 Kabilov M R<br>Lymantria dispar NPV An C<br>Lymantria dispar NPV 3054 Harrison R L<br>Lymantria dispar NPV 2161 Harrison R L<br>Lymantria dispar NPV 3041 Harrison R L
**Lymantria xylina NPV**<br>***Lymantria xylina nucleopolyhedrovirus***	Lymantria xylina NPV Nai Y S<br>Lymantria xylina NPV LyxyMNPV-5 Nai Y S<br>Lymantria xylina NPV Wu C Y<br>Lymantria xylina NPV 3061 Harrison R L<br>Lymantria xylina NPV S31 Jehle J A
**Lymantria mathura mutiple NPV**	Lymantria mathura mutiple NPV Lyma Kunohe A#1 Takatsuka J<br>Lymantria mathura mutiple NPV Lyma Kunohe B#1 Takatsuka J
**Lymantria monacha NPV**	Lymantria monacha NPV A19-3 Jehle J A<br>Lymantria monacha NPV 463 Harrison R L<br>Lymantria monacha NPV A14-3 Jehle J A<br>Lymantria dispar NPV BNP Rabalski L
**Hyposidra talaca NPV**	Hyposidra talaca NPV Antony B<br>Hyposidra infixaria NPV Antony B<br>Buzura suppressaria NPV Antony B<br>Hyposidra talaca NPV HytaNPV Dasgupta S<br>Hyposidra talaca NPV Terai K1 Ghosh B
**Ectropis obliqua NPV**<br>***Ectropis obliqua nucleopolyhedrovirus***	Ectropis grisescens NPV S22 Jehle J A<br>Ectropis obliqua NPV unioasis 1 Chen J<br>Ectropis obliqua NPV A1 Ma X C<br>Boarmia bistortata NPV A5-4 Lange M
**Hemileuca sp NPV**	Hemileuca sp NPV 165 Rohrmann G F<br>Hemileuca sp NPV Rohrmann G F
**Buzura suppressaria NPV**<br>***Buzura suppressaria nucleopolyhedrovirus***	Buzura suppressaria NPV GX-BsNPV Luo J<br>Buzura suppressaria NPV S13 Jehle J A<br>Buzura suppressaria NPV Guangxi Luo J<br>Buzura suppressaria NPV HB Hu Z H<br>Buzura suppressaria NPV Hubei Hu Z H<br>Buzura suppressaria NPV Terai K1 Ghosh B
**Sucra jujuba NPV**<br>***Sucra jujuba nucleopolyhedrovirus***	Sucra jujuba NPV 473 Liu X
**Orgyia pseudotsugata NPV-B**	Orgyia pseudotsugata NPV Jakubowska A<br>Hemerocampa vetusta NPV A24-5 Jehle J A<br>Orgyia antiqua NPV a9 Herniou E A/Theze J
**Orgyia anartoides NPV**	Orgyia anartoides NPV Oa-8 Bulach D M
**Orgyia leucostigma NPV**<br>***Orgyia leucostigma nucleopolyhedrovirus***	Orgyia leucostigma NPV CFS-77 Eveleigh R J M
**Dasychira plagiata NPV**	Dasychira plagiata NPV M36-8 Jehle J A
**Orgyia mixta NPV**	Orgyia mixta NPV 67 Herniou E A/Theze J<br>Aroa discalis NPV 63 Herniou E A/Theze J
**Euproctis pseudoconspersa NPV**<br>***Euproctis pseudoconspersa nucleopolyhedrovirus***	Euproctis pseudoconspersa NPV A13-1 Jehle J A<br>Euproctis pseudoconspersa NPV A4-5 Jehle J A<br>Euproctis pseudoconspersa NPV Hangzhou Tang X D

**Euproctis digramma NPV**	Euproctis digramma NPV S24 Jehle J A
**Apocheima cinerarium NPV**	Apocheima cinerarium NPV Qu L J<br>Apocheima cinerarium NPV Zhang Y A<br>Apocheima cinerarium NPV S7 Jehle J A
**Erannis defoliaria NPV**	Erannis defoliaria NPV 174 Herniou E A/Theze J
**Lambdina fiscellaria NPV**<br>***Lambdina fiscellaria nucleopolyhedrovirus***	Lambdina fiscellaria NPV GR15 Rohrmann G F
**Trabala vishnou NPV**	Trabala vishnou NPV 195 Herniou E A/Theze J
**Perigonia lusca NP**	Perigonia lusca NPV Ardisson-Araujo D M
**Clanis bilineata NPV**<br>***Clanis bilineata nucleopolyhedrovirus***	Clanis bilineata NPV DZ1 Zhu S Y
**Malacosoma californicum NPV**	Malacosoma californicum NPV M30-6 Lange M<br>Malacosoma sp NPV M28-2 Lange M<br>Malacosoma sp NPV 18 Rohrmann G F<br>Malacosoma californicum NPV 99 Cory J<br>Malacosoma sp NPV 164 Rohrmann G F
**Malacosoma americanum NPV**	Malacosoma americanum NPV M39-4 Jehle J A<br>Malacosoma americanum NPV Zeng F
**Malacosoma neustria NPV**	Malacosoma neustria NPV Dmitrenko V V<br>Malacosoma neustria NPV Jankevica L<br>Malacosoma neustria NPV A2-6 Lange M<br>Malacosoma neustria NPV Lange M<br>Malacosoma neustria NPV S32 Jehle J A
**Malacosoma disstria NPV**	Malacosoma disstria NPV MdMNPV-A92 Erlandson M A
**Lasiocampa quercus NPV**	Lasiocampa quercus NPV 202 Herniou E A/Theze J
**Pachypasa papyri NPV**	Pachypasa papyri NPV 436 Herniou E A/Theze J
**Vanessa atalanta NPV**	Aglais urticae NPV a7 Herniou E A/Theze J<br>Vanessa atalanta NPV 54 Herniou E A/Theze J<br>Polygonia c-album NPV 380 Herniou E A/Theze J<br>Vanessa cardui NPV 367 Herniou E A/Theze J
**Nymphalis io NPV**	Nymphalis io NPV 353 Herniou E A/Theze J
**Anomis sabulifera NPV**	Anomis sabulifera NPV 14 Herniou E A/Theze J
**Mahasena corbetti NPV**	Mahasena corbetti NPV 692 Herniou E A/Theze J
**Adoxophyes honmai NPV**<br>***Adoxophyes honmai nucleopolyhedrovirus***	Adoxophyes honmai NPV ADN001 Nakai M
**Adoxophyes orana NPV**	Adoxophyes orana NPV English Hilton S
**Kotochalia junodi NPV**	Kotochalia junodi NPV 32 Herniou E A/Theze J
**Urbanus proteus NPV**	Urbanus proteus NPV Southern Brazil Santos E R
**Epinotia granitalis NPV**	Epinotia granitalis NPV A1 Takatsuka J

**Helicoverpa armigera NPV**<br>*Heliocoverpa armigera nucleopolyhedrovirus*	Helicoverpa zea NPV 543 Rowley D L<br>Helicoverpa zea NPV Elkar Le T H<br>Helicoverpa zea NPV Chen X<br>Helicoverpa zea NPV 1578 Rowley D L<br>Helicoverpa zea NPV 668 Rowley D L<br>Helicoverpa zea NPV 1013 Rowley D L<br>Helicoverpa zea NPV 1024 Rowley D L<br>Helicoverpa armigera NPV F29 Le T H<br>Helicoverpa armigera NPV E17 Le T H<br>Helicoverpa zea NPV Gemstar-35022 Rowley D L<br>Helicoverpa armigera NPV AE20 Le T H<br>Helicoverpa armigera NPV A6 Le T H<br>Helicoverpa zea NPV 1180 Rowley D L<br>Trichoplusia ni NPV Abrahams R<br>Helicoverpa zea NPV HS-18 Ternovoi V A<br>Helicoverpa zea NPV Br South Ardisson-Araujo D M P<br>Helicoverpa armigera NPV 2588 Rowley D L<br>Helicoverpa armigera NPV 1113 Rowley D L<br>Helicoverpa armigera NPV H25EA1 Noune C<br>Helicoverpa zea NPV 3108 Rowley D L<br>Helicoverpa armigera NPV 3104 Rowley D L<br>Helicoverpa zea NPV 566 Rowley D L<br>Helicoverpa gelotopoeon NPV ar Ferrelli M L<br>Helicoverpa NPV AC53 AC53C6 Noune C<br>Helicoverpa NPV AC53 AC53T42 Noune C<br>Helicoverpa NPV AC53 AC53T5 Noune C<br>Helicoverpa NPV AC53 AC53T41 Noune C<br>Helicoverpa NPV AC53 AC53C9 Noune C<br>Helicoverpa NPV AC53 AC53 Noune C<br>Helicoverpa NPV AC53 AC53C1 Noune C<br>Helicoverpa NPV AC53 AC53C5 Noune C<br>Helicoverpa NPV AC53 AC53T2 Noune C<br>Helicoverpa NPV AC53 AC53C3 Noune C<br>Helicoverpa armigera NPV Zhang C<br>Helicoverpa armigera NPV 3010 Rowley D L<br>Helicoverpa armigera NPV 1073 Rowley D L<br>Helicoverpa armigera NPV C1 Zhang C X<br>Helicoverpa armigera NPV 1625 Rowley D L<br>Helicoverpa armigera NPV 2066 Rowley D L<br>Helicoverpa armigera NPV Chen X<br>Helicoverpa armigera NPV G4 Deng F<br>Helicoverpa armigera NPV Australia Zhang H<br>Helicoverpa armigera NPV Khan S<br>Helicoverpa armigera NPV Jodhan Gupta V K<br>Helicoverpa armigera NPV PAU Gupta V K<br>Helicoverpa armigera NPV HAU Gupta V K<br>Helicoverpa armigera NPV Bathinda Gupta V K<br>Helicoverpa armigera NPV PDBC Gupta V K<br>Helicoverpa armigera NPV NNg1 Ogembo J G<br>Busseola fusca NPV A2-4 Lange M<br>Helicoverpa armigera NPV LB6 Arrizubieta M<br>Helicoverpa armigera NPV LB3 Arrizubieta M<br>Helicoverpa armigera NPV LB1 Arrizubieta M<br>Helicoverpa armigera NPV SP1A Arrizubieta M<br>Helicoverpa armigera NPV SP1B Arrizubieta M<br>Helicoverpa armigera NPV 1186 Rowley D L<br>Helicoverpa armigera NPV Ludhiana Ashika T R<br>Helicoverpa armigera NPV Bangalore Jency J<br>Helicoverpa armigera NPV L1 HANPVL1 Rakshit O<br>Helicoverpa armigera NPV 75 Rowley D L<br>Helicoverpa armigera NPV 1221 Rowley D L<br>Helicoverpa armigera NPV 141 Rowley D L<br>Helicoverpa armigera NPV 126 Rowley D L<br>Helicoverpa armigera NPV 1240 Rowley D L<br>Helicoverpa armigera NPV 138 Rowley D L<br>Helicoverpa assulta NPV Korean Woo S D<br>Helicoverpa armigera NPV Palampur Guleria S<br>Helicoverpa armigera NPV Bangalore HA 01 Jose J<br>Helicoverpa armigera NPV 1115 Rowley D L<br>Helicoverpa armigera NPV 1825 Rowley D L<br>Helicoverpa armigera NPV Faridkot Jency J<br>Helicoverpa armigera NPV Faridkot Rakshit O<br>Helicoverpa armigera NPV hingoli Ashika T R<br>Helicoverpa armigera NPV 1623 Rowley D L<br>Helicoverpa zea NPV 1027 Rowley D L<br>Helicoverpa armigera NPV Ludhiana Rakshit O

**Spodoptera littoralis NPV**<br>*Spodoptera littoralis nucleopolyhedrovirus*	Spodoptera littoralis NPV Egy-SLNPV Ahmed Y E<br>Spodoptera littoralis NPV Seufi A<br>Spodoptera littoralis NPV AN1956 Breitenbach J E<br>Spodoptera littoralis NPV Spli1 Takatsuka J<br>Spodoptera littoralis NPV 454 Breitenbach J E<br>Spodoptera littoralis NPV 1628 Breitenbach J E<br>Spodoptera littoralis NPV 1263 Breitenbach J E<br>Spodoptera littoralis NPV SlMNPV-B isolate E15 Faktor O<br>Spodoptera littoralis NPV 3017 Breitenbach J E<br>Spodoptera littoralis NPV 3003 Breitenbach J E<br>Spodoptera littoralis NPV 3032 Breitenbach J E<br>Spodoptera litura NPV K1 Woo S<br>Spodoptera littoralis NPV A9-1 Jehle J A<br>Spodoptera littoralis NPV A26-5 Jehle J A<br>Spodoptera littoralis NPV SIMNPV-M2 Croizier L<br>Spodoptera litura NPV SlNPV-K2 Wang Y<br>Spodoptera littoralis NPV Az Martins T<br>Spodoptera littoralis NPV 1213 Breitenbach J E<br>Spodoptera littoralis NPV 2424 Breitenbach J E
**Spodoptera litura NPV-A**<br>*Spodoptera litura nucleopolyhedrovirus*	Spodoptera litura NPV Lab-1 Kouassi L N<br>Spodoptera litura NPV Os-7 Kouassi L N<br>Spodoptera litura NPV Kouassi L N<br>Spodoptera litura NPV Act-1 Kouassi L N<br>Spodoptera litura NPV Satsuma Kouassi L N<br>Spodoptera terricola NPV A26-1 Jehle J A<br>Spodoptera litura NPV A17-3 Jehle J A<br>Spodoptera litura NPV GZ-1 Wei Y J<br>Spodoptera litura NPV S37 Jehle J A<br>Spodoptera litura NPV G10-3 Zhu J<br>Spodoptera litura NPV G2 Pang Y<br>Spodoptera litura NPV Li C<br>Spodoptera litura NPV Splt1 Takatsuka J<br>Spodoptera litura NPV E8 Bulach D M<br>Spodoptera litura NPV B-0-4 Zhu J<br>Spodoptera litura NPV Bangalore Jose J<br>Spodoptera litura NPV K-3 Zhu J
**Mythimna unipuncta NPV-A**	Mythimna unipuncta NPV KY310 Keathley C P<br>Mythimna unipuncta NPV KY511 Keathley C P
**Mythimna separata NPV**<br>*Leucania separata nucleopolyhedrovirus*	Leucania separata NPV AH1 Du E Q
**Operophtera brumata NPV**	Operophtera brumata NPV OpbuNPV-MA Harrison R L
**Phalera bucephala NPV**	Phalera bucephala NPV 204 Herniou E A/Theze J
**Operophtera bruceata NP**	Operophtera bruceata NPV ME Broadley H J
**Wiseana signata NPV**<br>*Wiseana signata nucleopolyhedrovirus*	Wiseana signata NPV WisiSNPV Sadler T J
**Wiseana cervinata NPV**	Wiseana cervinata NPV 344 Herniou E A/Theze J
**Choristoneura fumiferana GV**<br>*Choristoneura fumiferana granulovirus*	Choristoneura fumiferana GV Bah A<br>Choristoneura fumiferana GV Escasa S R<br>Choristoneura viridis GV 22 Rohrmann G F<br>Choristoneura occidentalis GV British Columbia 2006 Graham R I<br>Choristoneura fumiferana GV Rashidan K K
**Choristoneura murinana GV**	Choristoneura murinana GV A11-1 M50-3 Jehle J A
**Pandemis limitata GV**	Pandemis limitata GV M36-1 Lange M
**Homona coffearia GV**	Homona coffearia GV 745 Herniou E A/Theze J
**Harrisina brillians GV**<br>*Harrisina brillians granulovirus*	Harrisina brillians GV m2 Herniou E A/Theze J
**Anthophila fabriciana GV**	Anthophila fabriciana GV 790 Herniou E A/Theze J
**Macroplectra nararia GV**	Macroplectra nararia GV 254 Herniou E A/Theze J
**Darna trima GV**	Darna trima GV 545 Herniou E A/Theze J
**Amelia pallorana GV**	Amelia pallorana GV M30-1 Lange M
**Erinnyis ello GV**<br>*Erinnyis ello granulovirus*	Erinnyis ello GV ErelGV-00 Brito A F<br>Erinnyis ello GV S86 Ardisson-Araujo D M<br>Erinnyis ello GV ErelGV-99 Brito A F<br>Erinnyis ello GV ErelGV-94 Brito A F<br>Erinnyis ello GV ErelGV-98 Brito A F<br>Erinnyis ello GV M34-4 Jehle J A<br>Erinnyis ello GV ErelGV-AC Brito A F<br>Erinnyis ello GV ErelGV-PA Brito A F
**Andraca bipunctata GV**<br>*Clostera anastomosis granulovirus B*	Clostera anastomosis GV B ClasGV-B Yin F<br>Andraca bipunctata GV S48 Jehle J A<br>Andraca bipunctata GV Zhang D

**Pieris rapae GV**<br>*Artogeia rapae granulovirus*	Pieris rapae GV Chen K<br>Artogeia rapae GV Wuhan Wang X F<br>Pieris brassicae GV S54 Jehle J A<br>Pieris rapae GV E3 Guangxi Wen R<br>Pieris rapae GV S55 Jehle J A<br>Pieris rapae GV Oh S<br>Pieris rapae GV M36-7 Lange M
**Clostera anachoreta GV**<br>*Clostera anachoreta granulovirus*	Clostera anachoreta GV ClanGV-HBHN Liang Z<br>Clostera anachoreta GV Zhang X X<br>Clostera anachoreta GV S49 Jehle J A
**Clostera anastomosis GV**<br>*Clostera anastomosis granulovirus A*	Clostera anastomosis GV Henan CaLGV-Henan Liang Z
**Plathypena scabra GV**	Plathypena scabra GV A25-6 Jehle J A
**Adoxophyes orana GV**<br>*Adoxophyes orana granulovirus*	Adoxophyes orana GV A2-3 Lange M<br>Adoxophyes orana GV A6-5 Jehle J A<br>Adoxophyes orana GV Wormleaton S<br>Adoxophyes orana GV H Kundu J K<br>Adoxophyes orana GV E1 Wormleaton S L<br>Adoxophyes orana GV Capex-L9 Kundu J K<br>Adoxophyes orana GV Capex1-L2 Kundu J K<br>Adoxophyes orana GV S45 Jehle J A<br>Adoxophyes orana GV Miyazaki Nakai M
**Cnaphalocrocis medinalis G**<br>*Cnaphalocrocis medinalis granulovirus*	Cnaphalocrocis medinalis GV Enping Zhang S<br>Cnaphalocrocis medinalis GV Han G
**Cydia pomonella GV**<br>*Cydia pomonella granulovirus*	Cydia pomonella GV I08 Eberle K E<br>Cydia pomonella GV I01 Eberle K E<br>Cydia pomonella GV I12 Eberle K E<br>Cydia pomonella GV CpGV-I12 Gebhardt M M<br>Cydia pomonella GV 217 Arneodo J D<br>Cydia pomonella GV Mexican 1 Crook N E<br>Cydia pomonella GV ALE1 Fan J B<br>Cydia pomonella GV CJ01 Shen J<br>Cydia pomonella GV P118 Arneodo J D<br>Cydia pomonella GV 38 Arneodo J D<br>Cydia pomonella GV I68 Sayed S<br>Cydia pomonella GV I66 Sayed S<br>Cydia pomonella GV I01 Sayed S<br>Cydia pomonella GV I08 Sayed S<br>Cydia pomonella GV I07 Sayed S<br>Cydia pomonella GV M39-1 Jehle J A<br>Cydia pomonella GV 616 Arneodo J D<br>Cydia pomonella GV G02 Eberle K E<br>Cydia pomonella GV I12 Sayed S<br>Cydia pomonella GV A6-4 Jehle J A<br>Cydia pomonella GV I66 Eberle K E<br>Cydia pomonella GV 69 Arneodo J D<br>Cydia pomonella GV ALE2 Fan J B<br>Cydia pomonella GV G02 Sayed S<br>Cydia pomonella GV G01 Sayed S<br>Cydia pomonella GV CpGV-M Gebhardt M M<br>Cydia pomonella GV A11-2 Jehle J A<br>Cydia pomonella GV ZY1 Fan J B<br>Cydia pomonella GV Col19 Arneodo J D<br>Cydia pomonella GV CpGV-S Gebhardt M M<br>Cydia pomonella GV KS1 Fan J B<br>Cydia pomonella GV WW1 Fan J B<br>Cydia pomonella GV E2 Eberle K E<br>Cydia pomonella GV CpGV-E2 Gebhardt M M<br>Cydia pomonella GV I07 Eberle K E<br>Cydia pomonella GV G01 Eberle K E<br>Cydia pomonella GV I68 Eberle K E<br>Cydia pomonella GV CpGV-I07 Gebhardt M M<br>Cydia pomonella GV C1 Arneodo J D<br>Cydia pomonella GV M10 Arneodo J D<br>Cydia pomonella GV C6 Arneodo J D<br>Cydia pomonella GV M3 Arneodo J D<br>Cydia pomonella GV M18 Arneodo J D<br>Cydia pomonella GV P7 Arneodo J D
**Cryptophlebia leucotreta GV**<br>*Cryptophlebia leucotreta granulovirus*	Cryptophlebia leucotreta GV CrleGV-SA Singh S<br>Cryptophlebia leucotreta GV CrleGV-SA van der Merwe M<br>Cryptophlebia leucotreta GV CV3 Jehle J A
**Cnephasia longana GV**	Cnephasia longana GV A2-2 Lange M
**Diatraea saccharalis GV**<br>*Diatraea saccharalis granulovirus*	Diatraea saccharalis GV Parana-2009 Ardisson-Araujo D M
**Phthorimaea operculella GV**<br>*Phthorimaea operculella granulovirus*	Phthorimaea operculella GV SA Jukes M D<br>Phthorimaea operculella GV Croizier L<br>Phthorimaea operculella GV 2 Jukes M D<br>Phthorimaea operculella GV 1 Jukes M D

**Plodia interpunctella GV**<br>*Plodia interpunctella granulovirus*	Plodia interpunctella GV Cambridge Harrison R L
**Epinotia aporema GV**<br>*Epinotia aporema granulovirus*	Epinotia aporema GV Ferrelli M L<br>Epinotia aporema GV Parola A D
**Agrotis segetum GV**<br>*Agrotis segetum granulovirus*	Agrotis segetum GV L1 Zhang X<br>Agrotis segetum GV DA Gueli Alletti G<br>Agrotis segetum GV A17-5 Jehle J A<br>Agrotis exclamationis GV S46 Jehle J A<br>Agrotis segetum GV Ai X L<br>Agrotis segetum GV S47 Jehle J A
**Lacanobia oleracea GV**<br>*Lacanobia oleracea granulovirus*	Lacanobia oleracea GV a6 Herniou E A/Theze J
**Melanchra persicariae GV**	Melanchra persicariae GV 26 Herniou E A/Theze J
**Euplexia lucipara GV**	Euplexia lucipara GV 248 Herniou E A/Theze J
**Wiseana cervinata GV**	Wiseana cervinata GV 342 Herniou E A/Theze J
**Plutella xylostella GV**<br>*Plutella xylostella granulovirus*	Plutella xylostella GV PxGV C Spence R J<br>Plutella xylostella GV PxGV K Spence R J<br>Plutella xylostella GV PxGV T Spence R J<br>Plutella xylostella GV PxGV M Spence R J<br>Plutella xylostella GV K1 Hashimoto Y<br>Plutella xylostella GV PlxyGV-SA001 Adbulkadir F<br>Plutella xylostella GV SA Jukes M D<br>Plutella xylostella GV PLXYGV-SA001 Abdulkadir F<br>Plutella xylostella GV PlxyGV-SA001 Abdulkadir F
**Caloptilia theivora GV**	Caloptilia theivora GV Kouassi L N
**Hyphantria cunea GV**	Hyphantria cunea GV A18-3 A5-1 Jehle J A<br>Hyphantria cunea GV Hc1 Erbas Z
**Estigmene acrea GV**	Estigmene acrea GV M30-3 Jehle J A
**Spilosoma lutea GV**	Spilosoma lutea GV 126 Herniou E A/Theze J
**Xestia c-nigrum GV**<br>*Xestia c-nigrum granulovirus*<br>*Helicoverpa armigera granulovirus*	Xestia c-nigrum GV alpha-4 Goto C<br>Xestia c-nigrum GV Hayakawa T<br>Euxoa ochrogaster GV A24-1 Jehle J A<br>Scotogramma trifolii GV A26-3 Jehle J A<br>Hoplodrina ambigua GV M39-2 Lange M<br>Autographa gamma GV M39-3 Lange M<br>Helicoverpa armigera GV Harrison R L
**Trichoplusia ni GV**<br>*Trichoplusia ni granulovirus*<br>*Mythimna unipuncta granulovirus A*	Trichoplusia ni GV M10-5 Lange M<br>Trichoplusia ni GV LBIV-12 LBIV-12 Del Rincon-Castro M C<br>Pseudalatia unipuncta GV Hawaii Tanada Y<br>Pseudalatia unipuncta GV Hawaiin Li Y<br>Trichoplusia ni GV Akiyoshi D<br>Mythimna unipuncta GV KY410 Keathley C P
**Mocis latipes GV**	Mocis latipes GV Southern Brazil Ardisson-Araujo D M P
**Spodoptera frugiperda GV**<br>*Spodoptera frugiperda granulovirus*	Spodoptera frugiperda GV VG014 Cuartas P<br>Spodoptera frugiperda GV VG008 Cuartas P E<br>Spodoptera frugiperda GV A12-4 Jehle J A
**Spodoptera androgea GV**	Spodoptera androgea GV A25-7 Jehle J A
**Mythimna unipuncta GV**<br>*Mythimna unipuncta granulovirus B*	Pseudaletia sp GV 2 Rohrmann G F<br>Mythimna unipuncta GV MyunGV#8 Harrison R L
**Peridroma morpontora GV**	Peridroma morpontora GV A25-3 Jehle J A
**Spodoptera litura GV**<br>*Spodoptera litura granulovirus*	Spodoptera litura GV SlGV-K1 Wang Y
**Spodoptera littoralis GV**	Spodoptera littoralis GV 66 Herniou E A/Theze J
**Achaea janata GV**	Achaea janata GV Hyderabad Naveen Kumar P<br>Achaea janata GV Hyderabad Kumar P N
**Junonia coenia GV**	Junonia coenia GV 19 Herniou E A/Theze J
**Neodiprion sertifer NPV**<br>*Neodiprion sertifer Nucleopyhedrovirus*	Neodiprion sertifer NPV Garcia-Maruniak A
**Neodiprion abietis NPV**	Neodiprion abietis NPV Duffy S P
**Neodiprion lecontei NPV**<br>*Neodiprion lecontei Nucleopyhedrovirus*	Neodiprion lecontei NPV Lauzon H A
**Culex nigripalpus NPV**<br>*Culex nigripalpus Nucleopyhedrovirus*	Culex nigripalpus NPV Florida1997 Afonso C L

† Each line correspond to a phylogenetic species based on our analysis. The species currently recognized by the ICTV (2017 release) are presented in blue italics font
